# The effect of physical exercise on cardiopulmonary fitness in burn patients: A meta-analysis

**DOI:** 10.1371/journal.pone.0330301

**Published:** 2025-08-18

**Authors:** Da Huang, XiaoXiang Wan, Juan Xu

**Affiliations:** School of Physical Education, Jiangxi University of Technology, Nanchang, China; Iran University of Medical Sciences, IRAN, ISLAMIC REPUBLIC OF

## Abstract

**Objective:**

Cardiopulmonary dysfunction in burn patients is typically caused by both the burn injury and smoke inhalation. Normally presenting with symptoms such as dyspnea, decreased exercise tolerance, decreased maximal heart rate, and decreased arterial oxygen saturation. It has been demonstrated that physical activity helps to increase cardiorespiratory fitness. The goal of this study was to determine whether physical activity can provide additional benefits to the recovery of cardiorespiratory fitness in burn patients by examining research on the topic of physical activity’s ability to enhance cardiorespiratory fitness in burn patients.

**Methods:**

The electronic databases Web of Science, PubMed, Embase, and Cochrane Library were searched from their inception until August 30, 2024. To contrast the efficacy of conventional rehabilitation with the benefits of physical exercise in conjunction with it. Revman 5.4 software was employed to conduct a meta-analysis, with peak oxygen consumption serving as the primary outcome indicator and the 6-minute walking test (6MWT), forced vital capacity (FVC)%, forced expiratory volume in the first second (FEV1)%, max heart rate (HRmax), and resting heart rate (RHR) serving as secondary outcome indicators. The literature’s risk of bias was evaluated using the Cochrane Collaboration tool.

**Results:**

A total of 13 studies were incorporated into the meta-analysis, which involved 530 patients. The study results demonstrated that physical exercise combined with conventional rehabilitation significantly improved VO_2Peak_ (MD = 4.91, 95% CI: 3.52–6.29, P < 0.001), 6MWT (MD = 37.11, 95% CI: 11.72–62.51, P = 0.004), FVC% (MD = 6.54, 95% CI: 4.9–8.17, P < 0.001), and FEV_1_% (MD = 8.27, 95% CI: 7.39–9.14, P < 0.001) in burn patients compared to conventional rehabilitation. Furthermore, there was no significant difference in the change in resting heart rate (RHR) (MD = 2.04, 95% CI: −2.71–6.78; P = 0.40) between the physical activity group and the control group, but there was a significant difference in the change in maximum heart rate (HR-max) (MD = 6.27, 95% CI: 1.75–10.97, P = 0.007). The results of the subgroup analysis of VO2peak indicate that resistance training combined with aerobic exercise (MD = 5.47, 95% CI: 4.81–6.13, P < 0.001) is more effective than aerobic exercise alone. In terms of single exercise duration, exercise lasting longer than 60 minutes (MD = 6.32, 95% CI: 4.49–6.16, P < 0.001) is more effective than exercise lasting less than 60 minutes. The improvement effects in adult burn patients (MD = 6.09, 95% CI: 3.7–8.48, P < 0.001) were superior to those in pediatric burn patients. The improvement effects in severe burn patients (MD = 5.66, 95% CI: 4.2–7.12, P < 0.001) were superior to those in moderate burn patients.

**Conclusions:**

Our study results indicate that physical exercise, when combined with conventional rehabilitation, is more effective than conventional rehabilitation alone in improving cardiorespiratory fitness in burn patients. This is demonstrated by improvements in aerobic capacity, exercise performance, and respiratory function. The most effective approach may involve combining prolonged resistance with aerobic exercise. The certainty of the evidence assessed according to the GRADE guidelines was moderate and very low, with key factors such as publication bias, imprecision, and inconsistency leading to its downgrading.

## 1. Introduction

The burn itself (e.g., chest burns) and smoke inhalation are typically the main causes of cardiorespiratory impairment in burn patients [1]. Impaired cardiorespiratory fitness in burn patients is defined by damage to the inspiratory and expiratory muscles, lung dynamics, and instability of the alveolar-capillary membranes [2]. Lung injuries (such as inhalation injuries, acute respiratory distress syndrome, and lung-related diseases [3–5]) and cardiovascular system injuries (such as hypovolemic shock with myocardial depression and myocardial injuries [6–8]) are the most frequent cardiopulmonary injuries among burn patients. Studies have shown that patients with severe burn injuries often experience cardiorespiratory-related health issues, such as reduced exercise tolerance and significant changes in respiratory capacity [9,10]. Additionally, during exercise, burn patients exhibit more pronounced cardiorespiratory impairment. Compared to the healthy population, a condition that may persist for up to five years [11]. This impaired cardiorespiratory fitness negatively affects the quality of life and physical activity levels of burn patients [10]. Among all types of burns, thoracic burns have the greatest impact, leading to considerable changes in the physiological anatomy and function of the thorax. This results in decreased mobility of the thoracic spine joints, dyspnea, airway obstruction, disrupted ventilation-perfusion ratios, and reduced exercise tolerance [12,13]. Since patients often need to remain in bed for long periods during their recovery, this may further lead to insufficient physical activity, thereby increasing their risk of cardiovascular disease.

Physical exercise has been found effective in improving cardiorespiratory fitness in various populations, including patients with atrial fibrillation [14], AIDS [15], and cancer [16,17]. Previous meta-analyses have demonstrated that physical activity improves physical function in burn patients, as evidenced by increased lean body mass and higher peak torque of the knee extensor muscles [18–20]. Two of the studies [19,20] reported that resistance training had a positive effect on the lean body mass and lower limb knee extensor strength of burn patients. In a separate study [18], physical activity was found to have a beneficial impact on the peak oxygen uptake of burn patients. However, the residual cardiorespiratory fitness indices were not reported, such as exercise tolerance, pulmonary function, and cardiac function. Early experimental studies indicated that cardiopulmonary fitness can be effectively enhanced through a combination of resistance and aerobic exercise [21–24]. Despite this, there is ongoing debate regarding the impact of physical activity on respiratory capacity in burn patients. Some recent studies have suggested that rehabilitation interventions involving exercise may not lead to substantial improvements in respiratory capacity (e.g., FVC, FEV_1_) [25]. Conversely, some research has demonstrated that physical exercise in conjunction with traditional rehabilitation can enhance respiratory capacity more effectively than traditional physiotherapy alone (e.g., joint mobility training, scar treatment, and everyday activity training) [26,27]. In short, our findings from previous meta-analyses indicate that physical exercise has a positive effect on the enhancement of physical fitness in burn patients [18–20]. Few studies, nonetheless, have examined how exercise can enhance cardiorespiratory fitness, and there is ongoing debate regarding the impact of physical exercise on the improvement of pulmonary function.

Thus, the two primary goals of this meta-analysis are 1) to systematically evaluate the effects of physical exercise on cardiorespiratory fitness in burn patients, utilizing a comprehensive range of indicators based on the latest studies; to investigate how physical exercise impacts different burn groups; and to determine the optimal forms and dosages of exercise through subgroup analyses; and 2) to address the ongoing debate regarding the impact of physical exercise on pulmonary function, thereby providing valuable insights for clinical treatment and decision-making.

## 2. Methods

### 2.1. Search strategy

The meta-analysis was conducted in conformance with the Preferred Reporting Items for Systematic Reviews and Meta-analyses (PRISMA) [28] and was registered in the PROSPERO database (Registration number: CRD42024588013); specific details can be found in S1 Appendix.

The Web of Science, PubMed, Embase, and Cochrane Library literature databases were searched for the period from the construction of each database to 30 August 2024. The literature search was conducted to investigate the impact of physical exercise on cardiorespiratory fitness in a population of burn patients. The search was conducted using keywords and medical subject terms, and two authors (Da Huang and Juan Xu) employed the same search strategy. A review search was conducted by the author (XiaoXiang Wan). The details of the search process are provided in S2 Appendix.

### 2.2. Inclusion criteria

Based on the PICOS principles (population, intervention, comparison, outcome, study design), studies meeting the following criteria were included in this meta-analysis: 1) Population: burn patients; 2) Intervention: The experimental group received physical exercise combined with conventional rehabilitation. Physical exercise is an active behavior designed to enhance or maintain physical fitness and promote health through planned, organized, and repetitive physical activities. This includes programs such as resistance exercise, aerobic exercise, and yoga. The control group received conventional rehabilitation, which consists of daily activity training, joint mobility exercises, and scar management; 3) Comparison: The control group received conventional rehabilitation, standard care, or daily exercise without planned and supervised physical activity. 4) Outcomes: the primary outcome was VO_2Peak_, while secondary outcomes included 6MWT, FVC%, FEV1%, HRmax, and RHR; 5) Study design: randomized controlled trials or controlled trials.

### 2.3. Exclusion criteria

1) Animal experiments, medical reports, reviews, abstracts, conference proceedings, dissertations, or retracted papers. 2) Burn patient groups that include other diseases, such as diabetes or heart disease. 3) Non-controlled experiments. 4) Studies with incomplete data or data unavailable for extraction.

### 2.4. Data collection and extraction

The literature retrieved through the search was first screened for duplicates using EndNote X9 software. Subsequently, two researchers (Da Huang and Juan Xu) independently reviewed the titles and abstracts of the literature following the inclusion criteria to exclude irrelevant literature initially. The literature that satisfied the requirements was read in its entirety, and the necessary information for the study was extracted. Excel was employed to generate a data extraction form, which yielded the following information components: 1) publication details: first author, year of publication; 2) patient characteristics: sample size, gender, age, and burn area; 3) intervention and control details: intervention type, duration, frequency, and time; 4) outcome indicators; and 5) risk of bias assessment data.

### 2.5. Risk of bias

Two authors (Da Huang and Juan Xu) independently used the Cochrane risk of bias assessment tool to assess the methodological qualitative aspects of the included literature, and where disagreements arose, they were discussed with a third author (Xiao Xiang Wan) until consensus was reached. The assessment covered seven areas. 1) random sequence generation, 2) allocation concealment, 3) blinding of participants and personnel, 4) blinding of outcome assessments, 5) incomplete outcome data, 6) selective reporting, and 7) other bias.

### 2.6. Certainty of evidence

As per the GRADE guidelines, two authors (Da Huang and Juan Xu) independently extracted data and evaluated the quality of inclusion. If disagreements were identified, they were discussed with a third author (XiaoXiang Wan) until consensus was achieved. The GRADE system was used to evaluate the overall quality of the main outcome indicators [29].

### 2.7. Statistical analysis

Statistical analyses were conducted using RevMan software (version 5.4; Cochrane Collaboration, Oxford, UK). The outcome indicators included in the analysis were continuous variables, expressed as mean difference (MD) with 95% confidence intervals (CI) when test methods and indicator units were consistent. If differences existed, effect sizes were combined using standardized mean difference (SMD) with 95% CI. For meta-analysis, at least two studies were included per outcome, and heterogeneity was tested using the chi-square test and I^2^ statistic. A P-value of <0.05 indicated statistical significance. An I^2^ > 50% suggested heterogeneity among studies, prompting the use of a random-effects model, whereas I^2^ < 50% indicated no significant heterogeneity, leading to the use of a fixed-effects model. An I^2^ < 50% was considered acceptable between-study heterogeneity, while I^2^ > 50% prompted sensitivity analysis via case-by-case elimination. Heterogeneity was classified as none (I^2^ < 25%), mild (25%−50%), moderate (50%−75%), and severe (I^2^ > 75%). Publication bias was assessed using Stata 17 software.

## 3. Result

**Fig 1:**
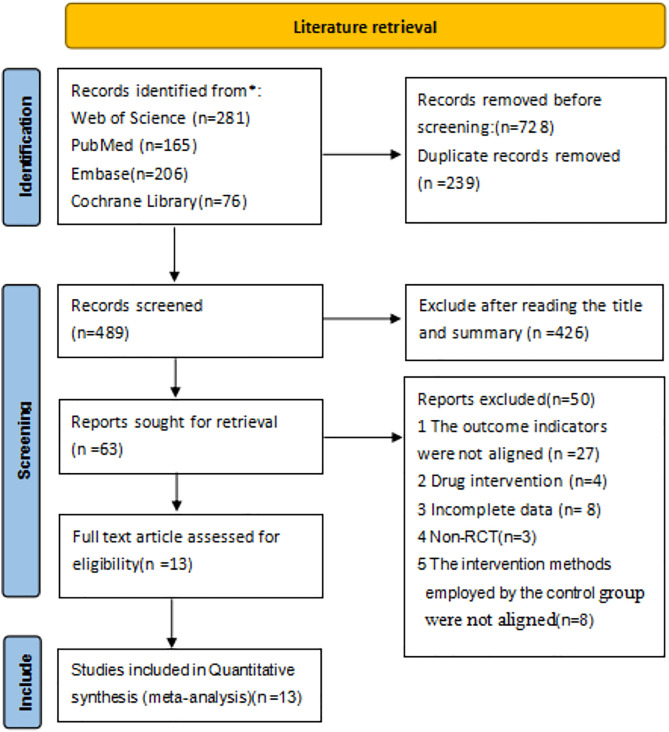
Flowchart of the database search following the PRISMA statement.

**Fig 2:**
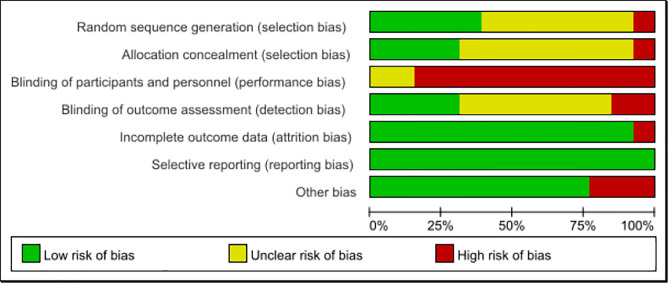
Overall risk of bias assessment.

**Fig 3:**
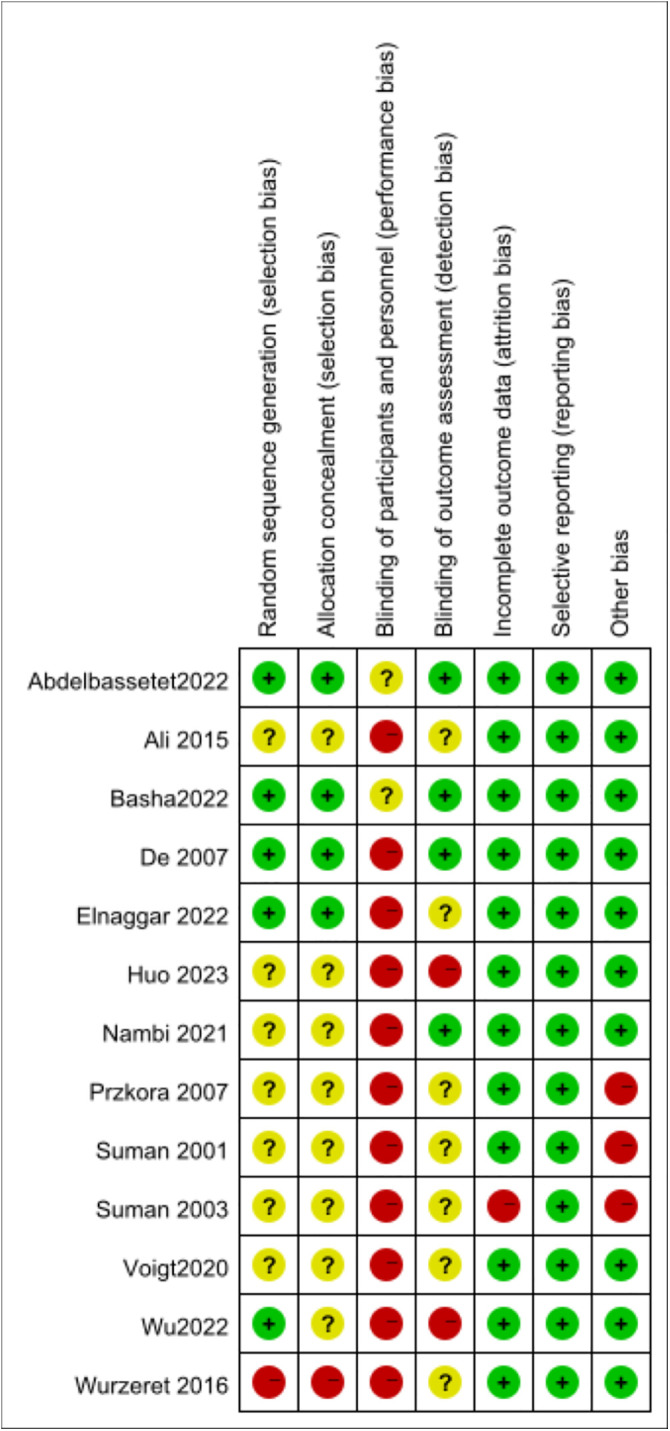
Risk of bias summary for randomized controlled trials.

In the beginning, 728 potentially possible papers were searched, 239 duplicates were excluded using EndNote X9 software, and 426 papers were excluded by reading the title and abstract. After reviewing the full text, 50 documents were excluded, and finally, 13 papers were included in the meta-analysis [21–23,26,27,30–37], as detailed in S3 and S4 Appendices. The screening process for the literature is illustrated in Fig 1, and the risk of bias assessment for the literature is presented in Figs 2 and 3, as well as in the S6 Appendix.

### 3.1. Trial and patient characteristics

A total of 13 papers [21–23,26,27,30–37] were incorporated into the meta-analysis, which involved 530 patients. Of these, 304 were in the experimental group, and 226 were in the control group. The following exercise types were included in the intervention: resistance exercise combined with aerobic exercise [21–23,34,35], resistance exercise [30], aerobic exercise [26,31–33,36,37], and yoga [27]. Table 1 displays specific information regarding the patients included in the study, the intervention program, and outcome indicators.

**Table 1:** Basic characteristics of the included studies.

Study	Sample Size	Sex	Age	Burn%	Hospital stay time (days)	Training program	Frequency of training per Week	Total intervention period (weeks)	Duration of a single intervention (minutes)	Outcome
Huo et al., [30] (2023, China)	EG:15 CG:17	EG:NA CG:NA	EG:9.6 ± 2.5 CG:8.5 ± 2.4	EG:28 CG:25	EG:26 ± 8 CG:24 ± 9	EG:Resistance CG:CR	12	3-5	NA	6MWT
Elnaggar et al., [31] (2022, Egypt)	EG:18 CG:18	EG:11/7 CG:8/10	EG:14.1 ± 2.7 CG:13.8 ± 2	EG:24.9 ± 5.3 CG:23.5 ± 4.7	EG:46.7 ± 11.5 CG:43.8 ± 7.7	EG:GAEx CG:CT	12	3	30-60 min	VO_2Peak_、HRmax
Basha et al., [32] (2022, Saudi)	EG:20 CG:20	EG:14/6 CG:12/8	EG:12.7 ± 1.56 CG:13.3 ± 1.29	EG:50 ± 4.61 CG:52 ± 5.51	EG:34.3 ± 4.23 CG:36.1 ± 3.63	EG:AE CG:CR	12	3	40min	VO_2Peak_
Wu et al., [33] (2022, China)	EG:20 CG:20	EG:13/7 CG:16/4	EG:39 ± 8 CG:45 ± 10	EG:44 ± 9 CG:47 ± 8	EG:59 ± 5 CG:56 ± 7	EG:Cycle ergometer CG:CR	8	7	35min	6MWT
Abdelbasset et al., [26] (2022, Egypt)	EG:15 CG:15	EG:9/6 CG:10/5	EG:13.8 ± 2.7 CG:14.2 ± 2.5	EG:NA CG:NA	EG:NA CG:NA	EG: Cycle ergometer CG: CR	8	3	30min	FVC%、FEV_1_、6MWT
Nambi et al., [27] (2021, Saudi)	EG:15 CG:15	EG:NA CG:NA	EG:37.5 ± 1.6 CG:38.4 ± 2.2	EG:11–25% CG:11–25%	EG:98.52 ± 5.8 CG:95.52 ± 6.2	EG: Yoga CG: CR	8	7	NA	FVC%、FEV_1_、6MWT
Voigt et al., [34] (2020, America)	EG:31 CG:14	EG:30/1 CG:14/0	EG:35 ± 11 CG:35 ± 9	EG:46 ± 11 CG:48 ± 19	EG:41 ± 34 CG:44 ± 40	EG: RS + AE CG: CR	12	3	>60min	VO_2Peak_
Wurzer et al., [35] (2016, America)	EG:82 CG:43	EG:59/23 CG:34/9	EG:12 ± 4 CG:12 ± 4	EG:56 ± 15 CG:54 ± 14	EG:38 ± 30 CG:33 ± 22	EG:RS + AE CG:CR	12	3	>60min	VO_2Peak_ RHR
Ali et al., [36] (2015, Egypt)	EG:15 CG:15	EG:NA CG:NA	EG:27.9 ± 7.3 CG:29.5 ± 6.6	EG:32.9 ± 6.6 CG:29.5 ± 5.1	EG:NA CG:NA	EG:AE CG:CR	12	3	>60min	VO_2Peak_
De et al., [37] (2007, America)	EG:11 EG:13 CG:11	EG:7/6 EG:10/1 CG:9/2	EG:35.4 ± 14.8 EG: 43.5 ± 8.9 CG: 34.9 ± 14.5	EG:16.8 ± 9.8 EG:19.5 ± 17.2 CG:21.6 ± 19.4	EG:NA EG:NA CG:NA	EG:AE CG:CR	12	3	30min	VO_2Peak_ HRmax RHR
Przkora et al., [21] (2007, America)	EG:17 CG:11	EG:13/4 CG:9/2	EG:10.9 ± 3.7 CG:11.8 ± 3.3	EG:55.6 ± 14.8 CG:53.4 ± 10.4	EG:NA CG:NA	EG:RS + AE CG:CR	12	3	>60min	VO_2Peak_
Suman et al., [22] (2003, America)	EG:13 CG:11	EG:10/3 CG:9/2	EG:10.5 ± 2.5 CG:10.8 ± 2.3	EG:58.5 ± 10.1 CG:59.4 ± 14.4	EG:38.4 ± 4.8 CG:35.8 ± 4.6	EG:RS + AE CG:CR	12	3	>60min	VO_2Peak_
Suman et al. [23] (2001, America)	EG:19 CG:16	EG:16/3 CG:12/4	EG:10.5 ± 0.92 CG:11.06 ± 1.2	EG:59.4 ± 3.30 CG:58.0 ± 4.42	EG:NA CG:NA	EG:RS + AE CG:CR	12	3	>60min	VO_2Peak_

EG: Experimental Group; CG: Control Group; RS: Resistance; CR: Conventional rehabilitation; AE: Aerobic Exercise; 6MWT: 6-minute walking test; VO_2Peak_: peak oxygen uptake; HRmax: max heart rate; RHR: resting heart rate; FVC:Forced Vital Capacity; FEV1:Forced Exprirtory Volume.

### 3.2. Study outcomes of VO_2Peak_

**Fig 4:**
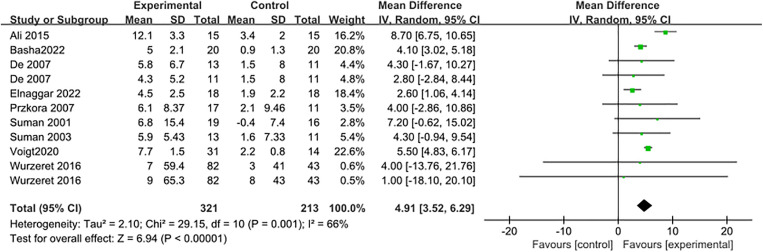
Forest plot of meta-analysis assessing the effect of physical activity on VO_2Peak_.

**Fig 5:**
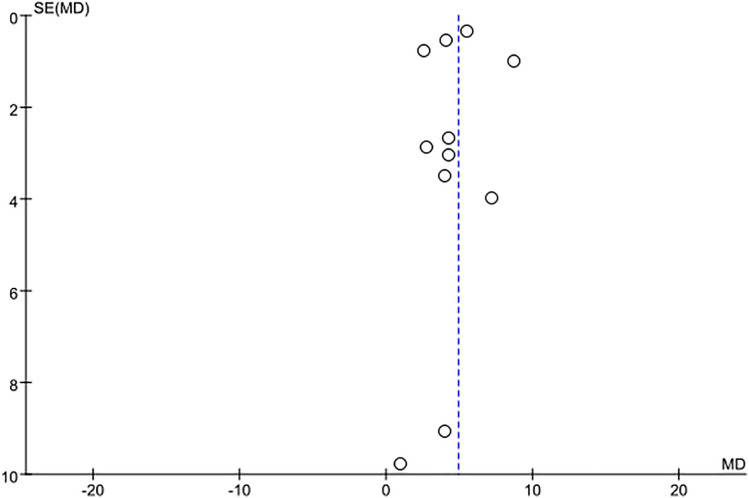
Funnel plot of VO_2Peak_ inclusion studies.

VO_2Peak_ was utilized as the primary index to analyze the effect of physical exercise on aerobic capacity in burn patients. Nine studies [21–23,31,32,34–37] reported VO_2Peak_ results for 398 patients. After combining the effect sizes (I^2^ = 66%, P = 0.001) using a random-effects model, the meta-analysis revealed that the experimental group’s VO_2Peak_ was significantly higher than that of the control group, with a mean difference of MD = 4.91 (95% CI: 3.52–6.29; P < 0.001), as shown in Fig 4. The funnel plot indicated that most studies were clustered at the top, and the two sides of the plot were not very symmetrical, suggesting a potential risk of publication bias, as shown in Fig 5. As indicated by Egger’s test, the results indicated that Prob > |z| = 0.4337 and P > 0.05, which implies the presence of publication bias.

### 3.3. Study outcomes of 6-minute walking test

**Fig 6:**
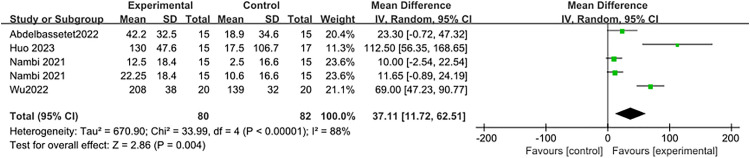
Forest plot of meta-analysis assessing the effect of physical activity on 6MWT.

The 6MWT was implemented to evaluate the impact of physical activity on the walking ability of burn patients. Four studies [26,27,30,33] reported results for the 6MWT. After combining the effect sizes (I^2^ = 88%, P < 0.001) using a random-effects model, the meta-analysis indicated that the experimental group covered a greater distance in the 6MWT compared to the control group, with a statistically significant difference (MD = 37.11, 95% CI: 11.72–62.51; P = 0.004), as shown in Fig 6).

### 3.4. Study outcomes of pulmonary function

**Fig 7:**
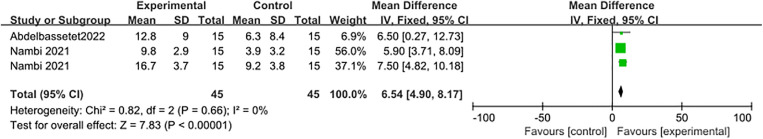
Forest plot of meta-analysis assessing the effect of physical activity on FVC%.

**Fig 8:**
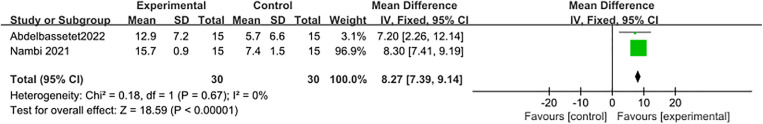
Forest plot of meta-analysis assessing the effect of physical activity on FEV _1_%.

Two randomized controlled trials [26,27] reported the results of FVC% after combining the effect sizes (I^2^ = 0%, P = 0.66), which were analyzed using a fixed-effects model. Meta-analysis revealed that the experimental group had a higher FVC% than the control group, and the difference in the change was statistically significant (MD = 6.54, 95% CI: 4.9–8.17; P < 0.001, as shown in Fig 7. According to the GRADE guidelines, publication bias and imprecision were two factors that led to downgrading, resulting in a low certainty of evidence for this outcome. Two randomized controlled trials [31,32] reported results for FEV1%, which were analyzed using a fixed-effects model after combining the effect sizes (I^2^ = 0%, P = 0.67). The meta-analysis indicated that FEV1% was higher in the experimental group compared to the control group, with a statistically significant difference (MD = 8.27, 95% CI: 7.39–9.14; P < 0.001), as shown in Fig 8.

### 3.5. Study outcomes of heart rate

**Fig 9:**
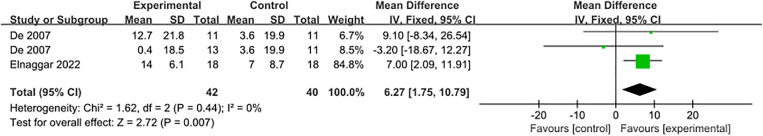
Forest plot of meta-analysis assessing the effect of physical activity on HRmax.

**Fig 10:**
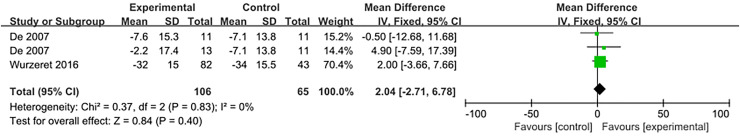
Forest plot of meta-analysis assessing the effect of physical activity on RHR.

A total of two studies [31,37] reported the effect of HRmax. After combining the effect sizes (I^2^ = 0%, P = 0.44) and analyzing the data using a fixed-effects model, the meta-analysis showed that HRmax was higher in the experimental group than in the control group before and after the intervention, with a statistically significant difference (MD = 6.27, 95% CI: 1.75–10.97, P = 0.007), as shown in Fig 9. According to the GRADE guidelines, publication bias and imprecision were factors for downgrading, resulting in a low certainty of evidence for this outcome. Additionally, two studies [35,37] reported the effect of RHR. After combining the effect sizes (I^2^ = 0%, P = 0.83) and analyzing the data using a fixed-effects model, the meta-analysis indicated that the change in RHR before and after the intervention in the experimental group was lower than that in the control group; however, this difference was not statistically significant (MD = 2.04, 95% CI: −2.71–6.78, P = 0.40), as shown in Fig 10.

### 3.6. Subgroup analysis of VO_2Peak_

Subgroup analyses based on age and heterogeneity tests after combining effect sizes revealed the following: for the children’s group, I^2^ = 0%, P = 0.76; for the adults’ group, I^2^ = 72%, P = 0.01. In the children’s group, the mean difference (MD) was 3.67 (95% CI: 2.81–4.53, P < 0.001), while in the adults’ group, MD was 6.09 (95% CI: 3.7–8.48; P < 0.001). Thus, the differences between both the child and adult groups compared to the control group were statistically significant, with no heterogeneity in the child group and moderate heterogeneity in the adult group (Table 2).

**Table 2:** Analysis of subgroups.

Outcome	Adjustment variables		n(ES)	Heterogeneous		Synergetic effect	
				P	*I*^*2*^ (%)	MD [95%CI]	P
VO_2Peak_	Total effect size		9	0.001	66	4.91 [3.52, 6.29]	<0.001
	Age	Children	6	0.76	0	3.67 [2.81, 4.53]	<0.001
		Adult	3	0.001	70	6.09 [3.7, 8.48]	<0.001
	Degree	Moderate	2	0.86	0	2.71 [1.27, 4.15]	0.002
		Severe	7	0.02	60	5.66 [4.2, 7.12]	<0.001
	ExerciseTime	>60min	6	0.12	41	6.32 [4.49, 8.16]	<0.001
		≤60 min	3	0.46	0	3.6 [2.73, 4.46]	<0.001
	Exercise Type	AE	4	<0.001	84	4.71 [2.26, 7.16]	<0.001
		RS + AE	5	0.98	0	5.47 [4.81, 6.13]	<0.001

Subgroup analyses based on burn severity showed that for the moderate group, I^2^ = 0, P = 0.86, and for the severe group, I^2^ = 60, P = 0.02. The moderate group had an MD of 2.71 (95% CI: 1.27–4.15, P < 0.001), while the severe group had an MD of 5.66 (95% CI: 4.2–7.12, P < 0.001). Both the moderate and severe groups were statistically significant compared to the control group, with no heterogeneity in the moderate group and moderate heterogeneity in the severe group (Table 2).

Subgroup analyses based on exercise duration showed that for the exercise time >60 min group, I^2^ = 41, P = 0.12, and for the exercise time ≤60 min group, I^2^ = 0, P = 0.46. The > 60 min group had an MD of 6.32 (95% CI: 4.49–6.16, P < 0.001), while the ≤ 60 min group had an MD of 3.6 (95% CI: 2.73–4.46, P < 0.001). Both groups were statistically significant compared to the control group, with no heterogeneity in the ≤ 60 min group and mild heterogeneity in the > 60 min group (Table 2).

Subgroup analyses based on the form of exercise showed that for the resistance combined with the aerobic exercise group, I^2^ = 0, P = 0.98, and for the aerobic exercise alone group, I^2^ = 84, P < 0.001. The resistance combined with the aerobic exercise group had an MD of 5.47 (95% CI: 4.81–6.13, P < 0.001), while the aerobic exercise alone group had an MD of 4.71 (95% CI: 2.26–7.16, P < 0.001). Both exercise forms were statistically significant compared to the control group, with no heterogeneity in the resistance combined with the aerobic exercise group and high heterogeneity in the aerobic exercise alone group (Table 2).

### 3.7. Sensitivity analysis

After excluding individual studies, the range of values for the combined effect size (MD) was found to be between 4.27 and 5.46, while I^2^ ranged from 39% to 69%, with p-values < 0.001 (Table 3). Notably, excluding the study by Ali (2015) [36] reduced the I^2^ from 66% to 39%. The sensitivity analysis indicated low sensitivity of the data, showing no significant changes to the meta-analysis results, which suggests stability and reliability in the findings.

**Table 3:** Combined effects of VO_2Peak_ after excluding single studies.

Study	MD	95%CI	P (synergetic effect)	*I*^*2*^ (%)
Ali 2015 [36]	4.27	3.2, 5.34	<0.001	39
Basha 2022 [32]	5.07	3.29, 6.68	<0.001	65
De 2007 [37]	5.01	3.57, 6.45	<0.001	68
De 2007 [37]	4.93	3.48, 6.38	<0.001	69
Elnaggar 2022 [31]	5.46	4.14, 6.78	<0.001	51
Przkora 2007 [21]	4.94	3.5, 6.38	<0.001	69
Suman 2001 [23]	4.84	3.41, 6.27	<0.001	69
Suman 2003 [22]	4.94	3.48, 6.4	<0.001	69
Voigt2020 [34]	4.72	2.82, 6.63	<0.001	64
Wurzer2016 [35]	4.93	3.52, 6.34	<0.001	69
Wurzer2016 [35]	4.91	3.5, 6.33	<0.001	69

### 3.8. Certainty of the evidence

The certainty of evidence for the analyzed results was evaluated using the GRADE software, as presented in S7 Appendix. Only the 6MWT showed a very low rating, with imprecision and inconsistency being the primary reasons for its downgrading. The other results were rated as moderate, with publication bias accounting for the downgrading of the VO_2Peak_ results and imprecision being the main factor for the downgrading of the remaining indicators.

## 4. Discussion

Based on existing research, our team used meta-analysis to explore the effects of physical exercise on the cardiopulmonary fitness of burn patients. Specifically, we investigated whether adding physical exercise to conventional rehabilitation could bring additional benefits to the cardiopulmonary fitness recovery of burn patients. In this analysis, we included 13 studies. Selecting VO_2peak_, closely related to cardiorespiratory fitness, as the primary outcome metric. Secondary outcome metrics included 6MWT, FVC%, FEV1%, HRmax, and RHR, which help address the controversy in this area. We found that cardiorespiratory fitness, encompassing aerobic capacity (VO_2Peak_), exercise capacity (6MWT), and pulmonary function (FVC%, FEV1%), can be effectively improved in burn patients through physical exercise. By analyzing the VO_2Peak_ subgroups, we discovered that regarding the effect of exercise, adult patients may benefit more than children from physical activity; patients with severe burns may show greater improvement than those with moderate burns; a combination of resistance and aerobic exercise may be more effective than aerobic exercise alone; and exercise sessions longer than 60 minutes may yield better results than those of 60 minutes or less. Additionally, sensitivity analysis indicated that the results of the VO_2Peak_ analysis exhibit stability and reliability.

VO_2peak_ refers to the maximum oxygen uptake that the body can achieve under specific conditions. It is widely used in assessments of aerobic capacity in burn patients and serves as a strong predictor of all-cause mortality [38]. We observed a statistically significant difference in the change in VO_2 peak_ between the experimental and control groups that were engaged in physical activity. This finding is consistent with the results of a previous meta-analysis [18]. The impact of burns on VO_2 peak_ was apparent in both adults and children. Children who were discharged from the hospital had a VO_2 peak_ that was 50% lower than that of an equivalent unburned population. Despite making a partial recovery over time, their VO_2 peak_ was still 20% lower than that of a healthy population at three years [39]. The data for adults is comparable, with a decrease in adults compared to standard data at 5–10 years [11,40]. VO_2 peak_ is influenced by a variety of factors, such as the pumping function of the heart, the ability of hemoglobin to transport oxygen, and the ability of the respiratory system to take up oxygen.

Regarding the heart’s pumping function, physical exercise has been shown to effectively improve resting heart structure in adults with burn injuries [41] and enhance cardiac output during exercise in children [42]. In terms of pulmonary function, we found that physical exercise enhanced the respiratory capacity of burn patients, as demonstrated by increases in FVC% and FEV1%. Our findings suggest that prolonged resistance combined with aerobic exercise may be the most effective approach for improving the condition of burn patients. In terms of duration, we believe that exercise sessions lasting more than 60 minutes are more beneficial, aligning with burn rehabilitation exercise guidelines that recommend over 150 minutes of exercise per week [43,44]. Additionally, a recent report highlights that exceeding 150 minutes of weekly exercise is a key factor in enhancing an individual’s VO_2 peak_ response [45]. We were unable to conduct subgroup analyses of exercise frequency versus intervention cycle. However, two previous experimental studies reported that VO_2 peak_ was improved, with a 12-week intervention cycle having a more significant effect than a 6-week intervention [46]. High-frequency interval training (more than three times per week) was more effective than low-frequency interval training (no more than two times per week) [47].

Concerning exercise intensity, a recent study concluded that a dose-response effect requires an intensity of more than 80% of peak heart rate [45]. The funnel plot exhibited asymmetry, and Egger’s test yielded a P-value of 0.0065 (<0.05), indicating the presence of publication bias. According to the GRADE guidelines, the certainty of evidence for VO_2Peak_ was rated as moderate, with publication bias being the primary factor for its downgrading.

The 6-minute walk test is commonly used in rehabilitation centers due to its simplicity and low cost [48]. Therefore, it is broadly employed in outpatient clinics and hospitals to evaluate patients with chronic diseases. It is also appropriate for the evaluation of functional mobility at the time of discharge from the hospital after individual specialist treatment of burns [49]. Previous meta-analyses [18–20] did not examine the 6MWT, while four of the studies we included focused on the short-term effects of physical activity on 6MWT performance in burn patients. The difference between the experimental and control groups was statistically significant, with an MD of 37.11 after pooling the effect sizes. This implies that physical activity is effective in enhancing the walking distance of burn patients. Additionally, enhanced walking function indicates an increase in aerobic capacity, which in turn enhances the patient’s capacity to conduct daily living activities.

Nevertheless, based on GRADE guidelines, the certainty of evidence for the 6MWT is very low, primarily due to inconsistency and imprecision. The high heterogeneity (I^2^ = 88%, P < 0.0001) likely stemmed from variability among the included groups, with two studies from China reporting more significant improvement effects. Furthermore, the small sample sizes of the included studies contributed to the downgrading of precision.

Pulmonary function impairment in burn patients is typically attributed to several factors, including direct thermal injury, smoke inhalation, and respiratory infections [1,50,51]. Among all types of burns, chest burns likely have the most significant impact on pulmonary function and are often the primary cause of restrictive lung disease [12]. FVC% and FEV1% are commonly used metrics for assessing the degree of airflow obstruction in response to lung ventilatory function [52]. We found that the experimental group and the control group exhibited a statistically significant difference in FVC% and FEV1% when we included two studies in a combined effect size analysis. This suggests that physical activity may enhance pulmonary ventilation function in burn patients. This was corroborated by improvements in other indices, including maximum voluntary ventilation (MVV), respiratory muscle strength maximal expiratory pressure (MEP) and maximal inspiratory pressure (MIP), diaphragmatic mobility (DM), minute ventilation (VE), ventilation equivalents (VEq/VO2), and respiratory exchange ratio (RER) [25,27,31]. A recent report discovered that pulmonary rehabilitation and expiratory muscle training (resistance combined with aerobic exercise) were more effective than pulmonary rehabilitation alone [53]. Consequently, physical exercise in conjunction with respiratory training appears to be more effective in enhancing pulmonary function. Further research will be required to demonstrate this. The certainty of evidence for both outcome metrics was rated as moderate according to the GRADE guidelines, with the insufficient sample sizes of the included studies leading to a downgrading of precision. HRmax was utilized to evaluate the relative cardiovascular stress response during exercise. Two studies were included in the pooled data analysis, revealing a statistically significant difference between the two groups. Nonetheless, both trials’ patient populations comprised both adults and children. More pertinent studies are required in the future to validate the impact of physical activity on the improvement of maximal heart rate in burn patients, as there were variations in the computation of maximal heart rate between children and adults. The certainty of evidence, as recommended by the GRADE guidelines, was rated as low. Despite physical activity having been demonstrated to enhance the resting cardiac structure of burn patients [41], our meta-analysis did not identify a statistically significant difference in the change in RHR among burn patients who engaged in physical activity. The certainty of evidence for both outcome metrics was assessed as moderate based on the GRADE guidelines, with insufficient sample sizes in the included studies leading to a downgrade in precision.

The following are the limitations of our study: 1) Due to the possibility of publishing and regional bias, we only searched four English journal databases and excluded gray literature and other databases in non-English languages. 2) The study’s groups comprised both adults and children, and they were widely dispersed. The results should be interpreted cautiously due to the structural differences in cardiovascular physiology between children and adults. 3) Only the six outcome indicators associated with cardiorespiratory fitness were examined in this meta-analysis; the other relevant outcome indicators were left out.

## 5. Conclusion

Our meta-analysis demonstrated that physical exercise, when combined with conventional rehabilitation, was more effective in improving cardiorespiratory fitness in burn patients than conventional rehabilitation alone. This was particularly evident in the enhancement of respiratory capacity, athletic ability, and aerobic capacity. By conducting subgroup analyses, we discovered that the most effective method of exercise for enhancing cardiorespiratory fitness is protracted resistance in conjunction with aerobic exercise. Physical exercise appears to be more beneficial for adults with burns and patients with severe burns. Based on our study’s findings and the recommendations from exercise guidelines, the optimal regimen for restoring cardiovascular fitness in burn patients is to exercise for more than 60 minutes at a time, at least three times a week, for 12 weeks, at 80% of maximum heart rate. According to the GRADE guidelines, the overall certainty of evidence is moderate and very low. Therefore, future randomized controlled trials with higher-quality responses related to respiratory and cardiac function are necessary to investigate further the effects of physical activity on cardiorespiratory fitness in burn patients.

## Supporting information

S1 Appendix PRISMA_2020_checklist. (DOCX)

S2 Appendix Search strategy. (DOC)

S3 Appendix Records removed before screening. (DOC)

S4 Appendix List of Included and Excluded Studies. (DOC)

S5 Appendix The data of meta-analysis. (XLS)

S6 Appendix Risk of bias. (XLS)

S7 Appendix GRADE judgements. (XLS)
