# Supplementary material for: The effect of physical exercise on cardiopulmonary fitness in burn patients: A meta-analysis
Source: PLoS One. 2025 Aug 18;20(8):e0330301. doi: 10.1371/journal.pone.0330301 (PMC12360598; doi:10.1371/journal.pone.0330301)
Supplement: S2 Appendix — (DOC) [file pone.0330301.s002.doc]

**Search strategy**

|  | **Pubed** |
| --- | --- |
| 1 | "burns"[MeSH Terms] |
| 2 | "burns"[MeSH Terms] |
| 3 | "burn"[Title/Abstract] OR "burns"[Title/Abstract] |
| 4 | #1 OR #2 OR #3 |
| 5 | “exercise”[MeSH Terms] |
| 6 | ( (((exercise[Title/Abstract])) OR (train[Title/Abstract])) OR (Physical activity[Title/Abstract])) OR (rehabilitation[Title/Abstract]) |
| 7 | #5 OR #6 |
| 8 | “randomized controlled trial”[Publication Type] |
| 9 | #4 AND #7 AND#8(n=165) |
| **Web of Science** | |
| 1 | (TS=(burns)) OR TS=(burn) |
| 2 | (((TS=(exercise)) OR TS=(train)) OR TS=(Physical activity)) OR TS=(rehabilitation) |
| 3 | ((TS=(randomized controlled trial)) OR TS=(clinical trial)) OR TS=(controlled clinical trial) |
| 4 | #1 AND #2 AND #3 (n=281) |
| **Embase** | |
| 1 | 'burn':ab,ti,kw OR 'burns':ab,ti,kw |
| 2 | 'exercise':ab,kw,ti OR 'train':ab,kw,ti OR 'physical activity':ab,kw,ti OR 'rehabilitation':ab,kw,ti |
| 3 | 'randomized controlled trial':ab,ti,kw OR 'clinical trial':kw,ti,ab OR 'controlled clinical trial':ab,kw,ti OR 'randomized':ab,kw,ti |
| 4 | #1 AND #2 AND #3 (n=206) |
| **Chochrane Library** | |
| **1** | MeSH descriptor: [Burns] explode all trees |
| **2** | "exercise":ab,kw,ti OR "train":ab,kw,ti OR "physical activity":ab,kw,ti OR"rehabilitation":ab,kw,ti |
| **3** | "randomized controlled trial":ab,ti,kw OR "clinical trial":kw,ti,ab OR "controlled clinical trial":ab,kw,ti OR "randomized":ab,kw,ti |
| **4** | #1 AND #2 AND #3 (n=76) |
