# Supplementary material for: The effect of physical exercise on cardiopulmonary fitness in burn patients: A meta-analysis
Source: PLoS One. 2025 Aug 18;20(8):e0330301. doi: 10.1371/journal.pone.0330301 (PMC12360598; doi:10.1371/journal.pone.0330301)
Supplement: S3 Appendix — (DOC) [file pone.0330301.s003.doc]

**S3 Table.** List of articles identified from different databases:

| Records removed before screening |
| --- |
| 1. Corrigendum to: Selection of Antiobesity Medications Based on Phenotypes Enhances Weight Loss: A Pragmatic Trial in an Obesity Clinic (Obesity, (2021), 29, 4, (662-671), 10.1002/oby.23120). *Obesity* 2021; **29**(9): 1565-1566.  2. Erratum: Corrigenda to “2021 SMA e-Conference Podium and Poster Presentations” (Journal of Science and Medicine in Sport (2021) 24(S1), (S1440244021003716)). *Journal of Science and Medicine in Sport* 2022; **25**(3): e1-e12.  3. ABAZARNEJAD E, FROUTAN R, AHMADABADI A, et al. Improving respiratory muscle strength and health status in burn patients: a randomized controlled trial. *Quality of Life Research* 2022; **31**(3): 769-776.  4. ABDEL-AAL NM, ALLAM NM, ELADL HM. Efficacy of whole-body vibration on balance control, postural stability, and mobility after thermal burn injuries: A prospective randomized controlled trial. *Clinical rehabilitation* 2021; **35**(11): 1555-1565.  5. ABDEL-AAL NM, ALLAM NM, ELADL HM. Efficacy of whole-body vibration on balance control, postural stability, and mobility after thermal burn injuries: A prospective randomized controlled trial. *Clinical rehabilitation* 2021; **35**(11): 1555-1565.  6. ABDEL-AAL NM, ALLAM NM, ELADL HM. Efficacy of whole-body vibration on balance control, postural stability, and mobility after thermal burn injuries: A prospective randomized controlled trial. *Clinical rehabilitation* 2021; **35**(11): 1555-1565.  7. ABDELBASSET WK, ABDELHALIM NM. Assessing the effects of 6 weeks of intermittent aerobic exercise on aerobic capacity, muscle fatigability, and quality of life in diabetic burned patients: randomized control study. *Burns : journal of the International Society for Burn Injuries* 2020; **46**(5): 1193‐1200.  8. ABDELBASSET WK, ABDELHALIM NM. Assessing the effects of 6 weeks of intermittent aerobic exercise on aerobic capacity, muscle fatigability, and quality of life in diabetic burned patients: Randomized control study. *Burns : journal of the International Society for Burn Injuries* 2020; **46**(5): 1193-1200.  9. ABDELBASSET WK, ABDELHALIM NM. Assessing the effects of 6 weeks of intermittent aerobic exercise on aerobic capacity, muscle fatigability, and quality of life in diabetic burned patients: Randomized control study. *Burns : journal of the International Society for Burn Injuries* 2020; **46**(5): 1193-1200.  10. ABDELBASSET WK, ABDELHALIM NM. Assessing the effects of 6 weeks of intermittent aerobic exercise on aerobic capacity, muscle fatigability, and quality of life in diabetic burned patients: Randomized control study. *Burns : journal of the International Society for Burn Injuries* 2020; **46**(5): 1193-1200.  11. ABDELBASSET WK, ELSAYED SH, NAMBI G, et al. Optimization of pulmonary function, functional capacity, and quality of life in adolescents with thoracic burns after a 2-month arm cycling exercise programme: A randomized controlled study. *Burns : journal of the International Society for Burn Injuries* 2022; **48**(1): 78-84.  12. ABDELBASSET WK, ELSAYED SH, NAMBI G, et al. Optimization of pulmonary function, functional capacity, and quality of life in adolescents with thoracic burns after a 2-month arm cycling exercise programme: A randomized controlled study. *Burns : journal of the International Society for Burn Injuries* 2022; **48**(1): 78-84.  13. ABDELBASSET WK, ELSAYED SH, NAMBI G, et al. Optimization of pulmonary function, functional capacity, and quality of life in adolescents with thoracic burns after a 2-month arm cycling exercise programme: a randomized controlled study. *Burns : journal of the International Society for Burn Injuries* 2022; **48**(1): 78‐84.  14. ABDELBASSET WK, ELSAYED SH, NAMBI G, et al. Response to Letter to the Editor on "Potential efficacy of sensorimotor exercise program on pain, proprioception, mobility, and quality of life in diabetic patients with foot burns: a 12-week randomized control study". *Burns : journal of the International Society for Burn Injuries* 2021; **47**(5): 1204‐1205.  15. ABDELBASSET WK, ELSAYED SH, NAMBI G, et al. Potential efficacy of sensorimotor exercise program on pain, proprioception, mobility, and quality of life in diabetic patients with foot burns: a 12-week randomized control study. *Burns : journal of the International Society for Burn Injuries* 2021; **47**(3): 587‐593.  16. ABDELBASSET WK, ELSAYED SH, NAMBI G, et al. Response to Letter to the Editor on “Potential efficacy of sensorimotor exercise program on pain, proprioception, mobility, and quality of life in diabetic patients with foot burns: A 12-week randomized control study”. *Burns : journal of the International Society for Burn Injuries* 2021; **47**(5): 1204-1205.  17. ABDELBASSET WK, ELSAYED SH, NAMBI G, et al. Potential efficacy of sensorimotor exercise program on pain, proprioception, mobility, and quality of life in diabetic patients with foot burns: A 12-week randomized control study. *Burns : journal of the International Society for Burn Injuries* 2021; **47**(3): 587-593.  18. ABDELBASSET WK, ELSAYED SH, NAMBI G, et al. Potential efficacy of sensorimotor exercise program on pain, proprioception, mobility, and quality of life in diabetic patients with foot burns: A 12-week randomized control study. *Burns : journal of the International Society for Burn Injuries* 2021; **47**(3): 587-593.  19. ABDELSATTAR AS, MAKKY S, NOFAL R, et al. Enhancement of wound healing via topical application of natural products: <i>In vitro</i> and <i>in vivo</i> evaluations. *Arab J Chem* 2022; **15**(6): 20.  20. ACOSTA A, CAMILLERI M, ABU DAYYEH B, et al. Selection of Antiobesity Medications Based on Phenotypes Enhances Weight Loss: A Pragmatic Trial in an Obesity Clinic. *Obesity* 2021; **29**(4): 662-671.  21. ACOSTA A, CAMILLERI M, ABU DAYYEH B, et al. Selection of Antiobesity Medications Based on Phenotypes Enhances Weight Loss: A Pragmatic Trial in an Obesity Clinic. *Obesity* 2021; **29**(4): 662-671.  22. AHUJA RB, GUPTA GK. A four arm, double blind, randomized and placebo controlled study of pregabalin in the management of post-burn pruritus. *Burns : journal of the International Society for Burn Injuries* 2013; **39**(1): 24-29.  23. AHUJA RB, GUPTA GK. A four arm, double blind, randomized and placebo controlled study of pregabalin in the management of post-burn pruritus. *Burns : journal of the International Society for Burn Injuries* 2013; **39**(1): 24-29.  24. AHUJA RB, GUPTA GK. A four arm, double blind, randomized and placebo controlled study of pregabalin in the management of post-burn pruritus. *Burns : journal of the International Society for Burn Injuries* 2013; **39**(1): 24-29.  25. ALI RR, SELIM AO, ABDEL GHAFAR MA, et al. Virtual reality as a pain distractor during physical rehabilitation in pediatric burns. *Burns : journal of the International Society for Burn Injuries* 2022; **48**(2): 303-308.  26. ALI ZA, ELADL HM, ABDELBASSET WK, et al. Inhalation injury in adult males: evaluation of the short-term efficacy of transcutaneous electrical acupoint stimulation on pulmonary functions and diaphragmatic mobility after burn: a double-blind randomized controlled study. *Burns : journal of the International Society for Burn Injuries* 2022; **48**(8): 1933‐1939.  27. ALI ZA, ELADL HM, ABDELBASSET WK, et al. Inhalation injury in adult males: Evaluation of the short-term efficacy of transcutaneous electrical acupoint stimulation on pulmonary functions and diaphragmatic mobility after burn: A double-blind randomized controlled study. *Burns : journal of the International Society for Burn Injuries* 2022; **48**(8): 1933-1939.  28. ALI ZA, ELADL HM, ABDELBASSET WK, et al. Inhalation injury in adult males: Evaluation of the short-term efficacy of transcutaneous electrical acupoint stimulation on pulmonary functions and diaphragmatic mobility after burn: A double-blind randomized controlled study. *Burns : journal of the International Society for Burn Injuries* 2022; **48**(8): 1933-1939.  29. ALI ZM, EL-REFAY BH, ALI RR. Aerobic exercise training in modulation of aerobic physical fitness and balance of burned patients. *Journal of physical therapy science* 2015; **27**(3): 585-589.  30. ALITO A, QUARTARONE A, LEONARDI G, et al. Brown adipose tissue human biomarkers: Which one fits best? A narrative review. *Medicine* 2022; **101**(48): 7.  31. ALLAM NM, BADAWY MM. Does High-Frequency Chest Wall Oscillation Have an Impact on Improving Pulmonary Function in Patients With Smoke Inhalation Injury? *Journal of burn care & research : official publication of the American Burn Association* 2021; **42**(2): 300-304.  32. AL-MOUSAWI AM, JESCHKE MG, CHINKES DL, et al. Effects of long-term combined oxandrolone and propranolol treatment following pediatric major burns. *Surgical Infections* 2009; **10**(2): 199-200.  33. AL-MOUSAWI AM, WILLIAMS FN, MLCAK RP, et al. Effects of exercise training on resting energy expenditure and lean mass during pediatric burn rehabilitation. *Journal of burn care & research* 2010; **31**(3): 400‐408.  34. AL-MOUSAWI AM, WILLIAMS FN, MLCAK RP, et al. Effects of exercise training on resting energy expenditure and lean mass during pediatric burn rehabilitation. *Journal of Burn Care and Research* 2010; **31**(3): 400-408.  35. AL-MOUSAWI AM, WILLIAMS FN, MLCAK RP, et al. Effects of exercise training on resting energy expenditure and lean mass during pediatric burn rehabilitation. *Journal of burn care & research : official publication of the American Burn Association* 2010; **31**(3): 400-408.  36. ALSHIEK J, GARCIA B, MINASSIAN V, et al. Clinical Consensus Statement: Vaginal Energy-Based Devices. *Obstetrical & Gynecological Survey* 2023; **78**(1): 23-25.  37. ALVES DD, MELO AKV, ALVES AF, et al. Safety and tolerability of cinnamaldehyde in orabase for oral candidiasis treatment: phase I clinical trial. *Clinical Oral Investigations* 2022; **26**(7): 4825-4833.  38. ANDY UU, JELOVSEK JE, CARPER B, et al. Impact of treatment for fecal incontinence on constipation symptoms. *American Journal of Obstetrics and Gynecology* 2020; **222**(6): 8.  39. ANTHONISSEN M, DALY D, JANSSENS T, et al. The effects of conservative treatments on burn scars: A systematic review. *Burns : journal of the International Society for Burn Injuries* 2016; **42**(3): 508-518.  40. ANTIRI EO, HORMENU T, ANSAH EW, et al. Efficacy of a physical activity regimen on visual outcomes among persons newly diagnosed with abnormal glucose tolerance: Study protocol for a pilot randomized controlled trial. 2024.  41. APAYDIN EA, MOHR DC, HAMILTON AB, et al. Differences in Burnout and Intent to Leave Between Women's Health and General Primary Care Providers in the Veterans Health Administration. *Journal of General Internal Medicine* 2022; **37**(10): 2382-2389.  42. ARSHAD Z, REHAN M, IQBAL T, et al. Treadmill Versus Overground Gait Training in Patients with Lower Limb Burn Injury: A Comparative Study. *Journal of burn care & research : official publication of the American Burn Association* 2023; **44**(5): 1150-1153.  43. ASHRAF U, MAQBOOL S, FATIMA T, et al. Comparison of the effect of Range of Motion exercises versus stretching techniques in prevention of burn contractures of upper limb; RCT- A Randomized clinical trial. *Pakistan Journal of Medical and Health Sciences* 2022; **16**(5): 54-56.  44. AULT P, PLAZA A, PARATZ J. Scar massage for hypertrophic burns scarring-A systematic review. *Burns : journal of the International Society for Burn Injuries* 2018; **44**(1): 24-38.  45. BADAWY MM, ALLAM NM. Impact of Adding Protein Supplementation to Exercise Training on Lean Body Mass and Muscle Strength in Burn Patients. *Journal of burn care & research : official publication of the American Burn Association* 2021; **42**(5): 968-974.  46. BAER L, SONG J, STANFORD K, et al. Effects of burn and disuse with resistance exercise on free fatty acids and fibroblast growth factor-21 in rats. *FASEB Journal* 2015; **29**.  47. BAER LA, SONG J, WOLF SE, et al. Effects of resistance exercise on caloric intake and body mass in rats following burn and disuse. *Journal of Burn Care and Research* 2015; **36**: S230.  48. BAER LA, SONG J, WOLF SE, et al. Effects of resistance exercise and daily insulin on body mass, food intake, fat mass and total hindlimb muscle mass in rats following burn and disuse. *Journal of Burn Care and Research* 2016; **37**: S207.  49. BARLETTA JF, PALMIERI TL, TOOMEY SA, et al. Management of Heat-Related Illness and Injury in the ICU: A Concise Definitive Review. *Critical Care Medicine* 2024; **52**(3): 362-375.  50. BARROW RE, DASU MR, FERRANDO AA, et al. Gene expression patterns in skeletal muscle of thermally injured children treated with oxandrolone. *Annals of surgery* 2003; **237**(3): 422-428.  51. BASHA MA, ABDEL-AAL NM, KAMEL FAH. Effects of Wii Fit Rehabilitation on Lower Extremity Functional Status in Adults With Severe Burns: a Randomized Controlled Trial. *Archives of physical medicine and rehabilitation* 2022; **103**(2): 289‐296.  52. BASHA MA, ABDEL-AAL NM, KAMEL FAH. Effects of Wii Fit Rehabilitation on Lower Extremity Functional Status in Adults With Severe Burns: A Randomized Controlled Trial. *Archives of physical medicine and rehabilitation* 2022; **103**(2): 289-296.  53. BASHA MA, ABDEL-AAL NM, KAMEL FAH. Effects of Wii Fit Rehabilitation on Lower Extremity Functional Status in Adults With Severe Burns: A Randomized Controlled Trial. *Archives of physical medicine and rehabilitation* 2022; **103**(2): 289-296.  54. BASHA MA, ABDEL-AAL NM, KAMEL FAH. Effects of Wii Fit Rehabilitation on Lower Extremity Functional Status in Adults With Severe Burns: A Randomized Controlled Trial. *Archives of physical medicine and rehabilitation* 2022; **103**(2): 289-296.  55. BASHA MA, ABOELNOUR NH, ALY SM, et al. Impact of Kinect-based virtual reality training on physical fitness and quality of life in severely burned children: a monocentric randomized controlled trial. *Annals of physical and rehabilitation medicine* 2022; **65**(1): 101471.  56. BASHA MA, ABOELNOUR NH, ALY SM, et al. Impact of Kinect-based virtual reality training on physical fitness and quality of life in severely burned children: A monocentric randomized controlled trial. *Annals of physical and rehabilitation medicine* 2022; **65**(1).  57. BASHA MA, ABOELNOUR NH, ALY SM, et al. Impact of Kinect-based virtual reality training on physical fitness and quality of life in severely burned children: A monocentric randomized controlled trial. *Annals of physical and rehabilitation medicine* 2022; **65**(1): 101471.  58. BASHA MA, ABOELNOUR NH, ALY SM, et al. Impact of Kinect-based virtual reality training on physical fitness and quality of life in severely burned children: A monocentric randomized controlled trial. *Annals of physical and rehabilitation medicine* 2022; **65**(1): 6.  59. BASHA MA, AZAB AR, ELNAGGAR RK, et al. Inspiratory muscle training impact on respiratory muscle strength, pulmonary function, and quality of life in children with chest burn: A randomized controlled trial. *Burns : journal of the International Society for Burn Injuries* 2024; **50**(7): 1916-1924.  60. BASHA MA, AZAB AR, ELNAGGAR RK, et al. Inspiratory muscle training impact on respiratory muscle strength, pulmonary function, and quality of life in children with chest burn: A randomized controlled trial. *Burns : journal of the International Society for Burn Injuries* 2024; **50**(7): 1916-1924.  61. BAYUO J, WONG FKY, CHUNG LYF. Effects of a nurse-led transitional burns rehabilitation programme (4Cs-TBuRP) for adult burn survivors: protocol for a randomised controlled trial. *Trials* 2021; **22**(1): 15.  62. BEAULIEU LD, BLANCHETTE AK, MERCIER C, et al. Efficacy, safety, and tolerability of bilateral transcranial direct current stimulation combined to a resistance training program in chronic stroke survivors: A double-blind, randomized, placebo-controlled pilot study. *Restorative Neurology and Neuroscience* 2019; **37**(4): 333-346.  63. BELVAL LN, CRAMER MN, MORALEZ G, et al. Interaction of Exercise Intensity and Simulated Burn Injury Size on Thermoregulation. *Medicine and science in sports and exercise* 2021; **53**(2): 367-374.  64. BENJAMIN NC, ANDERSEN CR, HERNDON DN, et al. The effect of lower body burns on physical function. *Burns : journal of the International Society for Burn Injuries* 2015; **41**(8): 1653-1659.  65. BERENDSEN S, VERDEGAAL LMA, VAN TRICHT MJ, et al. An old but still burning problem: Inter-rater reliability in clinical trials with antidepressant medication. *Journal of Affective Disorders* 2020; **276**: 748-751.  66. BEZUHLY M, FISH JS. Acute Burn Care. *Plastic and reconstructive surgery* 2012; **130**(2): 349E-358E.  67. BODEKAER M, FAURSCHOU A, PHILIPSEN PA, et al. Sun protection factor persistence during a day with physical activity and bathing. *Photodermatology, photoimmunology & photomedicine* 2008; **24**(6): 296-300.  68. BOHANON FJ, EDIONWE J, HESS C, et al. Effects of whole body vibration on bone of thermally injured children. *Journal of Burn Care and Research* 2015; **36**: S104.  69. BOLTON L. Evidence Corner: Taking Itch Seriously. *Wounds : a compendium of clinical research and practice* 2018; **30**(1): 27-28.  70. BORRIONE L, CAVENDISH BA, APARICIO LVM, et al. Home-Use Transcranial Direct Current Stimulation for the Treatment of a Major Depressive Episode: A Randomized Clinical Trial. *Jama Psychiatry* 2024; **81**(4): 329-337.  71. BOYER J, DASCOMBE B, EATHER N, et al. Feasibility of a teacher facilitated physical activity intervention for adolescents with disability: Findings from the Burn 2 Learn adapted pilot study. *Journal of Science and Medicine in Sport* 2021; **24**: S65-S66.  72. BRANSKI LK, HERNDON DN, BARROW RE, et al. Randomized Controlled Trial to Determine the Efficacy of Long-Term Growth Hormone Treatment in Severely Burned Children. *Annals of surgery* 2009; **250**(4): 514-523.  73. BRANSKI LK, HERNDON DN, BARROW RE, et al. Randomized controlled trial to determine the efficacy of long-term growth hormone treatment in severely burned children. *Annals of surgery* 2009; **250**(4): 514-522.  74. BRANSKI LK, HERNDON DN, BARROW RE, et al. Randomized controlled trial to determine the efficacy of long-term growth hormone treatment in severely burned children. *Annals of surgery* 2009; **250**(4): 514-523.  75. BRANSKI LK, HERNDON DN, BARROW RE, et al. Randomized controlled trial to determine the efficacy of long-term growth hormone treatment in severely burned children. *Annals of surgery* 2009; **250**(4): 514‐523.  76. BRAZIER A, LARSON E, XU Y, et al. 'Dear Doctor': a randomised controlled trial of a text message intervention to reduce burnout in trainee anaesthetists. *Anaesthesia* 2022; **77**(4): 405-415.  77. BURGESS JD, WATT KA, KIMBLE RM, et al. Knowledge of childhood burn risks and burn first aid: Cool Runnings. *Injury Prevention* 2019; **25**(4): 301-306.  78. BURNS J, BOOGAARD H, POLUS S, et al. Interventions to reduce ambient particulate matter air pollution and their effect on health. *Cochrane Database of Systematic Reviews* 2019; (5): 276.  79. CALVA V, CHOUINARD A, COUTURE MA, et al. Treating neuropathic pain in burn survivors: A case series. *Journal of Burn Care and Research* 2015; **36**: S103.  80. CANZONA M, HAYDON M, BUSCH M, et al. “Disconnected from the World:” Exploring How the COVID- 19 Pandemic Shaped Adolescent and Young Adult (AYA) Cancer Survivors' Daily Lives. *Psycho-Oncology* 2023; **32**: 71.  81. CAPARROS-LEFEBVRE D, DEWAILLY D. Preliminary PILOTE Study of cyproterone acetate for the treatment of aggressive behavior associated with severe dementia. *Rev Neurol* 2005; **161**(11): 1071-1078.  82. CAPONNETTO P, CAMPAGNA D, MAGLIA M, et al. Comparing the Effectiveness, Tolerability, and Acceptability of Heated Tobacco Products and Refillable Electronic Cigarettes for Cigarette Substitution (CEASEFIRE): Randomized Controlled Trial. *JMIR public health and surveillance* 2023; **9**: e42628.  83. CAPONNETTO P, CAMPAGNA D, MAGLIA M, et al. Comparing the Effectiveness, Tolerability, and Acceptability of Heated Tobacco Products and Refillable Electronic Cigarettes for Cigarette Substitution (CEASEFIRE): Randomized Controlled Trial. *JMIR public health and surveillance* 2023; **9**: e42628.  84. CAPONNETTO P, CAMPAGNA D, MAGLIA M, et al. Comparing the Effectiveness, Tolerability, and Acceptability of Heated Tobacco Products and Refillable Electronic Cigarettes for Cigarette Substitution (CEASEFIRE): Randomized Controlled Trial. *JMIR public health and surveillance* 2023; **9**: 14.  85. CATAPANO M, CATAPANO J, BORSCHEL G, et al. Effectiveness of Platelet-Rich Plasma Injections for Nonsurgical Management of Carpal Tunnel Syndrome: A Systematic Review and Meta-analysis of Randomized Controlled Trials. *Archives of physical medicine and rehabilitation* 2020; **101**(5): 897-906.  86. CATLEY MJ, O'CONNELL NE, BERRYMAN C, et al. Is Tactile Acuity Altered in People With Chronic Pain? A Systematic Review and Meta-analysis. *Journal of Pain* 2014; **15**(10): 985-1000.  87. CELIS MM, SUMAN OE, HUANG TT, et al. Effect of a supervised exercise and physiotherapy program on surgical interventions in children with thermal injury. *Journal of burn care & rehabilitation* 2003; **24**(1): 57‐61; discussion 56.  88. CELIS MM, SUMAN OE, HUANG TT, et al. Effect of a supervised exercise and physiotherapy program on surgical interventions in children with thermal injury. *The Journal of burn care & rehabilitation* 2003; **24**(1): 57-61; discussion 56.  89. CHA YH, URBANO D, PARISEAU N. Randomized Single Blind Sham Controlled Trial of Adjunctive Home-Based tDCS after rTMS for Mal De Debarquement Syndrome: Safety, Efficacy, and Participant Satisfaction Assessment. *Brain Stimulation* 2016; **9**(4): 539-546.  90. CHAO T, PARRY I, PALACKIC A, et al. The effects of short bouts of ergometric exercise for severely burned children in intensive care: A randomized controlled trial. *Clinical rehabilitation* 2022; **36**(8): 1052-1061.  91. CHAO T, PARRY I, PALACKIC A, et al. The effects of short bouts of ergometric exercise for severely burned children in intensive care: A randomized controlled trial. *Clinical rehabilitation* 2022; **36**(8): 1052-1061.  92. CHAO T, PARRY I, PALACKIC A, et al. The effects of short bouts of ergometric exercise for severely burned children in intensive care: A randomized controlled trial. *Clinical rehabilitation* 2022; **36**(8): 1052-1061.  93. CHAO T, PORTER C, HERNDON DN, et al. Propranolol and Oxandrolone Therapy Accelerated Muscle Recovery in Burned Children. *Medicine and science in sports and exercise* 2018; **50**(3): 427‐435.  94. CHAO T, PORTER C, HERNDON DN, et al. Propranolol and Oxandrolone Therapy Accelerated Muscle Recovery in Burned Children. *Medicine and science in sports and exercise* 2018; **50**(3): 427-435.  95. CHAO T, PORTER C, HERNDON DN, et al. Propranolol and Oxandrolone Therapy Accelerated Muscle Recovery in Burned Children. *Medicine and science in sports and exercise* 2018; **50**(3): 427-435.  96. CHAO T, PORTER C, HERNDON DN, et al. Propranolol and Oxandrolone Therapy Accelerated Muscle Recovery in Burned Children. *Medicine and science in sports and exercise* 2018; **50**(3): 427-435.  97. CHAUDHRY N, PANAGIOTI M, KHOSO AB, et al. Psychosocial and Physical Rehabilitation of Burn Survivors: A large multicentre cluster randomised controlled trial from Pakistan. *European Psychiatry* 2023; **66**: S906-S906.  98. CHENG ER, BAUER NS, DOWNS SM, et al. Parent Health Literacy, Depression, and Risk for Pediatric Injury. *Pediatrics* 2016; **138**(1): 9.  99. CHESTER JE, ROWNEKI M, VAN DOREN W, et al. Progression of intervention-focused research for Gulf War illness. *Military Med Res* 2019; **6**(1): 17.  100. CHO YS, JEON JH, HONG A, et al. The effect of burn rehabilitation massage therapy on hypertrophic scar after burn: A randomized controlled trial. *Burns : journal of the International Society for Burn Injuries* 2014; **40**(8): 1513-1520.  101. CHO YS, JEON JH, HONG A, et al. The effect of burn rehabilitation massage therapy on hypertrophic scar after burn: a randomized controlled trial. *Burns : journal of the International Society for Burn Injuries* 2014; **40**(8): 1513-1520.  102. CHO YS, JEON JH, HONG A, et al. The effect of burn rehabilitation massage therapy on hypertrophic scar after burn: A randomized controlled trial. *Burns : journal of the International Society for Burn Injuries* 2014; **40**(8): 1513-1520.  103. CHO YS, JOO SY, CUI H, et al. Effect of extracorporeal shock wave therapy on scar pain in burn patients: A prospective, randomized, single-blind, placebo-controlled study. *Medicine (United States)* 2016; **95**(32).  104. CHO YS, JOO SY, CUI H, et al. Effect of extracorporeal shock wave therapy on scar pain in burn patients: A prospective, randomized, single-blind, placebo-controlled study. *Medicine* 2016; **95**(32): e4575.  105. CHO YS, JOO SY, CUI H, et al. Effect of extracorporeal shock wave therapy on scar pain in burn patients A prospective, randomized, single-blind, placebo-controlled study. *Medicine* 2016; **95**(32): 7.  106. CHO YS, JOO SY, SEO CH. Effect of robot-assisted gait training on the biomechanical properties of burn scars: a single-blind, randomized controlled trial. *Burns and Trauma* 2022; **10**.  107. CHO YS, JOO SY, SEO CH. Effect of robot-assisted gait training on the biomechanical properties of burn scars: a single-blind, randomized controlled trial. *Burns & trauma* 2022; **10**: 9.  108. CHOI C, MUKOVOZOV I, JAZDAREHEE A, et al. Management of hypertrophic scars in adults: A systematic review and meta-analysis. *Australas J Dermatol* 2022; **63**(2): 172-189.  109. ÇINAR MA, BAYRAMLAR K, ERKILIC A, et al. Effect of three different exercise trainings on functional capacity in early stage severe burn patients: a randomized controlled trial. *Ulusal travma ve acil cerrahi dergisi [Turkish journal of trauma & emergency surgery: TJTES]* 2024; **30**(8): 562‐270.  110. ÇINAR MA, BAYRAMLAR K, ERKILIC A, et al. Effect of three different exercise trainings on functional capacity in early stage severe burn patients: A randomized controlled trial. *Ulusal Travma Ve Acil Cerrahi Dergisi-Turkish Journal of Trauma & Emergency Surgery* 2024; **30**(8): 562-570.  111. ÇıNAR MA, BAYRAMLAR K, ERKıLıC A, et al. Effect of three different exercise trainings on functional capacity in early stage severe burn patients: A randomized controlled trial. *Ulusal Travma ve Acil Cerrahi Dergisi* 2024; **30**(8): 562-570.  112. ÇıNAR MA, BAYRAMLAR K, ERKıLıC A, et al. Effect of three different exercise trainings on functional capacity in early stage severe burn patients: A randomized controlled trial. *Ulusal travma ve acil cerrahi dergisi = Turkish journal of trauma & emergency surgery : TJTES* 2024; **30**(8): 562-270.  113. ÇINAR MA, ERKILIç A. Effect of aerobic exercise on neutrophil-lymphocyte ratio, platelet-lymphocyte ratio, and lymphocyte-monocyte ratio in burn patients: a randomized controlled trial. *Journal of plastic, reconstructive & aesthetic surgery* 2024; **95**: 199‐206.  114. ÇINAR MA, ERKILIç A. Effect of aerobic exercise on neutrophil–lymphocyte ratio, platelet–lymphocyte ratio, and lymphocyte–monocyte ratio in burn patients: A randomized controlled trial. *Journal of Plastic, Reconstructive and Aesthetic Surgery* 2024; **95**: 199-206.  115. ÇINAR MA, ERKILIç A. Effect of aerobic exercise on neutrophil-lymphocyte ratio, platelet-lymphocyte ratio, and lymphocyte-monocyte ratio in burn patients: A randomized controlled trial. *Journal of plastic, reconstructive & aesthetic surgery : JPRAS* 2024; **95**: 199-206.  116. ÇINAR MA, ERKILIç A. Effect of aerobic exercise on neutrophil-lymphocyte ratio, platelet-lymphocyte ratio, and lymphocyte-monocyte ratio in burn patients: A randomized controlled trial. *Journal of Plastic Reconstructive and Aesthetic Surgery* 2024; **95**: 199-206.  117. CLARK DJ, CHATTERJEE SA, SKINNER JW, et al. Combining Frontal Transcranial Direct Current Stimulation With Walking Rehabilitation to Enhance Mobility and Executive Function: A Pilot Clinical Trial. *Neuromodulation* 2021; **24**(5): 950-959.  118. CLARKE A, COOPER C. Psychosocial rehabilitation after disfiguring injury or disease: investigating the training needs of specialist nurses. *J Adv Nurs* 2001; **34**(1): 18-26.  119. CLAYTON RP, WURZER P, ANDERSEN CR, et al. Effects of different duration exercise programs in children with severe burns. *Burns : journal of the International Society for Burn Injuries* 2017; **43**(4): 796‐803.  120. CLAYTON RP, WURZER P, ANDERSEN CR, et al. Effects of different duration exercise programs in children with severe burns. *Burns : journal of the International Society for Burn Injuries* 2017; **43**(4): 796-803.  121. CLAYTON RP, WURZER P, ANDERSEN CR, et al. Effects of different duration exercise programs in children with severe burns. *Burns : journal of the International Society for Burn Injuries* 2017; **43**(4): 796-803.  122. COGHLAN N, COPLEY J, APLIN T, et al. Patient Experience of Wearing Compression Garments Post Burn Injury: A Review of the Literature. *Journal of Burn Care & Research* 2017; **38**(4): 260-268.  123. COOPER MT. Common Painful Foot and Ankle Conditions A Review. *Jama-Journal of the American Medical Association* 2023; **330**(23): 2285-2294.  124. COSTA B, THORNTON M, GUEST E, et al. The effectiveness of interventions to improve psychosocial outcomes in parents of children with appearance-affecting health conditions: A systematic review. *Child Care Health and Development* 2021; **47**(1): 15-30.  125. COVEY MH, DUTCHER K, MARVIN JA, et al. Efficacy of continuous passive motion (CPM) devices with hand burns. *Journal of burn care & rehabilitation* 1988; **9**(4): 397‐400.  126. COVEY MH, DUTCHER K, MARVIN JA, et al. Efficacy of continuous passive motion (CPM) devices with hand burns. *Journal of Burn Care and Rehabilitation* 1988; **9**(4): 397-400.  127. COVEY MH, DUTCHER K, MARVIN JA, et al. Efficacy of continuous passive motion (CPM) devices with hand burns. *The Journal of burn care & rehabilitation* 1988; **9**(4): 397-400.  128. CRAIG TJ, HENAO MP. Advances in managing COPD related to α<sub>1</sub>-antitrypsin deficiency: An under-recognized genetic disorder. *Allergy* 2018; **73**(11): 2110-2121.  129. CUCUZZO NA, FERRANDO A, HERNDON DN. The effects of exercise programming vs traditional outpatient therapy in the rehabilitation of severely burned children. *Journal of burn care & rehabilitation* 2001; **22**(3): 214‐220.  130. CUCUZZO NA, FERRANDO A, HERNDON DN. The effects of exercise programming vs traditional outpatient therapy in the rehabilitation of severely burned children. *The Journal of burn care & rehabilitation* 2001; **22**(3): 214-220.  131. CUIJPERS MD, BAARTMANS MGA, JOOSTEN KFM, et al. The efficacy of therapeutic interventions on paediatric burn patients' height, weight, body composition, and muscle strength: A systematic review and meta-analysis. *Burns : journal of the International Society for Burn Injuries* 2024; **50**(6): 1437-1455.  132. CZECH O, WRZECIONO A, BATALíK L, et al. Virtual reality intervention as a support method during wound care and rehabilitation after burns: A systematic review and meta-analysis. *Complementary Therapies in Medicine* 2022; **68**: 9.  133. CZECH O, WRZECIONO A, BATALíK L, et al. Virtual reality intervention as a support method during wound care and rehabilitation after burns: A systematic review and meta-analysis. *Complementary Therapies in Medicine* 2022; **68**.  134. DAI YL, CHAI XM, ZHU N, et al. Analgesia effect of premixed nitrous oxide/oxygen during the rehabilitation after total knee arthroplasty: A study protocol for a randomized controlled trial. *Trials* 2019; **20**(1).  135. DAI YL, CHAI XM, ZHU N, et al. Analgesiaeffect of premixed nitrous oxide/oxygen during therehabilitation after total knee arthroplasty: a study protocol for a randomized controlled trial. *Trials* 2019; **20**: 8.  136. DALEY AJ, MCGEE E, BAYLISS S, et al. Effects of physical activity calorie equivalent food labelling to reduce food selection and consumption: systematic review and meta-analysis of randomised controlled studies. *Journal of epidemiology and community health* 2020; **74**(3): 269-275.  137. DARGAN D, KAZZAZI D, LIMNATITOU D, et al. Acute Management of Thermal Hand Burns in Adults: A 10-Year Review of the Literature. *Annals of plastic surgery* 2021; **86**(5): 517-531.  138. DARGAN D, KAZZAZI D, LIMNATITOU D, et al. Acute Management of Thermal Hand Burns in Adults A 10-Year Review of the Literature. *Annals of Plastic Surgery* 2021; **86**(5): 517-531.  139. DASGUPTA S, SANYAL S, GUPTA P, et al. A modification of split-skin graft. *Burns : journal of the International Society for Burn Injuries* 1997; **23**(6): 509-511.  140. DE LATEUR BJ, MAGYAR-RUSSELL G, BRESNICK MG, et al. Augmented exercise in the treatment of deconditioning from major burn injury. *Archives of physical medicine and rehabilitation* 2007; **88**(12 Suppl 2): S18‐23.  141. DE LATEUR BJ, MAGYAR-RUSSELL G, BRESNICK MG, et al. Augmented Exercise in the Treatment of Deconditioning From Major Burn Injury. *Archives of physical medicine and rehabilitation* 2007; **88**(12 SUPPL. 2): S18-S23.  142. DE LATEUR BJ, MAGYAR-RUSSELL G, BRESNICK MG, et al. Augmented exercise in the treatment of deconditioning from major burn injury. *Archives of physical medicine and rehabilitation* 2007; **88**(12 Suppl 2): S18-23.  143. DE LATEUR BJ, MAGYAR-RUSSELL G, BRESNICK MG, et al. Augmented exercise in the treatment of deconditioning from major burn injury. *Archives of physical medicine and rehabilitation* 2007; **88**(12): S18-S23.  144. DEGTIAREVA SA, SHMELEV EI, SMIRNOV SV, et al. [Stepwise treatment in patients with isolated thermal inhalation injury]. *Terapevticheskii arkhiv* 2013; **85**(8): 56-59.  145. DEMLING RH. Comparison of the anabolic effects and complications of human growth hormone and the testosterone analog, oxandrolone, after severe burn injury. *Burns : journal of the International Society for Burn Injuries* 1999; **25**(3): 215-221.  146. DEMLING RH. Comparison of the anabolic effects and complications of human growth hormone and the testosterone analog, oxandrolone, after severe burn injury. *Burns : journal of the International Society for Burn Injuries* 1999; **25**(3): 215-221.  147. DEMLING RH, DESANTI L. Oxandrolone, an anabolic steroid, significantly increases the rate of weight gain in the recovery phase after major burns. *Journal of Trauma - Injury, Infection and Critical Care* 1997; **43**(1): 47-51.  148. DEMLING RH, DESANTI L. Oxandrolone, an anabolic steroid, significantly increases the rate of weight gain in the recovery phase after major burns. *The Journal of trauma* 1997; **43**(1): 47-51.  149. DEMLING RH, DESANTI L. The rate of restoration of body weight after burn injury, using the anabolic agent oxandrolone, is not age dependent. *Burns : journal of the International Society for Burn Injuries* 2001; **27**(1): 46‐51.  150. DEMLING RH, DESANTI L. The rate of restoration of body weight after burn injury, using the anabolic agent oxandrolone, is not age dependent. *Burns : journal of the International Society for Burn Injuries* 2001; **27**(1): 46-51.  151. DEMLING RH, DESANTI L. Oxandrolone induced lean mass gain during recovery from severe burns is maintained after discontinuation of the anabolic steroid. *Burns : journal of the International Society for Burn Injuries* 2003; **29**(8): 793‐797.  152. DEMLING RH, DESANTI L. Oxandrolone induced lean mass gain during recovery from severe burns is maintained after discontinuation of the anabolic steroid. *Burns : journal of the International Society for Burn Injuries* 2003; **29**(8): 793-797.  153. DEMLING RH, DESANTI L. Oxandrolone induced lean mass gain during recovery from severe burns is maintained after discontinuation of the anabolic steroid. *Burns : journal of the International Society for Burn Injuries* 2003; **29**(8): 793-797.  154. DEMLING RH, ORGILL DP. The anticatabolic and wound healing effects of the testosterone analog oxandrolone after severe burn injury. *Journal of Critical Care* 2000; **15**(1): 12-17.  155. DEMLING RH, ORGILL DP. The anticatabolic and wound healing effects of the testosterone analog oxandrolone after severe burn injury. *J Crit Care* 2000; **15**(1): 12-17.  156. DENG AW, WEI D, RAN CF, et al. Psychological disorder in burnt patients. *Chinese Journal of Clinical Rehabilitation* 2005; **9**(16): 130-133.  157. DHATT PK, YASIN M, NICKELBURG SJ, et al. A successfully treated case of sulfamethoxazole-trimethoprim induced toxic epidermal necrolysis in an elderly patient. *Journal of the American Geriatrics Society* 2011; **59**: S64-S65.  158. DHAWAN S, ANDREWS R, KUMAR L, et al. A Randomized Controlled Trial to Assess the Effectiveness of Muscle Strengthening and Balancing Exercises on Chemotherapy-Induced Peripheral Neuropathic Pain and Quality of Life Among Cancer Patients. *Cancer Nursing* 2020; **43**(4): 269-280.  159. DICKINSON J, PERROS P. Thyroid-Associated Orbitopathy: Who and How to Treat. *Endocrinol Metabol Clin North Amer* 2009; **38**(2): 373-+.  160. DIETRICHS E. Carl Wilhelm Sem-Jacobsen Aerospace Neurophysiology and Deep Brain Stimulation Pioneer. *Neurology* 2022; **98**(5): 199-203.  161. DINYER TK, BYRD MT, GARVER MJ, et al. Low-Load vs. High-Load Resistance Training to Failure on One Repetition Maximum Strength and Body Composition in Untrained Women. *J Strength Cond Res* 2019; **33**(7): 1737-1744.  162. DIZON DS, SZCZEPANEK CM, PETRYLAK DP, et al. National impact of the COVID-19 pandemic on clinical trial staff attrition: Results of the SWOG Cancer Research Network Survey of Oncology Research Professionals. *Journal of Clinical Oncology* 2022; **40**(16).  163. DOWRAY S, SWARTZ JJ, BRAXTON D, et al. Potential effect of physical activity based menu labels on the calorie content of selected fast food meals. *Appetite* 2013; **62**: 173-181.  164. DRAZICH B, KUZMIK A, GALIK E, et al. Factors That Influence Physical Activity in Hospitalized Patients with Dementia. *Journal of the American Geriatrics Society* 2022; **70**(SUPPL 1): S223.  165. DUFFY SA, RONIS DL, WALTJE AH, et al. Protocol of a randomized controlled trial of sun protection interventions for operating engineers. *Bmc Public Health* 2013; **13**: 7.  166. DULHUNTY JM, BOOTS RJ, RUDD MJ, et al. Increased fluid resuscitation can lead to adverse outcomes in major-burn injured patients, but low mortality is achievable. *Burns : journal of the International Society for Burn Injuries* 2008; **34**(8): 1090-1097.  167. EBID AA, AHMED MT, EID MM, et al. Effect of whole body vibration on leg muscle strength after healed burns: A randomized controlled trial. *Burns : journal of the International Society for Burn Injuries* 2012; **38**(7): 1019-1026.  168. EBID AA, AHMED MT, MAHMOUD EID M, et al. Effect of whole body vibration on leg muscle strength after healed burns: a randomized controlled trial. *Burns : journal of the International Society for Burn Injuries* 2012; **38**(7): 1019-1026.  169. EBID AA, AHMED MT, MAHMOUD EID M, et al. Effect of whole body vibration on leg muscle strength after healed burns: A randomized controlled trial. *Burns : journal of the International Society for Burn Injuries* 2012; **38**(7): 1019-1026.  170. EBID AA, ATTALLA AF, IBRAHIM AR, et al. Effect of anti-gravity treadmill (Alter G) training on gait characteristics and postural stability in adult with healed burns: a single blinded randomized controlled trial. *Burns : journal of the International Society for Burn Injuries* 2024; **50**(1): 106‐114.  171. EBID AA, ATTALLA AF, IBRAHIM AR, et al. Effect of anti-gravity treadmill (Alter G) training on gait characteristics and postural stability in adult with healed burns: A single blinded randomized controlled trial. *Burns : journal of the International Society for Burn Injuries* 2024; **50**(1): 106-114.  172. EBID AA, ATTALLA AF, IBRAHIM AR, et al. Effect of anti-gravity treadmill (Alter G) training on gait characteristics and postural stability in adult with healed burns: A single blinded randomized controlled trial. *Burns : journal of the International Society for Burn Injuries* 2024; **50**(1): 106-114.  173. EBID AA, ATTALLA AF, IBRAHIM AR, et al. Effect of anti-gravity treadmill (Alter G) training on gait characteristics and postural stability in adult with healed burns: A single blinded randomized controlled trial. *Burns : journal of the International Society for Burn Injuries* 2024; **50**(1): 106-114.  174. EBID AA, EL-SHAMY SM, AMER MA. Effect of vitamin D supplementation and isokinetic training on muscle strength, explosive strength, lean body mass and gait in severely burned children: a randomized controlled trial. *Burns : journal of the International Society for Burn Injuries* 2017; **43**(2): 357‐365.  175. EBID AA, EL-SHAMY SM, AMER MA. Effect of vitamin D supplementation and isokinetic training on muscle strength, explosive strength, lean body mass and gait in severely burned children: A randomized controlled trial. *Burns : journal of the International Society for Burn Injuries* 2017; **43**(2): 357-365.  176. EBID AA, EL-SHAMY SM, AMER MA. Effect of vitamin D supplementation and isokinetic training on muscle strength, explosive strength, lean body mass and gait in severely burned children: A randomized controlled trial. *Burns : journal of the International Society for Burn Injuries* 2017; **43**(2): 357-365.  177. EBID AA, EL-SHAMY SM, AMER MA. Effect of vitamin D supplementation and isokinetic training on muscle strength, explosive strength, lean body mass and gait in severely burned children: A randomized controlled trial. *Burns : journal of the International Society for Burn Injuries* 2017; **43**(2): 357-365.  178. EBID AA, EL-SHAMY SM, DRAZ AH. Effect of isokinetic training on muscle strength, size and gait after healed pediatric burn: a randomized controlled study. *Burns : journal of the International Society for Burn Injuries* 2014; **40**(1): 97‐105.  179. EBID AA, EL-SHAMY SM, DRAZ AH. Effect of isokinetic training on muscle strength, size and gait after healed pediatric burn: A randomized controlled study. *Burns : journal of the International Society for Burn Injuries* 2014; **40**(1): 97-105.  180. EBID AA, EL-SHAMY SM, DRAZ AH. Effect of isokinetic training on muscle strength, size and gait after healed pediatric burn: a randomized controlled study. *Burns : journal of the International Society for Burn Injuries* 2014; **40**(1): 97-105.  181. EBID AA, EL-SHAMY SM, DRAZ AH. Effect of isokinetic training on muscle strength, size and gait after healed pediatric burn: A randomized controlled study. *Burns : journal of the International Society for Burn Injuries* 2014; **40**(1): 97-105.  182. EBID AA, OMAR MT, ABD EL BAKY AM. Effect of 12-week isokinetic training on muscle strength in adult with healed thermal burn. *Burns : journal of the International Society for Burn Injuries* 2012; **38**(1): 61-68.  183. ECCLESTON C, HEARN L, WILLIAMS ACD. Psychological therapies for the management of chronic neuropathic pain in adults. *Cochrane Database of Systematic Reviews* 2015; (10): 31.  184. EDGAR D, ZORZI LM, WAND B, et al. Prevention of neural hypersensitivity after acute upper limb burns: Development and pilot of a cortical training protocol. *Burns : journal of the International Society for Burn Injuries* 2011; **37**(4): 698-706.  185. EDGAR DW, FISH JS, GOMEZ M, et al. Local and Systemic Treatments for Acute Edema After Burn Injury: A Systematic Review of the Literature. *Journal of Burn Care & Research* 2011; **32**(2): 334-347.  186. EDIONWE J, HESS C, FERNANDEZ-RIO J, et al. Effects of whole-body vibration exercise on bone mineral content and density in thermally injured children. *Burns : journal of the International Society for Burn Injuries* 2016; **42**(3): 605‐613.  187. EDIONWE J, HESS C, FERNANDEZ-RIO J, et al. Effects of whole-body vibration exercise on bone mineral content and density in thermally injured children. *Burns : journal of the International Society for Burn Injuries* 2016; **42**(3): 605-613.  188. EDSTROM LE, ROBSON MC, MACCHIAVERNA JR, et al. Prospective randomized treatments for burned hands: Nonoperative vs. operative. Preliminary report. *Scandinavian Journal of Plastic and Reconstructive Surgery* 1979; **13**(1): 131-135.  189. EDWARDS L. New concepts in vulvodynia. *American Journal of Obstetrics and Gynecology* 2003; **189**(3): S24-S30.  190. EID MM, ABDELBASSET WK, ABDELATY FM, et al. Effect of physical therapy rehabilitation program combined with music on children with lower limb burns: a twelve-week randomized controlled study. *Burns : journal of the International Society for Burn Injuries* 2021; **47**(5): 1146‐1152.  191. EID MM, ABDELBASSET WK, ABDELATY FM, et al. Effect of physical therapy rehabilitation program combined with music on children with lower limb burns: A twelve-week randomized controlled study. *Burns : journal of the International Society for Burn Injuries* 2021; **47**(5): 1146-1152.  192. EID MM, ABDELBASSET WK, ABDELATY FM, et al. Effect of physical therapy rehabilitation program combined with music on children with lower limb burns: A twelve-week randomized controlled study. *Burns : journal of the International Society for Burn Injuries* 2021; **47**(5): 1146-1152.  193. EL AYADI A, PRASAI A, WANG Y, et al. Foxo3 modulates protein homeostasis and muscle cachexia postburn: Effects of androgen therapy and exercise. *Shock* 2018; **49**(6): 113.  194. ELIJAH I, EDELMAN LS, SABOURIN H, et al. The social milieu of burn injury and recovery: Using "Social Capital" as a framework for evaluating sex differences. *Journal of Burn Care & Research* 2008; **29**(1): 123-129.  195. ELNAGGAR RK, OSAILAN AM, ALSUBAIE SF, et al. Graded aerobic exercise (GAEx): an effective exercise regimen to improve cardio-respiratory fitness and physical and psychosocial functioning in children with burn sequelae of the chest. *Burns : journal of the International Society for Burn Injuries* 2022; **48**(2): 337‐344.  196. ELNAGGAR RK, OSAILAN AM, ALSUBAIE SF, et al. Graded aerobic exercise (GAEx): An effective exercise regimen to improve cardio-respiratory fitness and physical and psychosocial functioning in children with burn sequelae of the chest. *Burns : journal of the International Society for Burn Injuries* 2022; **48**(2): 337-344.  197. ELNAGGAR RK, SAMHAN AF, ELSHAFEY MA. Differential effects of extracorporeal shockwave therapy and botulinum toxin-a injection on postburn contractures and gait kinematics in burn children. *Journal of Burn Care and Research* 2020; **41**(3): 612-618.  198. ELNAGGAR RK, SAMHAN AF, ELSHAFEY MA. Differential Effects of Extracorporeal Shockwave Therapy and Botulinum Toxin-A Injection on Postburn Contractures and Gait Kinematics in Burn Children. *Journal of burn care & research : official publication of the American Burn Association* 2020; **41**(3): 612-618.  199. ELSHERBINY OE, EL FAHAR MH, WEHEIDA SM, et al. Effect of burn rehabilitation program on improving quality of life (QoL) for hand burns patients: a randomized controlled study. *European Journal of Plastic Surgery* 2018; **41**(4): 451-458.  200. ESHKALAK ZO, PARVIZY S, SEYEDFATEMI N, et al. The effectiveness of web-based training for parents on post-traumatic stress disorder in children. *Frontiers in psychology* 2024; **15**: 8.  201. ESSELMAN PC, THOMBS BD, MAGYAR-RUSSELL G, et al. Burn rehabilitation - State of the science. *American Journal of Physical Medicine & Rehabilitation* 2006; **85**(4): 383-413.  202. ETTENBERGER M, MAYA R, SALGADO-VASCO A, et al. The Effect of Music Therapy on Perceived Pain, Mental Health, Vital Signs, and Medication Usage of Burn Patients Hospitalized in the Intensive Care Unit: A Randomized Controlled Feasibility Study Protocol. *Frontiers in Psychiatry* 2021; **12**.  203. ETTENBERGER M, MAYA R, SALGADO-VASCO A, et al. The Effect of Music Therapy on Perceived Pain, Mental Health, Vital Signs, and Medication Usage of Burn Patients Hospitalized in the Intensive Care Unit: A Randomized Controlled Feasibility Study Protocol. *Frontiers in Psychiatry* 2021; **12**: 9.  204. ETULAIN J. Platelets in wound healing and regenerative medicine. *Platelets* 2018; **29**(6): 556-568.  205. EVERETT T, PARKER K, FISH J, et al. The Construction and Implementation of a Novel Postburn Pruritus Scale for Infants and Children Aged Five Years or Less: Introducing the Toronto Pediatric Itch Scale. *Journal of Burn Care & Research* 2015; **36**(1): 44-49.  206. FABRELLAS N, VIDAL A, AMAT G, et al. Nurse management of 'same day' consultation for patients with minor illnesses: results of an extended programme in primary care in Catalonia. *Journal of Advanced Nursing* 2011; **67**(8): 1811-1816.  207. FAUERBACH JA, GEHRKE AK, MASON ST, et al. Cognitive Behavioral Treatment for Acute Posttrauma Distress: A Randomized, Controlled Proof-of-Concept Study Among Hospitalized Adults With Burns. *Archives of physical medicine and rehabilitation* 2020; **101**(1): S16-S25.  208. FAUERBACH JA, GEHRKE AK, MASON ST, et al. Cognitive Behavioral Treatment for Acute Posttrauma Distress: A Randomized, Controlled Proof-of-Concept Study Among Hospitalized Adults With Burns. *Archives of physical medicine and rehabilitation* 2020; **101**(1s): S16-s25.  209. FAUERBACH JA, GEHRKE AK, MASON ST, et al. Cognitive Behavioral Treatment for Acute Posttrauma Check for updates Distress: A Randomized, Controlled Proof-of-Concept Study Among Hospitalized Adults With Burns. *Archives of physical medicine and rehabilitation* 2020; **101**(1): S16-S25.  210. FERGUSON SL, VOLL KV. Burn pain and anxiety: the use of music relaxation during rehabilitation. *The Journal of burn care & rehabilitation* 2004; **25**(1): 8-14.  211. FERNANDEZ S, TOBIN JN, CASSELLS A, et al. The counseling african americans to control hypertension (caatch) trial: baseline demographic, clinical, psychosocial, and behavioral characteristics. *Implement Sci* 2011; **6**: 13.  212. FIGUEIREDO FC, BAYLIS O, SALVADOR-CULLA B, et al. Outcomes of penetrating keratoplasty following ex vivo expanded Autologous Limbal Stem Cell Transplantation (ALSCT). *Investigative Ophthalmology and Visual Science* 2016; **57**(12): 891.  213. FIJAN S, FRAUWALLNER A, LANGERHOLC T, et al. Efficacy of Using Probiotics with Antagonistic Activity against Pathogens of Wound Infections: An Integrative Review of Literature. *Biomed Res Int* 2019; **2019**: 21.  214. FINNERTY CC, CAPEK KD, VOIGT C, et al. The P50 Research Center in Perioperative Sciences: How the investment by the National Institute of General Medical Sciences in team science has reduced postburn mortality. *Journal of Trauma and Acute Care Surgery* 2017; **83**(3): 532-542.  215. FISCHER M, CRAMER MN, HUANG M, et al. A Torso Burn Injury Does Not Exacerbate Thermoregulatory Strain During Exercise-Heat Stress While Wearing A Military Combat Uniform. *FASEB Journal* 2020; **34**(SUPPL 1).  216. FORD T, EDWARDS V, SHARKEY S, et al. Supporting teachers and children in schools: the effectiveness and cost-effectiveness of the incredible years teacher classroom management programme in primary school children: a cluster randomised controlled trial, with parallel economic and process evaluations. *Bmc Public Health* 2012; **12**: 16.  217. FREDERIX I, DENDALE P, BERGER J, et al. Comparison of two motion sensors for use in cardiac telerehabilitation. *Journal of Telemedicine and Telecare* 2011; **17**(5): 231-234.  218. FROUTAN R, SABERI A, AHMADABADI A, et al. The Effect of a Recreational Therapy Program on the Pain Anxiety and Quality of Life of Patients With Burn Injuries: A Randomized Clinical Trial. *Journal of Burn Care & Research* 2022; **43**(2): 381-388.  219. FUNG V, HO AL, SHAFFER J, et al. Use of Nintendo Wii Fit™ in the rehabilitation of outpatients following total knee replacement: a preliminary randomised controlled trial. *Physiotherapy* 2012; **98**(3): 183-188.  220. GALIK E, RESNICK B, LERNER N, et al. Function Focused Care for Assisted Living Residents With Dementia. *Gerontologist* 2015; **55**: S13-S26.  221. GARCIA-LARREA L, PERCHET C, HAGIWARA K, et al. At-Home Cortical Stimulation for Neuropathic Pain: a Feasibility Study with Initial Clinical Results. *Neurotherapeutics* 2019; **16**(4): 1198-1209.  222. GARRIDO-ARDILA EM, SANTOS-DOMíNGUEZ M, RODRíGUEZ-MANSILLA J, et al. A Systematic Review of the Effectiveness of Virtual Reality-Based Interventions on Pain and Range of Joint Movement Associated with Burn Injuries. *Journal of Personalized Medicine* 2022; **12**(8): 16.  223. GASTERATOS K, PAPAKONSTANTINOU M, MAN A, et al. Adjunctive Nonpharmacologic Interventions for the Management of Burn Pain: A Systematic Review. *Plastic and reconstructive surgery* 2022; **149**(5): 10.  224. GELLER AC, GLANZ K, SHIGAKI D, et al. Impact of skin cancer prevention on outdoor aquatics staff: the Pool Cool program in Hawaii and Massachusetts. *Prev Med* 2001; **33**(3): 155-161.  225. GERBER LH, BUSH H, HOLAVANAHALLI R, et al. A scoping review of burn rehabilitation publications incorporating functional outcomes. *Burns : journal of the International Society for Burn Injuries* 2019; **45**(5): 1005-1013.  226. GERBER LH, BUSH H, HOLAVANAHALLI R, et al. A scoping review of burn rehabilitation publications incorporating functional outcomes. *Burns : journal of the International Society for Burn Injuries* 2019; **45**(5): 1005-1013.  227. GERBER LH, DESHPANDE R, PRABHAKAR S, et al. Paucity of Clinical Practice Guidelines for the Rehabilitation of Burn Survivors. *American journal of physical medicine & rehabilitation* 2020; **99**(8): 739-751.  228. GERBER LH, DESHPANDE R, PRABHAKAR S, et al. Paucity of Clinical Practice Guidelines for the Rehabilitation of Burn Survivors. *American Journal of Physical Medicine & Rehabilitation* 2020; **99**(8): 739-751.  229. GHASEMI F. A randomized controlled trial of an adapted group cognitive-behavioral therapy for burned-out teachers. *Psychotherapy Research* 2023; **33**(4): 494-507.  230. GHEZELJEH TN, ARDEBILI FM, RAFII F. The effects of massage and music on pain, anxiety and relaxation in burn patients: Randomized controlled clinical trial. *Burns : journal of the International Society for Burn Injuries* 2017; **43**(5): 1034-1043.  231. GHEZELJEH TN, ARDEBILI FM, RAFII F, et al. The Effects of Music Intervention on Background Pain and Anxiety in Burn Patients: Randomized Controlled Clinical Trial. *Journal of Burn Care & Research* 2016; **37**(4): 226-234.  232. GIBSON L, STEPHENS-SHIELDS A, HUA S, et al. A Randomized Field Experiment Comparing Nutrition and Tax Salience Messages on Vending Machine Sales. *Obesity* 2022; **30**: 44.  233. GITTINGS PM, GRISBROOK TL, EDGAR DW, et al. Resistance training for rehabilitation after burn injury: A systematic literature review & meta-analysis. *Burns : journal of the International Society for Burn Injuries* 2018; **44**(4): 731-751.  234. GITTINGS PM, WAND BM, HINCE DA, et al. The efficacy of resistance training in addition to usual care for adults with acute burn injury: A randomised controlled trial. *Burns : journal of the International Society for Burn Injuries* 2021; **47**(1): 84-100.  235. GITTINGS PM, WAND BM, HINCE DA, et al. The efficacy of resistance training in addition to usual care for adults with acute burn injury: A randomised controlled trial. *Burns : journal of the International Society for Burn Injuries* 2021; **47**(1): 84-100.  236. GODLESKI M, HOLDEN MS, LUBY D, et al. Clinical Outcomes From a Foam Wedge Splinting Program for Axillary Contracture Prevention in the Intensive Care Unit. *Journal of Burn Care & Research* 2014; **35**(6): E379-E386.  237. GOETHALS J, CLAES K, FIERENS J, et al. The impact of the use of oxandrolone in the adult burn patient. *Intensive Care Medicine Experimental* 2019; **7**.  238. GOMEZ J, HOFFMAN HG, BISTRICKY SL, et al. The Use of Virtual Reality Facilitates Dialectical Behavior Therapy® "Observing Sounds and Visuals" Mindfulness Skills Training Exercises for a Latino Patient with Severe Burns: A Case Study. *Frontiers in psychology* 2017; **8**: 8.  239. GONçALVES N, CIOL MA, DANTAS RA, et al. A randomized controlled trial of an educational programme with telephone reinforcement to improve perceived health status of Brazilian burn victims at 6-month post discharge. *J Adv Nurs* 2016; **72**(10): 2508-2523.  240. GONçALVES N, CIOL MA, DANTAS RAS, et al. A randomized controlled trial of an educational programme with telephone reinforcement to improve perceived health status of Brazilian burn victims at 6-month post discharge. *Journal of Advanced Nursing* 2016; **72**(10): 2508-2523.  241. GONçALVES N, DANTAS RS, CIOL MA, et al. Randomized controlled clinical trial on telephone follow-up in rehabilitation of burn patients: impact on health status. *Quality of Life Research* 2014; **23**: 110-110.  242. GORDON MD, GOTTSCHLICH MM, HELVIG EI, et al. Review of evidenced-based practice for the prevention of pressure sores in burn patients. *Journal of Burn Care & Rehabilitation* 2004; **25**(5): 388-410.  243. GOTTLIEB M, COONEY R, HAAS M, et al. 340 Feel the Burn, While You Learn: The Impact of Exercise on Podcast Knowledge Acquisition and Retention. *Annals of Emergency Medicine* 2023; **82**(4): S149-S150.  244. GOTTSCHLICH MM, MAYES T, KHOURY J, et al. Differential effects of three vitamin D supplementation practices on clinical outcome postburn. *Journal of Burn Care and Research* 2011; **32**: S73.  245. GOUTOS I, ELDARDIRI M, KHAN AA, et al. Comparative evaluation of antipruritic protocols in acute burns. The emerging value of gabapentin in the treatment of burns pruritus. *Journal of burn care & research : official publication of the American Burn Association* 2010; **31**(1): 57-63.  246. GRISBROOK TL, GITTINGS PM, WOOD FM, et al. The effectiveness of session rating of perceived exertion to monitor resistance training load in acute burns patients. *Burns : journal of the International Society for Burn Injuries* 2017; **43**(1): 169‐175.  247. GRISBROOK TL, REID SL, EDGAR DW, et al. Exercise training to improve health related quality of life in long term survivors of major burn injury: A matched controlled study. *Burns : journal of the International Society for Burn Injuries* 2012; **38**(8): 1165-1173.  248. GUILLOT A, LEBON F, VERNAY M, et al. Effect of motor imagery in the rehabilitation of burn patients. *Journal of burn care & research : official publication of the American Burn Association* 2009; **30**(4): 686-693.  249. HAANSTRA TM, VAN BAAR ME, VAN LOEY NEE, et al. The burn centres outcome registry the netherlands (BORN): Development and first experiences. *Wound Repair and Regeneration* 2018; **26**(2): A22.  250. HäGELE FA, BüSING F, NAS A, et al. Appetite Control Is Improved by Acute Increases in Energy Turnover at Different Levels of Energy Balance. *Journal of Clinical Endocrinology & Metabolism* 2019; **104**(10): 4481-4491.  251. HAGHIGHI M, NABI BN, KHOSHRANG H, et al. The Effectiveness of Intravenous lidocaine in Burn Pain Relief: A Randomized Double-Blind Controlled Trial. *Crescent Journal of Medical and Biological Sciences* 2023; **10**(3): 110-115.  252. HAI BINH B, RAMIREZ P, MARTINEZ-PUIG D. A randomized, placebo-controlled study to evaluate efficacy and safety of a dietary supplement containing mucopolysaccharides, collagen type I and vitamin C for management of different tendinopathies. *Annals of the Rheumatic Diseases* 2014; **73**.  253. HAMMOND DC. Hypnosis in the treatment of anxiety- and stress-related disorders. *Expert review of neurotherapeutics* 2010; **10**(2): 263-273.  254. HAN B, RAN CF, HE YZ. Rehabilitative treatment for post-burn scar. *Chinese Journal of Clinical Rehabilitation* 2005; **9**(10): 180-182.  255. HAN K. Electric moxibustion for upper-limb lymphedema in breast cancer patients. *JAMS Journal of Acupuncture and Meridian Studies* 2020; **13**(2): 70.  256. HAN K, KWON O, PARK HJ, et al. Electronic Moxibustion for Breast Cancer-Related Lymphedema: A Pilot Clinical Trial. *Integr Cancer Ther* 2020; **19**: 12.  257. HAN T, KIM H, BAE J, et al. Neuromuscular pharmacodynamics of rocuronium in patients with major burns. *Anesthesia and analgesia* 2004; **99**(2): 386-392, table of contents.  258. HANNA R, BENSADOUN RJ, VANDER BEKEN S, et al. Outpatient Oral Neuropathic Pain Management with Photobiomodulation Therapy: A Prospective Analgesic Pharmacotherapy-Paralleled Feasibility Trial. *Antioxidants* 2022; **11**(3): 28.  259. HARDEE J, PORTER C, SIDOSSIS L, et al. Effect of early and late outpatient exercise training on muscle mass and protein kinetics in severely burned children. *FASEB Journal* 2014; **28**(1).  260. HARDEE JP, PORTER C, SIDOSSIS LS, et al. Early rehabilitative exercise training in the recovery from pediatric burn. *Medicine and science in sports and exercise* 2014; **46**(9): 1710-1716.  261. HARRé N, COVENEY A. School-based scalds prevention: reaching children and their families. *Health Educ Res* 2000; **15**(2): 191-202.  262. HEIDEN M, LYSKOV E, NAKATA M, et al. Evaluation of cognitive behavioural training and physical activity for patients with stress-related illnesses: A randomized controlled study. *Journal of Rehabilitation Medicine* 2007; **39**(5): 366-373.  263. HEIMBACH DM, WARDEN GD, LUTERMAN A, et al. Multicenter postapproval clinical trial of Integra<SUP>®</SUP> dermal regeneration template for burn treatment. *Journal of Burn Care & Rehabilitation* 2003; **24**(1): 42-48.  264. HERNDON D, CAPEK KD, ROSS E, et al. Reduced Postburn Hypertrophic Scarring and Improved Physical Recovery With Yearlong Administration of Oxandrolone and Propranolol. *Annals of surgery* 2018; **268**(3): 431-441.  265. HIRCHE C, ALMELAND SK, DHEANSA B, et al. Eschar removal by bromelain based enzymatic debridement (Nexobrid®) in burns: European consensus guidelines update. *Burns : journal of the International Society for Burn Injuries* 2020; **46**(4): 782-796.  266. HIRCHE C, CITTERIO A, HOEKSEMA H, et al. Eschar removal by bromelain based enzymatic debridement (Nexobrid®) in burns: An European consensus. *Burns : journal of the International Society for Burn Injuries* 2017; **43**(8): 1640-1653.  267. HOFFMAN HG, PATTERSON DR, SEIBEL E, et al. Virtual reality pain control during burn wound debridement in the hydrotank. *Clinical journal of pain* 2008; **24**(4): 299‐304.  268. HOFFMAN HG, PATTERSON DR, SEIBEL E, et al. Virtual reality pain control during burn wound debridement in the hydrotank. *Clinical Journal of Pain* 2008; **24**(4): 299-304.  269. HOFFMAN HG, PATTERSON DR, SEIBEL E, et al. Virtual reality pain control during burn wound debridement in the hydrotank. *The Clinical journal of pain* 2008; **24**(4): 299-304.  270. HOLAVANAHALLI RK, HELM PA, KOWALSKE KJ, et al. Effectiveness of Paraffin and Sustained Stretch in Treatment of Shoulder Contractures Following a Burn Injury. *Archives of physical medicine and rehabilitation* 2020; **101**(1): S42-S49.  271. HOLAVANAHALLI RK, HELM PA, KOWALSKE KJ, et al. Effectiveness of Paraffin and Sustained Stretch in Treatment of Shoulder Contractures Following a Burn Injury. *Archives of physical medicine and rehabilitation* 2020; **101**(1s): S42-s49.  272. HOLAVANAHALLI RK, HELM PA, KOWALSKE KJ, et al. Effectiveness of Paraffin and Sustained Stretch in Treatment of Shoulder Contractures Following a Burn Injury. *Archives of physical medicine and rehabilitation* 2020; **101**(1): S42-S49.  273. HOLBERT MD, DUFF J, WOOD F, et al. Barriers and co-designed strategies for the implementation of negative pressure wound therapy in acute pediatric burn care in Australia: A mixed method study. *Journal of Pediatric Nursing-Nursing Care of Children & Families* 2024; **77**: e520-e530.  274. HONARI S, CACERES M, ROMO M, et al. The role of a burn research coordinator: A guide for novice coordinators. *Journal of Burn Care and Research* 2016; **37**(2): 127-134.  275. HONARI S, CACERES M, ROMO M, et al. The Role of a Burn Research Coordinator: A Guide for Novice Coordinators. *Journal of Burn Care & Research* 2016; **37**(2): 127-134.  276. HONARI SE, CACERES M, GIBRAN NS. The role of a research coordinator (RC): A guide for novice coordinators. *Journal of Burn Care and Research* 2014; **35**: S132.  277. HONEA RA, VIDONI ED, MORRIS JK, et al. Aerobic exercise reduces hippocampal atrophy in individuals with early Alzheimer's disease. *Alzheimer's and Dementia* 2014; **10**: P303.  278. HUNTER AL, UNOSSON J, BOSSON JA, et al. Effect of wood smoke exposure on vascular function and thrombus formation in healthy fire fighters. *Part Fibre Toxicol* 2014; **11**: 62.  279. HUNTER AL, UNOSSON J, BOSSON JA, et al. Effect of wood smoke exposure on vascular function and thrombus formation in healthy fire fighters. *Particle and fibre toxicology* 2014; **11**: 62.  280. HUO T, RUAN JJ, JIANG MJ, et al. [Prospective study on the effects of resistance training with elastic band at home on muscle function and walking ability of severely burned children]. *Zhonghua Shao Shang Yu Chuang Mian Xiu Fu Za Zhi* 2023; **39**(12): 1131-1139.  281. IBRAHIM ZM, ALI OI, MOAWD SA, et al. Low vibrational training as an additional intervention for postural balance, balance confidence and functional mobility in type 2 diabetic patients with lower limb burn injury: A randomized clinical trial. *Diabetes, Metabolic Syndrome and Obesity* 2021; **14**: 3617-3626.  282. IBRAHIM ZM, ALI OI, MOAWD SA, et al. Low Vibrational Training as an Additional Intervention for Postural Balance, Balance Confidence and Functional Mobility in Type 2 Diabetic Patients with Lower Limb Burn Injury: A Randomized Clinical Trial. *Diabetes Metabolic Syndrome and Obesity-Targets and Therapy* 2021; **14**: 3617-3626.  283. INTISO D, BASCIANI M, SANTAMATO A, et al. Botulinum Toxin Type A for the Treatment of Neuropathic Pain in Neuro-Rehabilitation. *Toxins* 2015; **7**(7): 2454-2480.  284. IPAKTCHI K, ARBABI S. Advances in burn critical care. *Critical Care Medicine* 2006; **34**(9): S239-S244.  285. ISMAIL M, ALSUBHEEN SA, LOUCKS-ATKINSON A, et al. Multiple propane gas burn rates procedure to determine accuracy and linearity of indirect calorimetry systems: an experimental assessment of a method. *PeerJ* 2022; **10**: 19.  286. JACOBS PL, BURNS P. Acute enhancement of lower-extremity dynamic strength and flexibility with whole-body vibration. *Journal of Strength and Conditioning Research* 2009; **23**(1): 51-57.  287. JAGNOOR J, LUKASZYK C, FRASER S, et al. Rehabilitation practices for burn survivors in low and middle income countries: A literature review. *Burns : journal of the International Society for Burn Injuries* 2018; **44**(5): 1052-1064.  288. JAISHREE, BHOWMIK S. Letter to the Editor on: "Optimization of pulmonary function, functional capacity, and quality of life in adolescents with thoracic burns after a 2-month arm cycling exercise programme: a randomized controlled study". *Burns : journal of the International Society for Burn Injuries* 2022; **48**(5): 1268‐1269.  289. JAISHREE, BHOWMIK S. Letter to the Editor on: “Optimization of pulmonary function, functional capacity, and quality of life in adolescents with thoracic burns after a 2-month arm cycling exercise programme: A randomized controlled study”. *Burns : journal of the International Society for Burn Injuries* 2022; **48**(5): 1268-1269.  290. JAISHREE, BHOWMIK S. Letter to the Editor on: "Optimization of pulmonary function, functional capacity, and quality of life in adolescents with thoracic burns after a 2-month arm cycling exercise programme: A randomized controlled study". *Burns : journal of the International Society for Burn Injuries* 2022; **48**(5): 1268-1269.  291. JAMES A, ADAMS-HUET B, SHAH M. Menu labels displaying the kilocalorie content or the exercise equivalent: effects on energy ordered and consumed in young adults. *American journal of health promotion : AJHP* 2015; **29**(5): 294-302.  292. JAMES A, ADAMS-HUET B, SHAH M. Menu labels displaying the kilocalorie content or the exercise equivalent: effects on energy ordered and consumed in young adults. *Am J Health Promot* 2015; **29**(5): 294-302.  293. JANDA M, YOUL P, MARSHALL AL, et al. The HealthyTexts study: a randomized controlled trial to improve skin cancer prevention behaviors among young people. *Contemp Clin Trials* 2013; **35**(1): 159-167.  294. JANG KU, CHOI JS, MUN JH, et al. Multi-axis shoulder abduction splint in acute burn rehabilitation: a randomized controlled pilot trial. *Clinical rehabilitation* 2015; **29**(5): 439-446.  295. JANG KU, CHOI JS, MUN JH, et al. Multi-axis shoulder abduction splint in acute burn rehabilitation: a randomized controlled pilot trial. *Clinical rehabilitation* 2015; **29**(5): 439-446.  296. JANG KU, CHOI JS, MUN JH, et al. Multi-axis shoulder abduction splint in acute burn rehabilitation: a randomized controlled pilot trial. *Clinical rehabilitation* 2015; **29**(5): 439-446.  297. JEFFS D, DORMAN D, BROWN S, et al. Effect of Virtual Reality on Adolescent Pain During Burn Wound Care. *Journal of Burn Care & Research* 2014; **35**(5): 395-408.  298. JEPHCOTT C, GRUMMET J, NGUYEN N, et al. A review of the safety and efficacy of inhaled methoxyflurane as an analgesic for outpatient procedures. *British Journal of Anaesthesia* 2018; **120**(5): 1040-1048.  299. JERKIC M, LEUNG CH, KHAN Z, et al. Remote ischemic conditioning in posttraumatic hemorrhagic shock patients: Potential role of irisin. *Shock* 2020; **53**: 122.  300. JESCHKE MG, FINNERTY CC, SUMAN OE, et al. The effect of Oxandrolone on the endocrinologic, inflammatory, and hypermetabolic responses during the acute phase postburn. *Annals of surgery* 2007; **246**(3): 351-362.  301. JIANG JR, YEN SY, CHIEN JY, et al. Predicting weaning and extubation outcomes in long-term mechanically ventilated patients using the modified Burns Wean Assessment Program scores. *Respirology* 2014; **19**(4): 576-582.  302. JIN DY, HALVARI H, MAEHLE N, et al. Self-tracking behaviour in physical activity: a systematic review of drivers and outcomes of fitness tracking. *Behaviour & Information Technology* 2022; **41**(2): 242-261.  303. JO JH, JANG Y, CHUNG G, et al. Long-term efficacy and patient satisfaction of pulsed radiofrequency therapy in temporomandibular disorders A randomized controlled trial. *Medicine* 2021; **100**(52): 9.  304. JOHNSTON MV, SHERER M, WHYTE J. Applying evidence standards to rehabilitation research. *American journal of physical medicine & rehabilitation* 2006; **85**(4): 292‐309.  305. JOHNSTON MV, SHERER M, WHYTE J. Applying evidence standards to rehabilitation research. *American Journal of Physical Medicine and Rehabilitation* 2006; **85**(4): 292-309.  306. JOHNSTON MV, SHERER M, WHYTE J. Applying evidence standards to rehabilitation research. *American Journal of Physical Medicine & Rehabilitation* 2006; **85**(4): 292-309.  307. JOO SY, CHO YS, LEE SY, et al. Effects of virtual reality-based rehabilitation on burned hands: A prospective, randomized, single-blind study. *Journal of clinical medicine* 2020; **9**(3).  308. JOO SY, LEE SY, CHO YS, et al. Effects of robot-assisted gait training in patients with burn injury on lower extremity: A single-blind, randomized controlled trial. *Journal of clinical medicine* 2020; **9**(9): 1-12.  309. JOO SY, LEE SY, CHO YS, et al. Effects of Robot-Assisted Gait Training in Patients with Burn Injury on Lower Extremity: A Single-Blind, Randomized Controlled Trial. *Journal of clinical medicine* 2020; **9**(9): 11.  310. JOSUTTIS D, KRUSE M, PLETTIG P, et al. Prehospital treatment of severely burned patients: a retrospective analysis of patients admitted to the Berlin burn centre. *Scand J Trauma Resusc Emerg Med* 2024; **32**(1): 10.  311. KABLE TJ, LEAHY AA, SMITH JJ, et al. Time-efficient physical activity intervention for older adolescents with disability: rationale and study protocol for the Burn 2 Learn adapted (B2La) cluster randomised controlled trial. *Bmj Open* 2022; **12**(8): 11.  312. KALICHMAN L, VERED E, VOLCHEK L. Relieving Symptoms of Meralgia Paresthetica Using Kinesio Taping: A Pilot Study. *Archives of physical medicine and rehabilitation* 2010; **91**(7): 1137-1139.  313. KAMAL AM, FATHY H. Psychiatric assessment of disfigured burn patients following cognitive behavioral therapy program. *Egyptian Journal of Neurology, Psychiatry and Neurosurgery* 2013; **50**(1): 19-24.  314. KAMEL FAH, BASHA MA. Effects of Virtual Reality and Task-Oriented Training on Hand Function and Activity Performance in Pediatric Hand Burns: A Randomized Controlled Trial. *Archives of physical medicine and rehabilitation* 2021; **102**(6): 1059-1066.  315. KAMEL FAH, BASHA MA. Effects of Virtual Reality and Task-Oriented Training on Hand Function and Activity Performance in Pediatric Hand Burns: A Randomized Controlled Trial. *Archives of physical medicine and rehabilitation* 2021; **102**(6): 1059-1066.  316. KAMEL FAH, BASHA MA. Effects of Virtual Reality and Task-Oriented Training on Hand Function and Activity Performance in Pediatric Hand Burns: A Randomized Controlled Trial. *Archives of physical medicine and rehabilitation* 2021; **102**(6): 1059-1066.  317. KARGAR N, DELDAR K, AHMADABADI A, et al. Can a self-care educational mobile application improve the quality of life of victims with hand burns? A randomized controlled trial. *Crescent Journal of Medical and Biological Sciences* 2020; **7**(4): 497-502.  318. KARIMIANKAKOLAKI Z, MAHMOODABAD SSM, KAZEMI A. Designing, implementing and evaluating an educational program regarding the effects of second-hand smoke in pregnancy on the knowledge, attitude and performance of male smokers. *Reproductive Health* 2023; **20**(1): 8.  319. KAWOHL W. Employment and mental health. *European Psychiatry* 2015; **30**: 96.  320. KENDALL KL, HYDE PN, FAIRMAN CM, et al. A Randomized, Double-Blind, Placebo-Controlled Trial to Determine the Effectiveness and Safety of a Thermogenic Supplement in Addition to an Energy-Restricted Diet in Apparently Healthy Females. *J Diet Suppl* 2017; **14**(6): 653-666.  321. KENNEDY SG, LEAHY AA, SMITH JJ, et al. Process Evaluation of a School-Based High-Intensity Interval Training Program for Older Adolescents: The Burn 2 Learn Cluster Randomised Controlled Trial. *Children-Basel* 2020; **7**(12): 22.  322. KHAMEES KM, DELDAR K, YAZARLU O, et al. Effect of augmented reality–based rehabilitation of hand burns on hand function in children: A randomized controlled trial. *Journal of Hand Therapy* 2024.  323. KHANIPOUR M, LAJEVARDI L, TAGHIZADEH G, et al. The investigation of the effects of occupation-based intervention on anxiety, depression, and sleep quality of subjects with hand and upper extremity burns: A randomized clinical trial. *Burns : journal of the International Society for Burn Injuries* 2022; **48**(7): 1645-1652.  324. KHANIPOUR M, LAJEVARDI L, TAGHIZADEH G, et al. The investigation of the effects of occupation-based intervention on anxiety, depression, and sleep quality of subjects with hand and upper extremity burns: A randomized clinical trial. *Burns : journal of the International Society for Burn Injuries* 2022; **48**(7): 1645-1652.  325. KHANIPOUR M, LAJEVARDI L, TAGHIZADEH G, et al. The investigation of the effects of occupation-based intervention on anxiety, depression, and sleep quality of subjects with hand and upper extremity burns: A randomized clinical trial. *Burns : journal of the International Society for Burn Injuries* 2022; **48**(7): 1645-1652.  326. KHANIPOUR M, LAJEVARDI L, TAGHIZADEH G, et al. Effects of an Occupation-Based Intervention on Hand and Upper Extremity Function, Daily Activities, and Quality of Life in People With Burn Injuries: A Randomized Controlled Trial. *The American journal of occupational therapy : official publication of the American Occupational Therapy Association* 2023; **77**(5).  327. KHANIPOUR M, LAJEVARDI L, TAGHIZADEH G, et al. Effects of an Occupation-Based Intervention on Hand and Upper Extremity Function, Daily Activities, and Quality of Life in People With Burn Injuries: A Randomized Controlled Trial. *Am J Occup Ther* 2023; **77**(5).  328. KHANIPOUR M, LAJEVARDI L, TAGHIZADEH G, et al. Effects of an Occupation-Based Intervention on Hand and Upper Extremity Function, Daily Activities, and Quality of Life in People With Burn Injuries: A Randomized Controlled Trial. *American Journal of Occupational Therapy* 2023; **77**(5): 9.  329. KIM JB, CHO YS, JANG KU, et al. Effects of sustained release growth hormone treatment during the rehabilitation of adult severe burn survivors. *Growth hormone & IGF research : official journal of the Growth Hormone Research Society and the International IGF Research Society* 2016; **27**: 1-6.  330. KIM MH, OH AY, HAN SH, et al. The effect of magnesium sulphate on intubating condition for rapid-sequence intubation: a randomized controlled trial. *Journal of Clinical Anesthesia* 2015; **27**(7): 595-601.  331. KIMURA R, HAYASHI N, UTSUNOMIYA A. Effect of a Japanese Version of the Burns Wean Assessment Program e-Learning Materials on Ventilator Withdrawal for Intensive Care Unit Nurses. *Journal of Nursing Research* 2023; **31**(4): 9.  332. KINSELLA EA, SMITH K, BHANJI S, et al. Mindfulness in allied health and social care professional education: a scoping review. *Disability and rehabilitation* 2020; **42**(2): 283-295.  333. KNEIB CJ, CARROUGHER GJ, RHODES L, et al. Sleep after discharge: A northwest regional burn model system cohort study of burn subjects in a home-based virtual rehabilitation randomized trial. *Burns Open* 2024; **8**(3): 228-236.  334. KNICKENBERG RJ. Phase model in medical and professional rehabilitation in psychosomatic diseases. *Pravention und Rehabilitation* 2009; **21**(4): 173-182.  335. KNOBLOCH K, JOEST B, KRäMER R, et al. Cellulite and focused extracorporeal shockwave therapy for non-invasive body contouring: A randomized trial. *Dermatology and Therapy* 2013; **3**(2): 143-155.  336. KOLMUS AM, HOLLAND AE, BYRNE MJ, et al. The effects of splinting on shoulder function in adult burns. *Burns : journal of the International Society for Burn Injuries* 2012; **38**(5): 638‐644.  337. KOLMUS AM, HOLLAND AE, BYRNE MJ, et al. The effects of splinting on shoulder function in adult burns. *Burns : journal of the International Society for Burn Injuries* 2012; **38**(5): 638-644.  338. KRAMER DN, LANDOLT MA. Early psychological intervention in accidentally injured children ages 2-16: a randomized controlled trial. *European Journal of Psychotraumatology* 2014; **5**: 13.  339. KUDCHADKAR SR, BERGER J, PATEL R, et al. Non-pharmacological interventions for sleep promotion in hospitalized children. *Cochrane Database of Systematic Reviews* 2022; **2022**(6).  340. KUDCHADKAR SR, BERGER J, PATEL R, et al. Non-pharmacological interventions for sleep promotion in hospitalized children. *Cochrane Database of Systematic Reviews* 2022; (6): 72.  341. KUTENAI HJ, JAFARI H, SHAFIPOUR V, et al. Comparison of the effects of Benson relaxation technique and nature sounds on pain, anxiety, and body image in burn-injured patients admitted to the burn ICU: A single-blind randomized clinical trial. *Burns : journal of the International Society for Burn Injuries* 2023; **49**(6): 1439-1447.  342. KUTENAI HJ, JAFARI H, SHAFIPOUR V, et al. Comparison of the effects of Benson relaxation technique and nature sounds on pain, anxiety, and body image in burn-injured patients admitted to the burn ICU: A single-blind randomized clinical trial. *Burns : journal of the International Society for Burn Injuries* 2023; **49**(6): 1439-1447.  343. KUTENAI HJ, JAFARI H, SHAFIPOUR V, et al. Comparison of the effects of Benson relaxation technique and nature sounds on pain, anxiety, and body image in burn-injured patients admitted to the burn ICU: A single-blind randomized clinical trial. *Burns : journal of the International Society for Burn Injuries* 2023; **49**(6): 1439-1447.  344. LAN X, TAN Z, ZHOU T, et al. Use of Virtual Reality in Burn Rehabilitation: A Systematic Review and Meta-analysis. *Archives of physical medicine and rehabilitation* 2023; **104**(3): 502-513.  345. LAN XD, TAN ZM, ZHOU T, et al. Use of Virtual Reality in Burn Rehabilitation: A Systematic Review and Meta-analysis. *Archives of physical medicine and rehabilitation* 2023; **104**(3): 502-513.  346. LANTIS K, SCHNELL P, BLAND CR, et al. Biomechanical effect of neurologic dance training (NDT) for breast cancer survivors with chemotherapy-induced neuropathy: study protocol for a randomized controlled trial and preliminary baseline data. *Trials* 2023; **24**(1): 17.  347. LAPSHIN VP, SMIRNOV SV, LOGINOV LP, et al. [Low intensity electrical stimulation in rehabilitation of patients with inhalation trauma]. *Voprosy kurortologii, fizioterapii, i lechebnoi fizicheskoi kultury* 2001; (3): 18-19.  348. LASALLE L, RACHELSKA G, NEDELEC B. Naltrexone for the management of post-burn pruritus: A preliminary report. *Burns : journal of the International Society for Burn Injuries* 2008; **34**(6): 797-802.  349. LATIF H, BAEZ SOSA V, SEGAL KERSUN L, et al. Using Virtual Reality to Promote Mindfulness Among Medical Trainees; A Study of Feasibility and Acceptance. *Blood* 2020; **136**: 39.  350. LATIF H, SOSA VB, KERSUN LS, et al. Using virtual reality to promote mindfulness among medical trainees; a study of feasibility and acceptance. *Blood* 2020; **136**(SUPPL 1): 39.  351. LAU PW, WONG DEL P, NGO JK, et al. Effects of high-intensity intermittent running exercise in overweight children. *European journal of sport science* 2015; **15**(2): 182-190.  352. LAWRIE TA, NARDIN JM, KULIER R, et al. Techniques for the interruption of tubal patency for female sterilisation. *Cochrane Database of Systematic Reviews* 2011; (2): 56.  353. LEAHY AA, DIALLO TMO, EATHER N, et al. Mediating effects of sleep on mental health in older adolescents: Findings from the Burn 2 Learn randomized controlled trial. *Scandinavian journal of medicine & science in sports* 2023; **33**(11): 2369-2380.  354. LEAHY AA, DIALLO TMO, EATHER N, et al. Mediating effects of sleep on mental health in older adolescents: Findings from the Burn 2 Learn randomized controlled trial. *Scand J Med Sci Sports* 2023; **33**(11): 2369-2380.  355. LEAHY AA, DIALLO TMO, EATHER N, et al. Mediating effects of sleep on mental health in older adolescents: Findings from the Burn 2 Learn randomized controlled trial. *Scandinavian Journal of Medicine & Science in Sports* 2023; **33**(11): 2369-2380.  356. LEAHY AA, EATHER N, SMITH JJ, et al. School-based physical activity intervention for older adolescents: rationale and study protocol for the Burn 2 Learn cluster randomised controlled trial. *Bmj Open* 2019; **9**(5): 14.  357. LEAHY AA, EATHER N, SMITH JJ, et al. Feasibility and Preliminary Efficacy of a Teacher-Facilitated High-Intensity Interval Training Intervention for Older Adolescents. *Pediatric exercise science* 2019; **31**(1): 107-117.  358. LEAHY AA, EATHER N, SMITH JJ, et al. Feasibility and Preliminary Efficacy of a Teacher-Facilitated High-Intensity Interval Training Intervention for Older Adolescents. *Pediatr Exerc Sci* 2019; **31**(1): 107-117.  359. LEAHY AA, KENNEDY SG, SMITH JJ, et al. Feasibility of a school-based physical activity intervention for adolescents with disability. *Pilot and feasibility studies* 2021; **7**(1).  360. LEAHY AA, MICHELS MFI, EATHER N, et al. Feasibility of test administration and preliminary findings for cognitive control in the Burn 2 learn pilot randomised controlled trial. *J Sports Sci* 2020; **38**(15): 1708-1716.  361. LEAHY AA, MICHELS MFI, EATHER N, et al. Feasibility of test administration and preliminary findings for cognitive control in the Burn 2 learn pilot randomised controlled trial. *Journal of Sports Sciences* 2020; **38**(15): 1708-1716.  362. LEDOUX J, MEYER WJ, 3RD, BLAKENEY PE, et al. Relationship between parental emotional states, family environment and the behavioural adjustment of pediatric burn survivors. *Burns : journal of the International Society for Burn Injuries* 1998; **24**(5): 425-432.  363. LEE KK, PERRY AS, WOLF SA, et al. Promoting routine stair use: evaluating the impact of a stair prompt across buildings. *American journal of preventive medicine* 2012; **42**(2): 136-141.  364. LEWINSON RT, CAPOZZI LC, JOHNSON K, et al. A Review of Perforator Flaps for Burn Scar Contractures of Joints. *Plast Surg* 2019; **27**(1): 66-77.  365. LI J, ZHOU L, WANG Y. The effects of music intervention on burn patients during treatment procedures: A systematic review and meta-analysis of randomized controlled trials. *BMC complementary and alternative medicine* 2017; **17**(1).  366. LI JD, LV GZ. Letter to the Editor on "Potential efficacy of sensorimotor exercise program on pain, proprioception, mobility, and quality of life in diabetic patients with foot burns: a 12-week randomized control study". *Burns : journal of the International Society for Burn Injuries* 2021; **47**(5): 1203‐1204.  367. LI JD, LV GZ. Letter to the Editor on “Potential efficacy of sensorimotor exercise program on pain, proprioception, mobility, and quality of life in diabetic patients with foot burns: A 12-week randomized control study”. *Burns : journal of the International Society for Burn Injuries* 2021; **47**(5): 1203-1204.  368. LI JY, ZHOU L, WANG YG. The effects of music intervention on burn patients during treatment procedures: a systematic review and meta-analysis of randomized controlled trials. *BMC complementary and alternative medicine* 2017; **17**: 14.  369. LI L, DAI JX, XU L, et al. The effect of a rehabilitation nursing intervention model on improving the comprehensive health status of patients with hand burns. *Burns : journal of the International Society for Burn Injuries* 2017; **43**(4): 877‐885.  370. LI L, DAI JX, XU L, et al. The effect of a rehabilitation nursing intervention model on improving the comprehensive health status of patients with hand burns. *Burns : journal of the International Society for Burn Injuries* 2017; **43**(4): 877-885.  371. LI L, DAI JX, XU L, et al. The effect of a rehabilitation nursing intervention model on improving the comprehensive health status of patients with hand burns. *Burns : journal of the International Society for Burn Injuries* 2017; **43**(4): 877-885.  372. LIANG ZQ, LI HM, MENG CY. Repair of second degree facial burns in children using recombinant human epidermal growth factor. *Journal of Clinical Rehabilitative Tissue Engineering Research* 2007; **11**(10): 1974-1975.  373. LIN HT, LI YI, HU WP, et al. A Scoping Review of The Efficacy of Virtual Reality and Exergaming on Patients of Musculoskeletal System Disorder. *Journal of clinical medicine* 2019; **8**(6): 17.  374. LIN SL, HUANG CY, SHIU SP, et al. Effects of Yoga on Stress, Stress Adaption, and Heart Rate Variability Among Mental Health Professionals--A Randomized Controlled Trial. *Worldviews on evidence-based nursing / Sigma Theta Tau International, Honor Society of Nursing* 2015; **12**(4): 236-245.  375. LIN SL, HUANG CY, SHIU SP, et al. Effects of Yoga on Stress, Stress Adaption, and Heart Rate Variability Among Mental Health Professionals--A Randomized Controlled Trial. *Worldviews on evidence-based nursing* 2015; **12**(4): 236-245.  376. LIN SL, HUANG CY, SHIU SP, et al. Effects of Yoga on Stress, Stress Adaption, and Heart Rate Variability Among Mental Health ProfessionalsA Randomized Controlled Trial. *Worldviews on evidence-based nursing* 2015; **12**(4): 236-245.  377. LO SF, HAYTER M, HSU M, et al. The effectiveness of multimedia learning education programs on knowledge, anxiety and pressure garment compliance in patients undergoing burns rehabilitation in Taiwan: an experimental study. *Journal of clinical nursing* 2010; **19**(1-2): 129-137.  378. LOHMAN EB, 3RD, BAINS GS, LOHMAN T, et al. A comparison of the effect of a variety of thermal and vibratory modalities on skin temperature and blood flow in healthy volunteers. *Medical science monitor : international medical journal of experimental and clinical research* 2011; **17**(9): Mt72-81.  379. LUBANS D, SMITH J, EATHER N, et al. Time-efficient physical activity intervention for older adolescents: The Burn 2 Learn cluster randomised controlled trial (Jan, 10.1016/j.jsams.2021.09.018, 2022). *Journal of Science and Medicine in Sport* 2022; **25**(3): E1-E1.  380. LUBANS DR, SMITH JJ, EATHER N, et al. Time-efficient intervention to improve older adolescents' cardiorespiratory fitness: findings from the 'Burn 2 Learn' cluster randomised controlled trial. *British Journal of Sports Medicine* 2021; **55**(13): 751-+.  381. LUO X, CEN Y, YU R, et al. Effectiveness of recombinant human growth hormone treatment for severe burn injury. *Journal of West China University of Medical Sciences* 2000; **31**(3): 399-401.  382. LUO X, CEN Y, YU R, et al. [Effectiveness of recombinant human growth hormone treatment for severe burn injury]. *Hua xi yi ke da xue xue bao = Journal of West China University of Medical Sciences = Huaxi yike daxue xuebao* 2000; **31**(3): 399-401.  383. MAANI CV, DESOCIO PA, JANSEN RK, et al. Use of Ultra Rapid Opioid Detoxification in the Treatment of US Military Burn Casualties. *J Trauma-Injury Infect Crit Care* 2011; **71**: S114-S119.  384. MALAGARIS I, HERNDON DN, POLYCHRONOPOULOU E, et al. Determinants of skeletal muscle protein turnover following severe burn trauma in children. *Clin Nutr* 2019; **38**(3): 1348-1354.  385. MALIK SS, TASSADAQ N. Effectiveness of Deep Breathing Exercises and Incentive Spirometry on Arterial Blood Gases in Second Degree Inhalation Burn Patients. *Journal of the College of Physicians and Surgeons--Pakistan : JCPSP* 2019; **29**(10): 954‐957.  386. MALIK SS, TASSADAQ N. Effectiveness of Deep Breathing Exercises and Incentive Spirometry on Arterial Blood Gases in Second Degree Inhalation Burn Patients. *Jcpsp-Journal of the College of Physicians and Surgeons Pakistan* 2019; **29**(10): 954-957.  387. MARTINEZ L, ESTEVE V, YESTE M, et al. Neuromuscular Electrostimulation as a New Therapeutic Option to Improve Radio-cephalic Arteriovenous Fistula Maturation in End Stage Chronic Kidney Disease Patients. *European Journal of Vascular and Endovascular Surgery* 2019; **58**(6): e455-e457.  388. MARTYN JA, GOUDSOUZIAN NG, CHANG Y, et al. Neuromuscular effects of mivacurium in 2- to 12-yr-old children with burn injury. *Anesthesiology* 2000; **92**(1): 31-37.  389. MASIC U, CHRISTIANSEN P, BOYLAND EJ. The influence of calorie and physical activity labelling on snack and beverage choices. *Appetite* 2017; **112**: 52-58.  390. MATSUOKA H, CHIBA I, SAKANO Y, et al. Cognitive behavioral therapy for psychosomatic problems in dental settings. *BioPsychoSoc Med* 2017; **11**: 7.  391. MAVILIDI MF, MASON C, LEAHY AA, et al. Effect of a Time-Efficient Physical Activity Intervention on Senior School Students' On-Task Behaviour and Subjective Vitality: the 'Burn 2 Learn' Cluster Randomised Controlled Trial. *Educational Psychology Review* 2021; **33**(1): 299-323.  392. MAYNARD TS, BURNS CA. Balance Rehabilitation in Blinded Elders, BRIBE. *Journal of Cardiopulmonary Rehabilitation and Prevention* 2023; **43**(5): E12.  393. MEAUME S, LE PILLOUER-PROST A, RICHERT B, et al. Management of scars: updated practical guidelines and use of silicones. *Eur J Dermatol* 2014; **24**(4): 435-443.  394. MEAUME S, PEREZ J, DESCAMPS H, et al. Use of a new, flexible lipidocolloid dressing on acute and chronic wounds: Results of a clinical study. *Journal of Wound Care* 2011; **20**(4): 180-185.  395. MEAUME S, PEREZ J, DESCAMPS H, et al. Use of a new, flexible lipidocolloid dressing on acute and chronic wounds: results of a clinical study. *J Wound Care* 2011; **20**(4): 180, 182-185.  396. MEAUME S, PEREZ J, DESCAMPS H, et al. Use of a new, flexible lipidocolloid dressing on acute and chronic wounds: results of a clinical study. *Journal of Wound Care* 2011; **20**(4): 180-+.  397. MEIJER HA, GRAAFLAND M, GOSLINGS JC, et al. Systematic Review on the Effects of Serious Games and Wearable Technology Used in Rehabilitation of Patients With Traumatic Bone and Soft Tissue Injuries. *Archives of physical medicine and rehabilitation* 2018; **99**(9): 1890-1899.  398. MELCHERT-MCKEARNAN K, DEITZ J, ENGEL JM, et al. Children with burn injuries: purposeful activity versus rote exercise. *The american journal of occupational therapy : official publication of the american occupational therapy association* 2000; **54**(4): 381‐390.  399. MELCHERT-MCKEARNAN K, DEITZ J, ENGEL JM, et al. Children with burn injuries: purposeful activity versus rote exercise. *The American journal of occupational therapy : official publication of the American Occupational Therapy Association* 2000; **54**(4): 381-390.  400. MELCHERT-MCKEARNAN K, DEITZ J, ENGEL JM, et al. Children with burn injuries: purposeful activity versus rote exercise. *Am J Occup Ther* 2000; **54**(4): 381-390.  401. MELLO MJ, BAIRD J, LEE C, et al. A Randomized Controlled Trial of a Telephone Intervention for Alcohol Misuse With Injured Emergency Department Patients. *Annals of Emergency Medicine* 2016; **67**(2): 263-275.  402. MERILLAT BD, GONZáLEZ-VALLEJO C. How Much Sugar is in My Drink? The Power of Visual Cues. *Nutrients* 2020; **12**(2).  403. MICHISHITA T, KOBAYASHI S, KATSUYA T, et al. Evaluation of the Antiobesity Effects of an Amino Acid Mixture and Conjugated Linoleic Acid on Exercising Healthy Overweight Humans: a Randomized, Double-blind, Placebo-controlled Trial. *Journal of International Medical Research* 2010; **38**(3): 844-859.  404. MILLER JT, BTAICHE IF. Oxandrolone in pediatric patients with severe thermal burn injury. *Annals of Pharmacotherapy* 2008; **42**(9): 1310-1315.  405. MILLER JT, BTAICHE IF. Oxandrolone treatment in adults with severe thermal injury. *Pharmacotherapy* 2009; **29**(2): 213-226.  406. MIRI S, HOSSEINI SJ, TAKASI P, et al. Effects of breathing exercise techniques on the pain and anxiety of burn patients: A systematic review and meta-analysis. *International Wound Journal* 2023; **20**(6): 2360-2375.  407. MOIEMEN N, MATHERS J, JONES L, et al. Pressure garment to prevent abnormal scarring after burn injury in adults and children: the PEGASUS feasibility RCT and mixed-methods study. *Health technology assessment (Winchester, England)* 2018; **22**(36): 1‐162.  408. MORENO-DUARTE I, MORSE LR, ALAM M, et al. Targeted therapies using electrical and magnetic neural stimulation for the treatment of chronic pain in spinal cord injury. *Neuroimage* 2014; **85**: 1003-1013.  409. MORTADA H, ZAHRELDIN AA, SALEH MS, et al. The Efficacy of Whole-Body Vibration in Managing Postburn Victims' Complications: A Systematic Review. *Journal of Burn Care & Research* 2024; **45**(1): 48-54.  410. MUDAWARIMA T, CHIWARIDZO M, JELSMA J, et al. A systematic review protocol on the effectiveness of therapeutic exercises utilised by physiotherapists to improve function in patients with burns. *Systematic reviews* 2017; **6**: 7.  411. MURPHY KD, THOMAS S, MLCAK RP, et al. Effects of long-term oxandrolone administration in severely burned children. *Surgery* 2004; **136**(2): 219-224.  412. MURPHY KD, THOMAS S, MLCAK RP, et al. Effects of long-term oxandrolone administration in severely burned children. *Surgery* 2004; **136**(2): 219-224.  413. MURPHY KD, THOMAS S, MLCAK RP, et al. Effects of long-term oxandrolone administration in severely burned children. *Surgery* 2004; **136**(2): 219-224.  414. MUTTON DL, SCREMIN AM, BARSTOW TJ, et al. Physiologic responses during functional electrical stimulation leg cycling and hybrid exercise in spinal cord injured subjects. *Archives of physical medicine and rehabilitation* 1997; **78**(7): 712-718.  415. NAMBI G, ABDELBASSET WK. Efficacy of Maitland joint mobilization technique on pain intensity, mouth opening, functional limitation, kinesiophobia, sleep quality and quality of life in temporomandibular joint dysfunction following bilateral cervicofacial burns. *Burns : journal of the International Society for Burn Injuries* 2020; **46**(8): 1880-1888.  416. NAMBI G, ABDELBASSET WK, ELSAYED SH, et al. Role of electro acupuncture therapy on temporomandibular joint pain with orofacial myalgia following post healed unilateral cervicofacial burn. *Acupuncture and Electro-Therapeutics Research* 2021; **46**(2): 123-134.  417. NAMBI G, ABDELBASSET WK, ELSHEHAWY AA, et al. Yoga in Burn: Role of pranayama breathing exercise on pulmonary function, respiratory muscle activity and exercise tolerance in full-thickness circumferential burns of the chest. *Burns : journal of the International Society for Burn Injuries* 2021; **47**(1): 206-214.  418. NAMBI G, ABDELBASSET WK, SOLIMAN GS, et al. Clinical and functional efficacy of gallium–arsenide super pulsed laser therapy on temporo mandibular joint pain with orofacial myalgia following healed unilateral cervicofacial burn — A randomized trial. *Burns : journal of the International Society for Burn Injuries* 2022; **48**(2): 404-412.  419. NAMBI G, ABDELBASSET WK, SOLIMAN GS, et al. Clinical and functional efficacy of gallium-arsenide super pulsed laser therapy on temporo mandibular joint pain with orofacial myalgia following healed unilateral cervicofacial burn - A randomized trial. *Burns : journal of the International Society for Burn Injuries* 2022; **48**(2): 404-412.  420. NAMBI G, ABDELBASSET WK, SOLIMAN GS, et al. Clinical and functional efficacy of gallium-arsenide super pulsed laser therapy on temporo mandibular joint pain with orofacial myalgia following healed unilateral cervicofacial burn - A randomized trial. *Burns : journal of the International Society for Burn Injuries* 2022; **48**(2): 404-412.  421. NAMBI G, ALGHADIER M, EBRAHIM EE, et al. Role of virtual reality distraction technique to improve chest burns with acute respiratory distress syndrome (ARDS) following smoke inhalation in middle-aged adults - A randomized controlled study. *Burns : journal of the International Society for Burn Injuries* 2023; **49**(7): 1643‐1653.  422. NAMBI G, ALGHADIER M, EBRAHIM EE, et al. Role of virtual reality distraction technique to improve chest burns with acute respiratory distress syndrome (ARDS) following smoke inhalation in middle-aged adults - A randomized controlled study. *Burns : journal of the International Society for Burn Injuries* 2023; **49**(7): 1643‐1653.  423. NASH MS, MELTZER NM, MARTINS SC, et al. Nutrient Supplementation Post Ambulation in Persons With Incomplete Spinal Cord Injuries: A Randomized, Double-Blinded, Placebo-Controlled Case Series. *Archives of physical medicine and rehabilitation* 2007; **88**(2): 228-233.  424. NATALE VM, BRENNER IK, MOLDOVEANU AI, et al. Effects of three different types of exercise on blood leukocyte count during and following exercise. *Sao Paulo Medical Journal* 2003; **121**(1): 9-14.  425. NATALE VM, BRENNER IK, MOLDOVEANU AI, et al. Effects of three different types of exercise on blood leukocyte count during and following exercise. *Sao Paulo medical journal = Revista paulista de medicina* 2003; **121**(1): 9-14.  426. NAYLOR EV, ANTONUCCIO DO, LITT M, et al. Bibliotherapy as a Treatment for Depression in Primary Care. *Journal of Clinical Psychology in Medical Settings* 2010; **17**(3): 258-271.  427. NCT. Effects of a Community Based Exercise Program in Adults With Severe Burns. *https://clinicaltrialsgov/show/NCT01184547* 2010.  428. NCT. Amino Acid Supplementation in Recovery From Severe Burns. *https://clinicaltrialsgov/show/NCT01618630* 2012.  429. NCT. Effect of In-Patient Exercise Training on Length of Hospitalization in Burned Patients. *https://clinicaltrialsgov/show/NCT02739464* 2015.  430. NCT. A Prospective Trial of Virtual Home Rehabilitation After Burn Injury. *https://clinicaltrialsgov/show/NCT03475654* 2018.  431. NCT. The Effects of Robot-assisted Gait Training(RAGT) On Patients With Burn. *https://clinicaltrialsgov/show/NCT03992547* 2019.  432. NCT. STAT: standard Therapy Plus Active Therapy. *https://clinicaltrialsgov/ct2/show/NCT04368117* 2020.  433. NCT. Antigravity Treadmill Training on Gait Characteristics and Balance. *https://clinicaltrialsgov/show/NCT05493696* 2022.  434. NCT. The Effect of Aerobic Exercise on Neutrophil-Lymphocyte Ratio, Platelet-Lymphocyte Ratio and Lymphocyte-Monocyte Ratio in Burn Patients: a Randomized Controlled Study. *https://clinicaltrialsgov/ct2/show/NCT06013553* 2023.  435. NCT. Impact of a Telerehabilitation Program With Technology Enhancement on Post-burn Recovery. *https://clinicaltrialsgov/ct2/show/NCT06162052* 2023.  436. NCT. Skin Wetting in Burn Survivors. *https://clinicaltrialsgov/ct2/show/NCT06529757* 2024.  437. NEDELEC B, CARTER A, FORBES L, et al. Practice Guidelines for the Application of Nonsilicone or Silicone Gels and Gel Sheets After Burn Injury. *Journal of Burn Care & Research* 2015; **36**(3): 345-374.  438. NELSON MU, BIZZARRO MJ, DEMBRY LM, et al. One size does not fit all: why universal decolonization strategies to prevent methicillin-resistant <i>Staphylococcus aureus</i> colonization and infection in adult intensive care units may be inappropriate for neonatal intensive care units. *Journal of Perinatology* 2014; **34**(9): 655-657.  439. NEUBAUER H, STOLLE A, RIPPER S, et al. Evaluation of an International Classification of Functioning, Disability and Health-based rehabilitation for thermal burn injuries: A prospective non-randomized design. *Trials* 2019; **20**(1).  440. NOONAN CW, SEMMENS EO, WARE D, et al. Wood stove interventions and child respiratory infections in rural communities: KidsAir rationale and methods. *Contemporary Clinical Trials* 2020; **89**: 7.  441. NORMAN GJ, ADAMS MA, CALFAS KJ, et al. A randomized trial of a multicomponent intervention for adolescent sun protection behaviors. *Archives of pediatrics & adolescent medicine* 2007; **161**(2): 146-152.  442. NURMATOV UB, MULLEN S, QUINN-SCOGGINS H, et al. The effectiveness and cost-effectiveness of first aid interventions for burns given to caregivers of children: A systematic review. *Burns : journal of the International Society for Burn Injuries* 2018; **44**(3): 512-523.  443. ODENDAAL W, VAN NIEKERK A, JORDAAN E, et al. The impact of a home visitation programme on household hazards associated with unintentional childhood injuries: A randomised controlled trial. *Accident Analysis and Prevention* 2009; **41**(1): 183-190.  444. OEN I, VAN BAAR ME, MIDDELKOOP E, et al. Effectiveness of Cerium Nitrate-Silver Sulfadiazine in the Treatment of Facial Burns: A Multicenter, Randomized, Controlled Trial. *Plastic and reconstructive surgery* 2012; **130**(2): 274E-283E.  445. OGDEN HB, CHILD RB, FALLOWFIELD JL, et al. Gastrointestinal Tolerance of Low, Medium and High Dose Acute Oral l-Glutamine Supplementation in Healthy Adults: A Pilot Study. *Nutrients* 2020; **12**(10).  446. OGDEN HB, CHILD RB, FALLOWFIELD JL, et al. Gastrointestinal Tolerance of Low, Medium and High Dose Acute Oral l-Glutamine Supplementation in Healthy Adults: A Pilot Study. *Nutrients* 2020; **12**(10): 13.  447. OHRTMAN EA, ZANINOTTO AL, CARVALHO S, et al. Longitudinal Clinical Trial Recruitment and Retention Challenges in the Burn Population: Lessons Learned From a Trial Examining a Novel Intervention for Chronic Neuropathic Symptoms. *Journal of Burn Care & Research* 2019; **40**(6): 792-795.  448. OKHOVATIAN F, ZOUBINE N. A comparison between two burn rehabilitation protocols. *Burns : journal of the International Society for Burn Injuries* 2007; **33**(4): 429-434.  449. OKHOVATIAN F, ZOUBINE N. A comparison between two burn rehabilitation protocols. *Burns : journal of the International Society for Burn Injuries* 2007; **33**(4): 429-434.  450. OKHOVATIAN F, ZOUBINE N. A comparison between two burn rehabilitation protocols. *Burns : journal of the International Society for Burn Injuries* 2007; **33**(4): 429-434.  451. OLIVEIRA D, DE STEUR H, LAGAST S, et al. The impact of calorie and physical activity labelling on consumer's emo-sensory perceptions and food choices. *Food Res Int* 2020; **133**: 109166.  452. OLUSANYA A, YEARSLEY A, BROWN N, et al. Capsaicin 8% Patch for Spinal Cord Injury Focal Neuropathic Pain, a Randomized Controlled Trial. *Pain Med* 2023; **24**(1): 71-78.  453. OMAR MT, HEGAZY FA, MOKASHI SP. Influences of purposeful activity versus rote exercise on improving pain and hand function in pediatric burn. *Burns : journal of the International Society for Burn Injuries* 2012; **38**(2): 261-268.  454. ORELLANA M, Ýñ V, HIDALGO G, et al. Lidocaine patch in children with neuropathic pain from burn sequelae. *European Journal of Pain Supplements* 2011; **5**(1): 91.  455. ORR R, SINGH MF. The anabolic androgenic steroid oxandrolone in the treatment of wasting and catabolic disorders - Review of efficacy and safety. *Drugs* 2004; **64**(7): 725-750.  456. OTHMAN EM, TOSON RA. Response of bone mineral density and balance performance in post-burn patients with selected Qigong training: a single-blind randomized controlled trial. *Burns : journal of the International Society for Burn Injuries* 2024; **50**(2): 495‐506.  457. OTHMAN EM, TOSON RA. Response of bone mineral density and balance performance in post-burn patients with selected Qigong training: A single-blind randomized controlled trial. *Burns : journal of the International Society for Burn Injuries* 2024; **50**(2): 495-506.  458. ÖZKAL Ö, KISMET K, KONAN A, et al. Treadmill versus overground gait training in patients with lower limb burn injury: A matched control study. *Burns : journal of the International Society for Burn Injuries* 2022; **48**(1): 51-58.  459. ÖZKAL Ö, KıSMET K, KONAN A, et al. Treadmill versus overground gait training in patients with lower limb burn injury: a matched control study. *Burns : journal of the International Society for Burn Injuries* 2022; **48**(1): 51‐58.  460. ÖZKAL Ö, KıSMET K, KONAN A, et al. Treadmill versus overground gait training in patients with lower limb burn injury: A matched control study. *Burns : journal of the International Society for Burn Injuries* 2022; **48**(1): 51-58.  461. ÖZKAL Ö, KıSMET K, KONAN A, et al. Treadmill versus overground gait training in patients with lower limb burn injury: A matched control study. *Burns : journal of the International Society for Burn Injuries* 2022; **48**(1): 51-58.  462. ÖZKAL Ö, TOPUZ S, KARAHAN S, et al. Clinical predictors of pulmonary functions, respiratory/peripheral muscle strength and exercise capacity at discharge in adults with burn injury. *Disability and rehabilitation* 2021; **43**(20): 2875-2881.  463. PALACKIC A, ABAZIE S, PARRY I, et al. Comparison of Six-Minute Walk Test and Modified Bruce Treadmill Test in Paediatric Patients With Severe Burns: a Cross-Over Study. *Journal of rehabilitation medicine* 2022; **54**: jrm00305.  464. PALACKIC A, ABAZIE S, PARRY I, et al. Comparison of Six-Minute Walk Test and Modified Bruce Treadmill Test in Paediatric Patients With Severe Burns: A Cross-Over Study. *Journal of rehabilitation medicine* 2022; **54**: jrm00305.  465. PALACKIC A, ABAZIE S, PARRY I, et al. Comparison of Six-Minute Walk Test and Modified Bruce Treadmill Test in Paediatric Patients With Severe Burns: A Cross-Over Study. *J Rehabil Med* 2022; **54**: jrm00305.  466. PALACKIC A, REGO A, PARRY I, et al. Effects of Aerobic Exercise in the Intensive Care Unit on Patient-Reported Physical Function and Mental Health Outcomes in Severely Burned Children—A Multicenter Prospective Randomized Trial. *Journal of Personalized Medicine* 2023; **13**(3).  467. PALMGREN L, BROWN S, KARTIN D, et al. The use of serial casting in management of patients with burn injury. *Journal of Burn Care and Research* 2011; **32**: S164.  468. PALMIERI TL. What's New in Critical Care of the Burn-Injured Patient? *Clinics in plastic surgery* 2009; **36**(4): 607-+.  469. PANOSSIAN A, WIKMAN G. Pharmacology of <i>Schisandra chinensis</i> Bail.:: An overview of Russian research and uses in medicine. *J Ethnopharmacol* 2008; **118**(2): 183-212.  470. PARATZ JD, STOCKTON K, PLAZA A, et al. Intensive exercise after thermal injury improves physical, functional, and psychological outcomes. *The journal of trauma and acute care surgery* 2012; **73**(1): 186-194.  471. PARKER M, DELAHUNTY B, HEBERLEIN N, et al. Interactive gaming consoles reduced pain during acute minor burn rehabilitation: a randomized, pilot trial. *Burns : journal of the International Society for Burn Injuries* 2016; **42**(1): 91‐96.  472. PARKER M, DELAHUNTY B, HEBERLEIN N, et al. Interactive gaming consoles reduced pain during acute minor burn rehabilitation: A randomized, pilot trial. *Burns : journal of the International Society for Burn Injuries* 2016; **42**(1): 91-96.  473. PARKER M, DELAHUNTY B, HEBERLEIN N, et al. Interactive gaming consoles reduced pain during acute minor burn rehabilitation: A randomized, pilot trial. *Burns : journal of the International Society for Burn Injuries* 2016; **42**(1): 91-96.  474. PARKER M, DELAHUNTY B, HEBERLEIN N, et al. Interactive gaming consoles reduced pain during acute minor burn rehabilitation: A randomized, pilot trial. *Burns : journal of the International Society for Burn Injuries* 2016; **42**(1): 91-96.  475. PARRY I, PAINTING L, BAGLEY A, et al. A Pilot Prospective Randomized Control Trial Comparing Exercises Using Videogame Therapy to Standard Physical Therapy: 6 Months Follow-Up. *Journal of burn care & research* 2015; **36**(5): 534‐544.  476. PARRY I, PAINTING L, BAGLEY A, et al. A pilot prospective randomized control trial comparing exercises using videogame therapy to standard physical therapy: 6 Months follow-up. *Journal of Burn Care and Research* 2015; **36**(5): 534-544.  477. PARRY I, PAINTING L, BAGLEY A, et al. A Pilot Prospective Randomized Control Trial Comparing Exercises Using Videogame Therapy to Standard Physical Therapy: 6 Months Follow-Up. *Journal of burn care & research : official publication of the American Burn Association* 2015; **36**(5): 534-544.  478. PARRY I, PAINTING L, BAGLEY A, et al. A Pilot Prospective Randomized Control Trial Comparing Exercises Using Videogame Therapy to Standard Physical Therapy: 6 Months Follow-Up. *Journal of Burn Care & Research* 2015; **36**(5): 534-544.  479. PARRY IS, PAINTING L, BAGLEY A, et al. A prospective randomized controlled trial comparing video games to standard physical therapy: Six months follow up. *Journal of Burn Care and Research* 2014; **35**: S76.  480. PATERNOSTRO-SLUGA T, STIEGER M. Hand splints in rehabilitation. *Critical Reviews in Physical and Rehabilitation Medicine* 2004; **16**(4): 233-256.  481. PATIL PG, HAZAREY V, CHAUDHARI R, et al. A randomized control trial measuring the effectiveness of a mouth-exercising device for mucosal burning in oral submucous fibrosis. *Oral Surgery Oral Medicine Oral Pathology Oral Radiology* 2016; **122**(6): 713-718.  482. PENA R, HERNDON DN, ELLIOTT T, et al. Effects of community based exercise in children with severe burns. *Journal of Burn Care and Research* 2014; **35**: S76.  483. PEñA R, RAMIREZ LL, CRANDALL CG, et al. Effects of community-based exercise in children with severe burns: a randomized trial. *Burns : journal of the International Society for Burn Injuries* 2016; **42**(1): 41‐47.  484. PEñA R, RAMIREZ LL, CRANDALL CG, et al. Effects of community-based exercise in children with severe burns: A randomized trial. *Burns : journal of the International Society for Burn Injuries* 2016; **42**(1): 41-47.  485. PEñA R, RAMIREZ LL, CRANDALL CG, et al. Effects of community-based exercise in children with severe burns: A randomized trial. *Burns : journal of the International Society for Burn Injuries* 2016; **42**(1): 41-47.  486. PEñA R, SUMAN OE, ROSENBERG M, et al. One-Year Comparison of a Community-Based Exercise Program Versus a Day Hospital-Based Exercise Program on Quality of Life and Mental Health in Severely Burned Children. *Archives of physical medicine and rehabilitation* 2020; **101**(1s): S26-s35.  487. PENG H, LIANG PF, WANG A, et al. [Influences of different rehabilitative methods on function of hands and psychological anxiety of patients with deeply burned hands retaining denatured dermis and grafting large autologous skin]. *Zhonghua Shao Shang Za Zhi* 2017; **33**(5): 272-276.  488. PERALTA-RAMíREZ MI, ROBLES-ORTEGA H, NAVARRETE-NAVARRETE N, et al. Effectiveness of stress management therapy in two populations with high stress: chronic patients and healty people. *Salud Ment* 2009; **32**(3): 251-258.  489. PERERA A, PERERA C, KARUNANAYAKE A. Effectiveness of early stretching exercises for the quality of recovery of the upper limb in burnt patients. *Physiotherapy (United Kingdom)* 2015; **101**: eS1194.  490. PFISTER RR. Chemical injuries of the eye. *Ophthalmology* 1983; **90**(10): 1246-1253.  491. PFISTER RR. Chemical injuries of the eye. *Ophthalmology* 1983; **90**(10): 1246-1253.  492. PHAM TN, WONG JN, TERKEN T, et al. Feasibility of a Kinect®-based rehabilitation strategy after burn injury. *Burns : journal of the International Society for Burn Injuries* 2018; **44**(8): 2080‐2086.  493. PHAM TN, WONG JN, TERKEN T, et al. Feasibility of a Kinect®-based rehabilitation strategy after burn injury. *Burns : journal of the International Society for Burn Injuries* 2018; **44**(8): 2080-2086.  494. PHAM TN, WONG JN, TERKEN T, et al. Feasibility of a Kinece®-based rehabilitation strategy after burn injury. *Burns : journal of the International Society for Burn Injuries* 2018; **44**(8): 2080-2086.  495. PHILLIPS C, ESSICK G, ZUNIGA J, et al. Qualitative descriptors used by patients following orthognathic surgery to portray altered sensation. *J Oral Maxillofac Surg* 2006; **64**(12): 1751-1760.  496. PHIPPS A, SHARMA V, BROWN S. Pressure garment to prevent abnormal scarring after burn injury in adults and children: the PEGASUS feasibility RCT and mixed-methods study. *Health Technology Assessment* 2018; **22**(36): 1-+.  497. PLAZA A, PARATZ J, COTTRELL M. A six-week physical therapy exercise program delivered via home-based telerehabilitation is comparable to in-person programs for patients with burn injuries: a randomized, controlled, non-inferiority clinical pilot trial. *Burns : journal of the International Society for Burn Injuries* 2023; **49**(1): 55‐67.  498. PLAZA A, PARATZ J, COTTRELL M. A six-week physical therapy exercise program delivered via home-based telerehabilitation is comparable to in-person programs for patients with burn injuries: A randomized, controlled, non-inferiority clinical pilot trial. *Burns : journal of the International Society for Burn Injuries* 2023; **49**(1): 55-67.  499. PLAZA A, PARATZ J, COTTRELL M. A six-week physical therapy exercise program delivered via home-based telerehabilitation is comparable to in-person programs for patients with burn injuries: A randomized, controlled, non-inferiority clinical pilot trial. *Burns : journal of the International Society for Burn Injuries* 2023; **49**(1): 55-67.  500. PLAZA A, PARATZ J, COTTRELL M. A six-week physical therapy exercise program delivered via home-based telerehabilitation is comparable to in-person programs for patients with burn injuries: A randomized, controlled, non- inferiority clinical pilot trial. *Burns : journal of the International Society for Burn Injuries* 2023; **49**(1): 55-67.  501. PLAZA A, PARATZ J, STOCKTON K, et al. Exercise programmes are effective and safe in a burns population: A controlled trial. *Journal of Burn Care and Research* 2011; **32**: S117.  502. POLYCHRONOPOULOU E, HERNDON DN, PORTER C. The Long-Term Impact of Severe Burn Trauma on Musculoskeletal Health. *Journal of Burn Care & Research* 2018; **39**(6): 869-880.  503. POPA SL, CHIARIONI G, DAVID L, et al. The Efficacy of Hypnotherapy in the Treatment of Functional Dyspepsia. *Am J Ther* 2019; **26**(6): E704-E713.  504. POPE L, HARVEY-BERINO J. Burn and earn: A randomized controlled trial incentivizing exercise during fall semester for college first-year students. *Preventive Medicine* 2013; **56**(3-4): 197-201.  505. POPE L, HARVEY-BERINO J. Burn and earn: a randomized controlled trial incentivizing exercise during fall semester for college first-year students. *Prev Med* 2013; **56**(3-4): 197-201.  506. POPE L, HARVEY-BERINO J. Burn and earn: A randomized controlled trial incentivizing exercise during fall semester for college first-year students. *Preventive Medicine* 2013; **56**(3-4): 197-201.  507. PORRO LJ, AL-MOUSAWI AM, WILLIAMS F, et al. Effects of propranolol and exercise training in children with severe burns. *Journal of pediatrics* 2013; **162**(4): 799‐803.e791.  508. PORRO LJ, AL-MOUSAWI AM, WILLIAMS F, et al. Effects of propranolol and exercise training in children with severe burns. *Journal of Pediatrics* 2013; **162**(4): 799-803.e791.  509. PORRO LJ, AL-MOUSAWI AM, WILLIAMS F, et al. Effects of propranolol and exercise training in children with severe burns. *The Journal of pediatrics* 2013; **162**(4): 799-803.e791.  510. PORRO LJ, AL-MOUSAWI AM, WILLIAMS F, et al. Effects of Propranolol and Exercise Training in Children with Severe Burns. *Journal of Pediatrics* 2013; **162**(4): 799-+.  511. PORRO LJ, HERNDON DN, RODRIGUEZ NA, et al. Five-year outcomes after oxandrolone administration in severely burned children: a randomized clinical trial of safety and efficacy. *Journal of the American College of Surgeons* 2012; **214**(4): 489‐502; discussion 502‐484.  512. PORRO LJ, HERNDON DN, RODRIGUEZ NA, et al. Five-year outcomes after oxandrolone administration in severely burned children: A randomized clinical trial of safety and efficacy. *Journal of the American College of Surgeons* 2012; **214**(4): 489-502.  513. PORRO LJ, HERNDON DN, RODRIGUEZ NA, et al. Five-year outcomes after oxandrolone administration in severely burned children: a randomized clinical trial of safety and efficacy. *J Am Coll Surg* 2012; **214**(4): 489-502; discussion 502-484.  514. PORRO LJ, HERNDON DN, RODRIGUEZ NA, et al. Five-Year Outcomes after Oxandrolone Administration in Severely Burned Children: A Randomized Clinical Trial of Safety and Efficacy. *Journal of the American College of Surgeons* 2012; **214**(4): 489-502.  515. PRELACK K, DWYER J, SHERIDAN R, et al. Body water in children during recovery from severe burn injury using a combined tracer dilution method. *The Journal of burn care & rehabilitation* 2005; **26**(1): 67-74.  516. PRZKORA R, HERNDON DN, SUMAN OE. The effects of oxandrolone and exercise on muscle mass and function in children with severe burns. *Pediatrics* 2007; **119**(1): e109-116.  517. RADWAN NL, IBRAHIM MM, MAHMOUD WS. Effect of Wii-habilitation on spatiotemporal parameters and upper limb function post-burn in children. *Burns : journal of the International Society for Burn Injuries* 2021; **47**(4): 828‐837.  518. RAETZ J, WILSON M, COLLINS K. Varicose Veins: Diagnosis and Treatment. *Am Fam Physician* 2019; **99**(11): 682-688.  519. RAHMAN J, SCRAGG R. Factors associated with self-reported sun exposure in a multi-ethnic community sample from New Zealand. *Journal of Steroid Biochemistry and Molecular Biology* 2022; **221**: 6.  520. RAY JJ, ALVAREZ AD, ULBRICH SL, et al. Shake It Off: A Randomized Pilot Study of the Effect of Whole Body Vibration on Pain in Healing Burn Wounds. *Journal of Burn Care and Research* 2017; **38**(4): e756-e764.  521. RAY JJ, ALVAREZ AD, ULBRICH SL, et al. Shake it off: A randomized study of the effect of whole body vibration on pain in healing burn wounds. *Journal of Burn Care and Research* 2016; **37**: S121.  522. RAY JJ, ALVAREZ AD, ULBRICH SL, et al. Shake It Off: A Randomized Pilot Study of the Effect of Whole Body Vibration on Pain in Healing Burn Wounds. *Journal of burn care & research : official publication of the American Burn Association* 2017; **38**(4): e756-e764.  523. REEVES P, HERNDON D, TANKSLEY J, et al. Five-year outcomes after long-term oxandrolone administration in severely burned children: A randomized clinical trial of safety and efficacy. *Shock* 2015; **43**(6): 22.  524. REGO A, PARRY I, SEN S, et al. Exercise in the ICU and its Effects on Physical and Mental Health in Pediatric Burn. *Journal of Burn Care and Research* 2023; **44**: S72.  525. REZAEI M, JALALI R, HEYDARIKHAYAT N, et al. Effect of Telenursing and Face-to-Face Training Techniques on Quality of Life in Burn Patients: A Clinical Trial. *Archives of physical medicine and rehabilitation* 2020; **101**(4): 667-673.  526. REZAEI M, JALALI R, HEYDARIKHAYAT N, et al. Effect of Telenursing and Face-to-Face Training Techniques on Quality of Life in Burn Patients: A Clinical Trial. *Archives of physical medicine and rehabilitation* 2020; **101**(4): 667-673.  527. REZAEI M, JALALI R, HEYDARIKHAYAT N, et al. Effect of Telenursing and Face-to-Face Training Techniques on Quality of Life in Burn Patients: A Clinical Trial. *Archives of physical medicine and rehabilitation* 2020; **101**(4): 667-673.  528. RHODES L, SIBBETT S, ORTON C, et al. Sleep After Discharge: A Cohort Study of Burn Participants in a Home-Based Virtual Rehabilitation Trial. *Journal of Burn Care and Research* 2023; **44**: S65.  529. RICHARD R, SANTOS-LOZADA AR. Burn Patient Acuity Demographics, Scar Contractures, and Rehabilitation Treatment Time Related to Patient Outcomes: The ACT Study. *Journal of burn care & research : official publication of the American Burn Association* 2017; **38**(4): 230-242.  530. RICHARD RL, LESTER M, DEWEY WS, et al. Reliability of a video analysis software system to assess dorsal hand skin movement. *Journal of Burn Care and Research* 2013; **34**(2): S185.  531. RICHARD RL, MILLER SF, FINLEY RK, et al. Comparison of the effect of passive exercise v static wrapping on finger range of motion in the burned hand. *Journal of burn care & rehabilitation* 1987; **8**(6): 576‐578.  532. RICHARD RL, MILLER SF, FINLEY RK, et al. Comparison of the effect of passive exercise v static wrapping on finger range of motion in the burned hand. *Journal of burn care & rehabilitation* 1987; **8**(6): 576‐578.  533. RICHARD RL, MILLER SF, FINLEY RK, et al. Comparison of the effect of passive exercise vs. static wrapping on finger range of motion in the burned hand. *Journal of Burn Care and Rehabilitation* 1987; **8**(6): 576-578.  534. RICHARD RL, MILLER SF, FINLEY RK, JR., et al. Comparison of the effect of passive exercise v static wrapping on finger range of motion in the burned hand. *The Journal of burn care & rehabilitation* 1987; **8**(6): 576-578.  535. RIVAS E, HERNDON DN, PORTER C, et al. Short-term metformin and exercise training effects on strength, aerobic capacity, glycemic control, and mitochondrial function in children with burn injury. *American journal of physiology Endocrinology and metabolism* 2018; **314**(3): E232‐E240.  536. RIVAS E, HERNDON DN, PORTER C, et al. Short-term metformin and exercise training effects on strength, aerobic capacity, glycemic control, and mitochondrial function in children with burn injury. *Am J Physiol Endocrinol Metab* 2018; **314**(3): E232-e240.  537. RIVAS E, MCENTIRE SJ, HERNDON DN, et al. Resting β-Adrenergic Blockade Does Not Alter Exercise Thermoregulation in Children With Burn Injury: a Randomized Control Trial. *Journal of burn care & research* 2018; **39**(3): 402‐412.  538. RIVAS E, MCENTIRE SJ, HERNDON DN, et al. Resting β-Adrenergic Blockade Does Not Alter Exercise Thermoregulation in Children with Burn Injury: A Randomized Control Trial. *Journal of Burn Care and Research* 2018; **39**(3): 402-412.  539. RIVAS E, MCENTIRE SJ, HERNDON DN, et al. Resting β-Adrenergic Blockade Does Not Alter Exercise Thermoregulation in Children With Burn Injury: A Randomized Control Trial. *Journal of burn care & research : official publication of the American Burn Association* 2018; **39**(3): 402-412.  540. RIVAS E, MCENTIRE SJ, HERNDON DN, et al. Resting β-Adrenergic Blockade Does Not Alter Exercise Thermoregulation in Children With Burn Injury: A Randomized Control Trial. *Journal of Burn Care & Research* 2018; **39**(3): 402-412.  541. ROBERTSON JM, DIAS RD, GUPTA A, et al. Medical Event Management for Future Deep Space Exploration Missions to Mars. *The Journal of surgical research* 2020; **246**: 305-314.  542. RODRIGUEZ NA, DIAZ E, HUANG T, et al. Effects of early exercise on the number of joint release interventions in children with severe burns. *Journal of Burn Care and Research* 2012; **33**(2): S73.  543. ROH YS, CHO H, OH JO, et al. Effects of skin rehabilitation massage therapy on pruritus, skin status, and depression in burn survivors. *Taehan Kanho Hakhoe chi* 2007; **37**(2): 221-226.  544. ROH YS, SEO CH, JANG KU. Effects of a skin rehabilitation nursing program on skin status, depression, and burn-specific health in burn survivors. *Rehabilitation nursing : the official journal of the Association of Rehabilitation Nurses* 2010; **35**(2): 65-69.  545. RONTOYANNI VG, MALAGARIS I, HERNDON DN, et al. Skeletal Muscle Mitochondrial Function is Determined by Burn Severity, Sex, and Sepsis, and is Associated With Glucose Metabolism and Functional Capacity in Burned Children. *Shock (Augusta, Ga)* 2018; **50**(2): 141‐148.  546. RONTOYANNI VG, MALAGARIS I, HERNDON DN, et al. Skeletal muscle mitochondrial function is determined by burn severity, sex, and sepsis, and is associated with glucose metabolism and functional capacity in burned children. *Shock* 2018; **50**(2): 141-148.  547. RONTOYANNI VG, MALAGARIS I, HERNDON DN, et al. Skeletal Muscle Mitochondrial Function is Determined by Burn Severity, Sex, and Sepsis, and is Associated With Glucose Metabolism and Functional Capacity in Burned Children. *Shock* 2018; **50**(2): 141-148.  548. RONTOYANNI VG, MALAGARIS I, HERNDON DN, et al. SKELETAL MUSCLE MITOCHONDRIAL FUNCTION IS DETERMINED BY BURN SEVERITY, SEX, AND SEPSIS, AND IS ASSOCIATED WITH GLUCOSE METABOLISM AND FUNCTIONAL CAPACITY IN BURNED CHILDREN. *Shock* 2018; **50**(2): 141-148.  549. ROSE KJ, RAYMOND J, REFSHAUGE K, et al. Serial night casting increases ankle dorsiflexion range in children and young adults with Charcot-Marie-Tooth disease: a randomised trial. *Journal of Physiotherapy* 2010; **56**(2): 113-119.  550. ROSENBLOOM AL. Mecasermin (recombinant human insulin-like growth factor I). *Adv Ther* 2009; **26**(1): 40-54.  551. ROUSSEAU AF, FOIDART-DESALLE M, LEDOUX D, et al. Effects of cholecalciferol supplementation and optimized calcium intakes on vitamin D status, muscle strength and bone health: A one-year pilot randomized controlled trial in adults with severe burns. *Burns : journal of the International Society for Burn Injuries* 2015; **41**(2): 317-325.  552. ROUSSEAU AF, FOIDART-DESALLE M, LEDOUX D, et al. Effects of cholecalciferol supplementation and optimized calcium intakes on vitamin D status, muscle strength and bone health: a one-year pilot randomized controlled trial in adults with severe burns. *Burns : journal of the International Society for Burn Injuries* 2015; **41**(2): 317-325.  553. ROUSSEAU AF, FOIDART-DESALLE M, LEDOUX D, et al. Effects of cholecalciferol supplementation and optimized calcium intakes on vitamin D status, muscle strength and bone health: A one-year pilot randomized controlled trial in adults with severe burns. *Burns : journal of the International Society for Burn Injuries* 2015; **41**(2): 317-325.  554. ROUSSEAU AF, LOSSER MR, ICHAI C, et al. ESPEN endorsed recommendations: Nutritional therapy in major burns. *Clin Nutr* 2013; **32**(4): 497-502.  555. ROUSSEAU AF, PANTET O, HEYLAND DK. Nutrition after severe burn injury. *Current Opinion in Clinical Nutrition and Metabolic Care* 2023; **26**(2): 99-104.  556. ROUZFARAKH M, DELDAR K, FROUTAN R, et al. The effect of rehabilitation education through social media on the quality of life in burn patients: a randomized, controlled, clinical trial. *BMC medical informatics and decision making* 2021; **21**(1): 70.  557. ROUZFARAKH M, DELDAR K, FROUTAN R, et al. The effect of rehabilitation education through social media on the quality of life in burn patients: a randomized, controlled, clinical trial. *BMC Med Inform Decis Mak* 2021; **21**(1): 70.  558. ROUZFARAKH M, DELDAR K, FROUTAN R, et al. The effect of rehabilitation education through social media on the quality of life in burn patients: a randomized, controlled, clinical trial. *Bmc Medical Informatics and Decision Making* 2021; **21**(1): 11.  559. ROWE G, EDGAR DW, OSBORNE T, et al. Does exercise influence burn-induced inflammation: a cross-over randomised controlled feasibility trial. *PloS one* 2022; **17**(4): e0266400.  560. ROWE G, EDGAR DW, OSBORNE T, et al. Does exercise influence burn-induced inflammation: A cross-over randomised controlled feasibility trial. *PLoS ONE* 2022; **17**(4 April).  561. ROWE G, EDGAR DW, OSBORNE T, et al. Does exercise influence burn-induced inflammation: A cross-over randomised controlled feasibility trial. *PLoS One* 2022; **17**(4): e0266400.  562. ROWE G, EDGAR DW, OSBORNE T, et al. Does exercise influence burn-induced inflammation: A cross-over randomised controlled feasibility trial. *Plos One* 2022; **17**(4): 11.  563. ROY S, PAI KM, SHARMA K, et al. A randomized control trial measuring the effectiveness of a mouth-exercising device for mucosal burning in oral submucous fibrosis: a commentary. *Oral Surgery Oral Medicine Oral Pathology Oral Radiology* 2017; **123**(6): 735-735.  564. RUMBACH AF, WARD EC, CORNWELL PL, et al. Physiological Characteristics of Dysphagia Following Thermal Burn Injury. *Dysphagia* 2012; **27**(3): 370-383.  565. RUTHERFORD C, PATEL MI, TAIT MA, et al. Patient-reported outcomes in non-muscle invasive bladder cancer: a mixed-methods systematic review. *Quality of Life Research* 2021; **30**(2): 345-366.  566. SALAM RA, ARSHAD A, DAS JK, et al. Interventions to Prevent Unintentional Injuries Among Adolescents: A Systematic Review and Meta-Analysis. *J Adolesc Health* 2016; **59**(4): S76-S87.  567. SALAZAR MJB, MINKOFF H, BAYYA J, et al. Influence of Surgeon Behavior on Trainee Willingness to Speak Up: A Randomized Controlled Trial. *Journal of the American College of Surgeons* 2014; **219**(5): 1001-1007.  568. SALLAM HS, ALI A, SUMAN O, et al. Adipose tissue dysfunction in burn: Contribution to ectopic fat deposition. *Endocrine Reviews* 2015; **36**.  569. SAMHAN AF, ABDELHALIM NM, ELNAGGAR RK. Effects of interactive robot-enhanced hand rehabilitation in treatment of paediatric hand-burns: a randomized, controlled trial with 3-months follow-up. *Burns : journal of the International Society for Burn Injuries* 2020; **46**(6): 1347‐1355.  570. SAMHAN AF, ABDELHALIM NM, ELNAGGAR RK. Effects of interactive robot-enhanced hand rehabilitation in treatment of paediatric hand-burns: A randomized, controlled trial with 3-months follow-up. *Burns : journal of the International Society for Burn Injuries* 2020; **46**(6): 1347-1355.  571. SAMHAN AF, ABDELHALIM NM, ELNAGGAR RK. Effects of interactive robot-enhanced hand rehabilitation in treatment of paediatric hand-burns: A randomized, controlled trial with 3-months follow-up. *Burns : journal of the International Society for Burn Injuries* 2020; **46**(6): 1347-1355.  572. SAMHAN AF, ABDELHALIM NM, ELNAGGAR RK. Effects of interactive robot-enhanced hand rehabilitation in treatment of paediatric hand-burns: A randomized, controlled trial with 3-months follow-up. *Burns : journal of the International Society for Burn Injuries* 2020; **46**(6): 1347-1355.  573. SáNCHEZ-GERVACIO BM, LEGORRETA-SOBERANIS J, BEDOLLA-SOLANO R, et al. Impact of a Non-Formal Environmental Education Program on safe handling of pesticides among Mexican subsistence farmers: a participatory pilot study. *Human and Ecological Risk Assessment* 2021; **27**(6): 1636-1654.  574. SANTOS L, PINHEIRO MD, RIJO D. The Effects of the Compassionate Mind Training for Caregivers on Professional Quality of Life and Mental Health: Outcomes from a Cluster Randomized Trial in Residential Youth Care Settings. *Child & Youth Care Forum* 2024; **53**(1): 195-215.  575. SARUBIN J. [Increased requirement of alcuronium in burned patients]. *Anaesthesist* 1982; **31**(8): 392-395.  576. SCAPIN S, ECHEVARRíA-GUANILO ME, BOEIRA FUCULO JUNIOR PR, et al. Virtual Reality in the treatment of burn patients: A systematic review. *Burns : journal of the International Society for Burn Injuries* 2018; **44**(6): 1403-1416.  577. SCAPIN S, ECHEVARRíA-GUANILO ME, FUCULO PRB, et al. Virtual Reality in the treatment of burn patients: A systematic review. *Burns : journal of the International Society for Burn Injuries* 2018; **44**(6): 1403-1416.  578. SCHEEPERS RA, EMKE H, EPSTEIN RM, et al. The impact of mindfulness-based interventions on doctors' well-being and performance: A systematic review. *Medical Education* 2020; **54**(2): 138-149.  579. SCHIEFFELERS DR, RU T, DAI H, et al. Effects of early exercise training following severe burn injury: a randomized controlled trial. *Burns and Trauma* 2024; **12**.  580. SCHIEFFELERS DR, RU TF, DAI HA, et al. Effects of early exercise training following severe burn injury: a randomized controlled trial. *Burns & trauma* 2024; **12**: 15.  581. SCHMITT YS, HOFFMAN HG, BLOUGH DK, et al. A randomized, controlled trial of immersive virtual reality analgesia, during physical therapy for pediatric burns. *Burns : journal of the International Society for Burn Injuries* 2011; **37**(1): 61-68.  582. SCHMITT YS, HOFFMAN HG, BLOUGH DK, et al. A randomized, controlled trial of immersive virtual reality analgesia, during physical therapy for pediatric burns. *Burns : journal of the International Society for Burn Injuries* 2011; **37**(1): 61-68.  583. SCHMITT YS, HOFFMAN HG, BLOUGH DK, et al. A randomized, controlled trial of immersive virtual reality analgesia, during physical therapy for pediatric burns. *Burns : journal of the International Society for Burn Injuries* 2011; **37**(1): 61-68.  584. SCHOUTEN HJ, NIEUWENHUIS MK, VAN ZUIJLEN PPM. A review on static splinting therapy to prevent burn scar contracture: Do clinical and experimental data warrant its clinical application? *Burns : journal of the International Society for Burn Injuries* 2012; **38**(1): 19-25.  585. SCHULZ A, DEMIR E, FUCHS P, et al. Early enzymatic debridement of deep dermal burns-first results in hands and preliminary results in face and lower extremity. *Journal of Burn Care and Research* 2015; **36**: S264.  586. SCHULZ A, PERBIX W, SHOHAM Y, et al. Our initial learning curve in the enzymatic debridement of severely burned hands—Management and pit falls of initial treatments and our development of a post debridement wound treatment algorithm. *Burns : journal of the International Society for Burn Injuries* 2017; **43**(2): 326-336.  587. SCHULZ A, PERBIX W, SHOHAM Y, et al. Our initial learning curve in the enzymatic debridement of severely burned hands-Management and pit falls of initial treatments and our development of a post debridement wound treatment algorithm. *Burns : journal of the International Society for Burn Injuries* 2017; **43**(2): 326-336.  588. SCHULZ A, PERBIX W, SHOHAM Y, et al. Our initial learning curve in the enzymatic debridement of severely burned hands Management and pit falls of initial treatments and our development of a post debridement wound treatment algorithm. *Burns : journal of the International Society for Burn Injuries* 2017; **43**(2): 326-336.  589. SCHWEBEL DC, SWART D, SIMPSON J, et al. An intervention to reduce kerosene-related burns and poisonings in low-income South African communities. *Health psychology : official journal of the Division of Health Psychology, American Psychological Association* 2009; **28**(4): 493-500.  590. SCOTT HC, STOCKDALE C, ROBINSON A, et al. Is massage an effective intervention in the management of post-operative scarring? A scoping review. *Journal of Hand Therapy* 2022; **35**(2): 186-199.  591. SELLES RW, SCHREUDERS TAR, STAM HJ. Mirror therapy in patients with causalgia (complex regional pain syndrome type II) following peripheral nerve injury: Two cases. *Journal of Rehabilitation Medicine* 2008; **40**(4): 312-314.  592. SENAYLI YA, KESKIN G, AKIN M, et al. A prospective study for an alternative probe site for pulse oximetry measurement in male patients with severe burn trauma: penile shaft. *Turkish Journal of Medical Sciences* 2023; **53**(2): 504-510.  593. SERAIA EV, SMIRNOV SV, LAPSHIN VP. [Exercise therapy in combination with transcutaneous electroneurostimulation in patients with inhalation burns during intensive care]. *Voprosy kurortologii, fizioterapii, i lechebnoi fizicheskoi kultury* 2004; (1): 38.  594. SERETIS K, BOUNAS N. Securing skin grafts: A network meta-analysis. *Journal of Plastic Reconstructive and Aesthetic Surgery* 2024; **96**: 146-157.  595. SEYEDOSHOHADAEE M, GHEZELJEH TN, SAMIMI R, et al. Implementation of a nursing rehabilitation model to improve quality of life of patients with hand burns: A randomized clinical trial. *European Journal of Translational Myology* 2022; **32**(4).  596. SEYMOUR NE, GALLAGHER AG, ROMAN SA, et al. Virtual reality training improves operating room performance: results of a randomized, double-blinded study. *Annals of surgery* 2002; **236**(4): 458-463; discussion 463-454.  597. SEYMOUR NE, GALLAGHER AG, ROMAN SA, et al. Virtual reality training improves operating room performance results of a randomized, double-blinded study. *Annals of surgery* 2002; **236**(4): 458-464.  598. SHAH M, BOUZA B, ADAMS-HUET B, et al. Effect of calorie or exercise labels on menus on calories and macronutrients ordered and calories from specific foods in Hispanic participants: A randomized study. *Journal of Investigative Medicine* 2016; **64**(8): 1261-1268.  599. SHAH M, BOUZA B, ADAMS-HUET B, et al. Effect of calorie or exercise labels on menus on calories and macronutrients ordered and calories from specific foods in Hispanic participants: a randomized study. *Journal of investigative medicine : the official publication of the American Federation for Clinical Research* 2016; **64**(8): 1261-1268.  600. SHARAR SR, CARROUGHER GJ, NAKAMURA D, et al. Factors Influencing the Efficacy of Virtual Reality Distraction Analgesia During Postburn Physical Therapy: Preliminary Results from 3 Ongoing Studies. *Archives of physical medicine and rehabilitation* 2007; **88**(12 SUPPL. 2): S43-S49.  601. SHARAR SR, CARROUGHER GJ, NAKAMURA D, et al. Factors influencing the efficacy of virtual reality distraction analgesia during postburn physical therapy: preliminary results from 3 ongoing studies. *Archives of physical medicine and rehabilitation* 2007; **88**(12 Suppl 2): S43-49.  602. SHARAR SR, CARROUGHER GJ, NAKAMURA D, et al. Factors influencing the efficacy of virtual reality distraction analgesia during postburn physical therapy: Preliminary results from 3 ongoing studies. *Archives of physical medicine and rehabilitation* 2007; **88**(12): S43-S49.  603. SHAW M, PALMERI M, LADENSACK D, et al. Proceedings #9: Immersive Virtual Reality Rehabilitation for Patients with Multiple Sclerosis. *Brain Stimulation* 2019; **12**(2): e68-e69.  604. SHI JJ, SHEN AM, SUN Y, et al. Occupational activity disorders of extremely severe mass burn patients in recovery period after injury: a cross-sectional survey. *Zhonghua shao shang za zhi = Zhonghua shaoshang zazhi = Chinese journal of burns* 2018; **34**(9): 624-628.  605. SHI JJ, SUN Y, PAN SS, et al. Manufacture and clinical application of the forearm pronation's assistant tableware in the severely burned. *Burns : journal of the International Society for Burn Injuries* 2021; **47**(3): 684-691.  606. SHI JJ, SUN Y, PAN SS, et al. Manufacture and clinical application of the forearm pronation's assistant tableware in the severely burned. *Burns : journal of the International Society for Burn Injuries* 2021; **47**(3): 684-691.  607. SHIMODOZONO M, KAWAHIRA K, OGATA A, et al. Addition of an Anabolic Steroid to Strength Training Promotes Muscle Strength in the Nonparetic Lower Limb of Poststroke Hemiplegia Patients. *International Journal of Neuroscience* 2010; **120**(9): 617-624.  608. SIBBETT S, ORTON C, CARROUGHER G, et al. Home-Based Virtual Rehabilitation Does Not Improve Daily Walking in the First Twelve Weeks After Discharge. *Journal of Burn Care and Research* 2023; **44**: S12.  609. SIBBETT SH, CARROUGHER GJ, ORTON CM, et al. A Randomized Controlled Trial of Home-Based Virtual Rehabilitation to Improve Adherence to Prescribed Home Therapy after Burn Injury: A Northwest Regional Burn Model System Trial. *Journal of burn care & research : official publication of the American Burn Association* 2024.  610. SIMPSON RC, THOMAS KS, LEIGHTON P, et al. Diagnostic criteria for erosive lichen planus affecting the vulva: an international electronic-Delphi consensus exercise. *Br J Dermatol* 2013; **169**(2): 337-343.  611. SKVARC NK, KAMENIK M. Effects of pregabalin on acute herpetic pain and postherpetic neuralgia incidence. *Wien Klin Wochen* 2010; **122**: 49-53.  612. SOLTANI M, DREVER SA, HOFFMAN HG, et al. Virtual reality analgesia for burn joint flexibility: a randomized controlled trial. *Rehabilitation psychology* 2018; **63**(4): 487‐494.  613. SOLTANI M, DREYER SA, HOFFMAN HG, et al. Virtual Reality Analgesia for Burn Joint Flexibility: A Randomized Controlled Trial. *Rehabilitation Psychology* 2018; **63**(4): 487-494.  614. SOUSSE LE, HERNDON DN, MLCAK RP, et al. Long-Term Administration of Oxandrolone Improves Lung Function in Pediatric Burned Patients. *Journal of burn care & research* 2016; **37**(5): 273‐277.  615. SOUSSE LE, HERNDON DN, MLCAK RP, et al. Long-Term Administration of Oxandrolone Improves Lung Function in Pediatric Burned Patients. *Journal of Burn Care and Research* 2016; **37**(5): 273-277.  616. SOUSSE LE, HERNDON DN, MLCAK RP, et al. Long-Term Administration of Oxandrolone Improves Lung Function in Pediatric Burned Patients. *Journal of burn care & research : official publication of the American Burn Association* 2016; **37**(5): 273-277.  617. SOUSSE LE, HERNDON DN, MLCAK RP, et al. Long-Term Administration of Oxandrolone Improves Lung Function in Pediatric Burned Patients. *Journal of Burn Care & Research* 2016; **37**(5): 273-277.  618. STAIANO AE, FLYNN R. Therapeutic Uses of Active Videogames: A Systematic Review. *Games for Health Journal* 2014; **3**(6): 351-+.  619. STANGHERLIN DAC, LEMOS ID, BELLO JZ, et al. Transcutaneous Electrical Nerve Stimulation in Dysphonic Patients: A Systematic Review. *J Voice* 2021; **35**(6): 876-885.  620. STANOJCIC M, FINNERTY CC, JESCHKE MG. Anabolic and anticatabolic agents in critical care. *Curr Opin Crit Care* 2016; **22**(4): 325-331.  621. STANTON E, WON P, MANASYAN A, et al. Neuropathic pain in burn patients – A common problem with little literature: A systematic review. *Burns : journal of the International Society for Burn Injuries* 2024; **50**(5): 1053-1061.  622. STANTON E, WON P, MANASYAN A, et al. Neuropathic Pain in Burn Patients - A Common Problem with Little Literature: A Systematic Review. *Journal of Burn Care and Research* 2024; **45**: S201.  623. STOCKDALE CK, LAWSON HW. 2013 Vulvodynia Guideline Update. *Journal of Lower Genital Tract Disease* 2014; **18**(2): 93-100.  624. STOOKEY JD, KLEIN A. Short-term effects of caloric beverages and Drinking water on macronutrient metabolism. *The FASEB Journal* 2009; **23**(S1).  625. STRECKMANN F, ELTER T, LEHMANN HC, et al. Preventive Effect of Neuromuscular Training on Chemotherapy-Induced Neuropathy: A Randomized Clinical Trial. *Jama Internal Medicine* 2024: 8.  626. SUMAN OE, HERNDON DN. Effects of cessation of a structured and supervised exercise conditioning program on lean mass and muscle strength in severely burned children. *Archives of physical medicine and rehabilitation* 2007; **88**(12 Suppl 2): S24‐29.  627. SUMAN OE, HERNDON DN. Effects of Cessation of a Structured and Supervised Exercise Conditioning Program on Lean Mass and Muscle Strength in Severely Burned Children. *Archives of physical medicine and rehabilitation* 2007; **88**(12 SUPPL. 2): S24-S29.  628. SUMAN OE, HERNDON DN. Effects of cessation of a structured and supervised exercise conditioning program on lean mass and muscle strength in severely burned children. *Archives of physical medicine and rehabilitation* 2007; **88**(12 Suppl 2): S24-29.  629. SUMAN OE, MLCAK RP, HERNDON DN. Effect of exercise training on pulmonary function in children with thermal injury. *Journal of burn care & rehabilitation* 2002; **23**(4): 288‐293; discussion 287.  630. SUMAN OE, MLCAK RP, HERNDON DN. Effect of exercise training on pulmonary function in children with thermal injury. *The Journal of burn care & rehabilitation* 2002; **23**(4): 288-293; discussion 287.  631. SUMAN OE, SPIES RJ, CELIS MM, et al. Effects of a 12-wk resistance exercise program on skeletal muscle strength in children with burn injuries. *Journal of applied physiology (Bethesda, Md : 1985)* 2001; **91**(3): 1168‐1175.  632. SUMAN OE, SPIES RJ, CELIS MM, et al. Effects of a 12-wk resistance exercise program on skeletal muscle strength in children with burn injuries. *Journal of Applied Physiology* 2001; **91**(3): 1168-1175.  633. SUMAN OE, SPIES RJ, CELIS MM, et al. Effects of a 12-wk resistance exercise program on skeletal muscle strength in children with burn injuries. *Journal of applied physiology (Bethesda, Md : 1985)* 2001; **91**(3): 1168-1175.  634. SUMAN OE, THOMAS SJ, WILKINS JP, et al. Effect of exogenous growth hormone and exercise on lean mass and muscle function in children with burns. *Journal of applied physiology (Bethesda, Md : 1985)* 2003; **94**(6): 2273‐2281.  635. SUMAN OE, THOMAS SJ, WILKINS JP, et al. Effect of exogenous growth hormone and exercise on lean mass and muscle function in children with burns. *Journal of Applied Physiology* 2003; **94**(6): 2273-2281.  636. SUMAN OE, THOMAS SJ, WILKINS JP, et al. Effect of exogenous growth hormone and exercise on lean mass and muscle function in children with burns. *Journal of applied physiology (Bethesda, Md : 1985)* 2003; **94**(6): 2273-2281.  637. SUN ZW, YAN L, G YY, et al. Increasing dietary vitamin D3 improves the walking ability and welfare status of broiler chickens reared at high stocking densities. *Poultry science* 2013; **92**(12): 3071-3079.  638. SVEEN J, ANDERSSON G, BUHRMAN B, et al. Internet-based information and support program for parents of children with burns: A randomized controlled trial. *Burns : journal of the International Society for Burn Injuries* 2017; **43**(3): 583-591.  639. SVEEN J, ANDERSSON G, BUHRMAN B, et al. Internet-based information and support program for parents of children with burns: A randomized controlled trial. *Burns : journal of the International Society for Burn Injuries* 2017; **43**(3): 583-591.  640. SVEEN J, ANDERSSON G, BUHRMAN B, et al. Internet-based information and support program for parents of children with burns: A randomized controlled trial. *Burns : journal of the International Society for Burn Injuries* 2017; **43**(3): 583-591.  641. SWART L, VAN NIEKERK A, SEEDAT M, et al. Paraprofessional home visitation program to prevent childhood unintentional injuries in low-income communities: a cluster randomized controlled trial. *Injury Prevention* 2008; **14**(3): 164-169.  642. TAJIKI I, VIZESHFAR F, KESHTKARAN Z. The effect of training program based on health belief model on burn prevention knowledge in mothers of children aged to 1-3 years: A randomized controlled. *Burns : journal of the International Society for Burn Injuries* 2022; **48**(4): 808-815.  643. TAN J, FU JF. [Effects of pressure therapy on the proliferation and apoptosis of cells in hypertrophic scar of burn patients]. *Zhonghua Shao Shang Za Zhi* 2013; **29**(6): 509-515.  644. TENFORDE AS, HEFNER JE, KODISH-WACHS JE, et al. Telehealth in Physical Medicine and Rehabilitation: A Narrative Review. *Pm&R* 2017; **9**(5): S51-S58.  645. TERAN-WODZINSKI P, HALADAY D, VU T, et al. Assessing gait, balance, and muscle strength among breast cancer survivors with chemotherapy-induced peripheral neuropathy (CIPN): study protocol for a randomized controlled clinical trial. *Trials* 2022; **23**(1): 13.  646. THOMBS BD, BRESNICK MG, MAGYAR-RUSSELL G, et al. Symptoms of depression predict change in physical health after burn injury. *Burns : journal of the International Society for Burn Injuries* 2007; **33**(3): 292-298.  647. TOKGöZ P, WäHNERT D, ELSNER A, et al. Virtual Reality for Upper Extremity Rehabilitation-A Prospective Pilot Study. *Healthcare* 2023; **11**(10): 18.  648. TOOLAROUD PB, NABOVATI E, AKBARI H, et al. Evaluation of the effectiveness of a smartphone-based educational intervention on the outcomes of children's burns: A randomized controlled trial. *International Wound Journal* 2024; **21**(1): 8.  649. TREDGET EE, SHANKOWSKY HA, PANNU R, et al. Transforming growth factor-beta in thermally injured patients with hypertrophic scars: effects of interferon alpha-2b. *Plastic and reconstructive surgery* 1998; **102**(5): 1317-1328; discussion 1329-1330.  650. TRICCAS LT, BURRIDGE JH, HUGHES AM, et al. A qualitative study exploring views and experiences of people with stroke undergoing transcranial direct current stimulation and upper limb robot therapy. *Top Stroke Rehabil* 2018; **25**(7): 480-488.  651. TROPEZ-ARCENEAUX LL, MEYER WJ, HOFFMAN HG, et al. Pain and perceived exertion in pediatric burn patients during exercise with virtual reality. *Journal of Burn Care and Research* 2011; **32**: S66.  652. UNAL E, OZDEMIR A. The effect of hybrid simulated burn care training on nursing students' knowledge, skills, and empathy: A randomised controlled trial. *Nurse Education Today* 2023; **126**: 7.  653. VALKENBORGHS S, HILLMAN CH, AL-IEDANI O, et al. Effect of high-intensity interval training on hippocampal metabolism in older adolescents. *Journal of Science and Medicine in Sport* 2022; **25**: S62.  654. VALKENBORGHS SR, HILLMAN CH, AL-IEDANI O, et al. Effect of high-intensity interval training on hippocampal metabolism in older adolescents. *Psychophysiology* 2022; **59**(11): e14090.  655. VALKENBORGHS SR, HILLMAN CH, AL-IEDANI O, et al. Effect of high-intensity interval training on hippocampal metabolism in older adolescents. *Psychophysiology* 2022; **59**(11): e14090.  656. VALKENBORGHS SR, HILLMAN CH, AL-IEDANI O, et al. Effect of high-intensity interval training on hippocampal metabolism in older adolescents. *Psychophysiology* 2022; **59**(11): 13.  657. VAN BEELEN MEJ, BEIRENS TMJ, DEN HERTOG P, et al. Effectiveness of Web-Based Tailored Advice on Parents' Child Safety Behaviors: Randomized Controlled Trial. *J Med Internet Res* 2014; **16**(1): 19.  658. VAN VOORHEES AS, FRIED R. Depression and Quality of Life in Psoriasis. *Postgrad Med* 2009; **121**(4): 154-161.  659. VASILEIADIS GI, BALTA AA, ZERVA A, et al. Role of Kinesiotherapy in the Prevention of Heterotopic Ossification A Systematic Review. *American Journal of Physical Medicine & Rehabilitation* 2023; **102**(2): 110-119.  660. VESHNAVEI HA. Evaluation of the efficacy of Agicoat in the treatment of partial-thickness skin graft donor sites of burn patients. *International Journal of Burns and Trauma* 2021; **11**(6): 470-476.  661. VIERA AJ, ANTONELLI R. Potential effect of physical activity calorie equivalent labeling on parent fast food decisions. *Pediatrics* 2015; **135**(2): e376-e382.  662. VIERA AJ, ANTONELLI R. Potential effect of physical activity calorie equivalent labeling on parent fast food decisions. *Pediatrics* 2015; **135**(2): e376-382.  663. VLADIMIROVA OV, LAVRESHIN PM, MINAEV SV, et al. Treatment of burn wounds using chitosan wound dressings. *Clinical and Experimental Surgery* 2023; **11**(3): 99-104.  664. VOIGT CD, FONCERRADA G, PEñA R, et al. Effects of Community-Based Exercise in Adults With Severe Burns: a Randomized Controlled Trial. *Archives of physical medicine and rehabilitation* 2020; **101**(1S): S36‐S41.  665. VOIGT CD, FONCERRADA G, PEñA R, et al. Effects of Community-Based Exercise in Adults With Severe Burns: A Randomized Controlled Trial. *Archives of physical medicine and rehabilitation* 2020; **101**(1): S36-S41.  666. VOIGT CD, FONCERRADA G, PEñA R, et al. Effects of Community-Based Exercise in Adults With Severe Burns: A Randomized Controlled Trial. *Archives of physical medicine and rehabilitation* 2020; **101**(1s): S36-s41.  667. VOIGT CD, FONCERRADA G, PEñA R, et al. Effects of Community-Based Exercise in Adults With Severe Burns: A Randomized Controlled Trial. *Archives of physical medicine and rehabilitation* 2020; **101**(1): S36-S41.  668. VOON K, SILBERSTEIN I, ERANKI A, et al. Xbox Kinect™ based rehabilitation as a feasible adjunct for minor upper limb burns rehabilitation: a pilot RCT. *Burns : journal of the International Society for Burn Injuries* 2016; **42**(8): 1797‐1804.  669. VOON K, SILBERSTEIN I, ERANKI A, et al. Xbox Kinect™ based rehabilitation as a feasible adjunct for minor upper limb burns rehabilitation: a pilot RCT. *Burns : journal of the International Society for Burn Injuries* 2016; **42**(8): 1797‐1804.  670. VOON K, SILBERSTEIN I, ERANKI A, et al. Xbox Kinect™ based rehabilitation as a feasible adjunct for minor upper limb burns rehabilitation: A pilot RCT. *Burns : journal of the International Society for Burn Injuries* 2016; **42**(8): 1797-1804.  671. VOON K, SILBERSTEIN I, ERANKI A, et al. Xbox Kinect™ based rehabilitation as a feasible adjunct for minor upper limb burns rehabilitation: A pilot RCT. *Burns : journal of the International Society for Burn Injuries* 2016; **42**(8): 1797-1804.  672. VOON K, SILBERSTEIN I, ERANKI A, et al. Xbox Kinect™ based rehabilitation as a feasible adjunct for minor upper limb burns rehabilitation: A pilot RCT. *Burns : journal of the International Society for Burn Injuries* 2016; **42**(8): 1797-1804.  673. WAITE E, JENKINSON E, KERSHAW S, et al. Psychosocial Interventions for Children and Young People With Visible Differences Resulting From Appearance-Altering Conditions, Injury, or Treatment Effects: An Updated Systematic Review. *Journal of Pediatric Psychology* 2024; **49**(1): 77-88.  674. WALKOSZ BJ, BULLER DB, ANDERSEN PA, et al. Translation of a Ski School Sun Safety Program to North American Ski and Snowboard Schools. *Health promotion practice* 2015; **16**(4): 560-570.  675. WALSH NP, BLANNIN AK, BISHOP NC, et al. Effect of oral glutamine supplementation on human neutrophil lipopolysaccharide-stimulated degranulation following prolonged exercise. *Int J Sport Nutr Exerc Metab* 2000; **10**(1): 39-50.  676. WANG A, LV G, CHENG X, et al. Guidelines on multidisciplinary approaches for the prevention and management of diabetic foot disease (2020 edition). *Burns and Trauma* 2020; **8**.  677. WANG AP, LV GZ, CHENG XB, et al. Guidelines on multidisciplinary approaches for the prevention and management of diabetic foot disease (2020 edition). *Burns & trauma* 2020; **8**: 52.  678. WANG E, THOMAS JJ, RODRIGUEZ ST, et al. Virtual reality for pediatric periprocedural care. *Curr Opin Anesthesiol* 2021; **34**(3): 284-291.  679. WASIAK J, MCMAHON M, DANILLA S, et al. Measuring common outcome measures and their concepts using the International Classification of Functioning, Disability and Health (ICF) in adults with burn injury: A systematic review. *Burns : journal of the International Society for Burn Injuries* 2011; **37**(6): 913-924.  680. WEHBE J, SAFAR Y. Hypnosis and physiotherapy. *Kinesitherapie* 2015; **15**(168): e1-e10.  681. WEHBE J, SAFAR Y. Hypnosis and physiotherapy. *Kinesitherapie* 2015.  682. WEICHMANN F, AVALTRONI F, BURKI C. Review of Clinical Effects and Presumed Mechanism of Action of the French Oak Wood Extract Robuvit. *J Med Food* 2021; **24**(9): 897-907.  683. WEINTRAUB MI, WOLFE GI, BAROHN RA, et al. Static magnetic field therapy for symptomatic diabetic neuropathy: A randomized, double-blind, placebo-controlled trial. *Archives of physical medicine and rehabilitation* 2003; **84**(5): 736-746.  684. WEISZ JR, UGUETO AM, HERREN J, et al. When the torch is passed, does the flame still burn? Testing a "train the supervisor" model for the child STEPs treatment program. *Journal of Consulting and Clinical Psychology* 2018; **86**(9): 726-737.  685. WEISZ JR, UGUETO AM, HERREN J, et al. When the torch is passed, does the flame still burn? Testing a "train the supervisor" model for the Child STEPs treatment program. *J Consult Clin Psychol* 2018; **86**(9): 726-737.  686. WEISZ JR, UGUETO AM, HERREN J, et al. When the Torch Is Passed, Does the Flame Still Burn? Testing a "Train the Supervisor" Model for the Child STEPs Treatment Program. *Journal of Consulting and Clinical Psychology* 2018; **86**(9): 726-737.  687. WENDER CLA, AHN SJ, O'CONNOR PJ. Interactive Virtual Reality Reduces Quadriceps Pain during High-Intensity Cycling. *Medicine and science in sports and exercise* 2019; **51**(10): 2088-2097.  688. WHAYNE TF, MAULIK N. Nutrition and the healthy heart with an exercise boost. *Canadian Journal of Physiology and Pharmacology* 2012; **90**(8): 967-976.  689. WIDIANINGSIH NPS, PRAKOESWA CRS. Fractional laser and laser assisted corticosteroid delivery for hypertrophic scars in thermal burns. *Dermatology Reports* 2019; **11**(S1): 134-135.  690. WIECHMAN SA, CARROUGHER GJ, ESSELMAN PC, et al. A randomized controlled trial to test an expanded delivery model for patients with burn injuries. *Journal of Burn Care and Research* 2014; **35**: S79.  691. WIECHMAN SA, CARROUGHER GJ, ESSELMAN PC, et al. An expanded delivery model for outpatient burn rehabilitation. *Journal of Burn Care and Research* 2015; **36**(1): 14-22.  692. WIECHMAN SA, CARROUGHER GJ, ESSELMAN PC, et al. An expanded delivery model for outpatient burn rehabilitation. *Journal of burn care & research : official publication of the American Burn Association* 2015; **36**(1): 14-22.  693. WIECHMAN SA, CARROUGHER GJ, ESSELMAN PC, et al. An Expanded Delivery Model for Outpatient Burn Rehabilitation. *Journal of Burn Care & Research* 2015; **36**(1): 14-22.  694. WILD J, SMITH KV, THOMPSON E, et al. A prospective study of pre-trauma risk factors for post-traumatic stress disorder and depression. *Psychological Medicine* 2016; **46**(12): 2571-2582.  695. WISCHMEYER PE, SUMAN OE, KOZAR R, et al. Role of anabolic testosterone agents and structured exercise to promote recovery in ICU survivors. *Curr Opin Crit Care* 2020; **26**(5): 508-515.  696. WITHAM MD, RAMAGE L, BURNS SL, et al. Trends in Function and Postdischarge Mortality in a Medicine for the Elderly Rehabilitation Center Over a 10-Year Period. *Archives of physical medicine and rehabilitation* 2011; **92**(8): 1288-1292.  697. WOLF SE, ARNOLDO BD. The year in burns 2011. *Burns : journal of the International Society for Burn Injuries* 2012; **38**(8): 1096-1108.  698. WON AS, BAILEY J, BAILENSON J, et al. Immersive Virtual Reality for Pediatric Pain. *Children-Basel* 2017; **4**(7): 15.  699. WON YH, CHO YS, JOO SY, et al. The effect of a pulmonary rehabilitation on lung function and exercise capacity in patients with burn: A prospective randomized single-blind study. *Journal of clinical medicine* 2020; **9**(7): 1-11.  700. WOOD KM, OLIVE B, LAVALLE K, et al. Dissimilar Physiological and Perceptual Responses Between Sprint Interval Training and High-Intensity Interval Training. *Journal of strength and conditioning research / National Strength & Conditioning Association* 2016; **30**(1): 244-250.  701. WOOD KM, OLIVE B, LAVALLE K, et al. Dissimilar Physiological and Perceptual Responses Between Sprint Interval Training and High-Intensity Interval Training. *J Strength Cond Res* 2016; **30**(1): 244-250.  702. WU JX, LIU QS, ZHOU XL, et al. Primary observation of therapeutic effect of collagenase on hypertrophic scar. *Chinese Journal of Clinical Rehabilitation* 2005; **9**(38): 168-169.  703. WU JY, HU YM, ZHU Y, et al. Systematic Review of Adverse Effects: A Further Step towards Modernization of Acupuncture in China. *Evid-based Complement Altern Med* 2015; **2015**: 19.  704. WU KP, CHEN P, RU TF, et al. [A prospective randomized controlled study on the effects of bicycle ergometer rehabilitation training on quadriceps and walking ability of patients with lower limb dysfunction caused by extensive burns]. *Zhonghua Shao Shang Yu Chuang Mian Xiu Fu Za Zhi* 2022; **38**(5): 447-453.  705. WU YT, CHEN KH, BAN SL, et al. Evaluation of leap motion control for hand rehabilitation in burn patients: An experience in the dust explosion disaster in Formosa Fun Coast. *Burns : journal of the International Society for Burn Injuries* 2019; **45**(1): 157-164.  706. WURZER P, VOIGT CD, CLAYTON RP, et al. Long-term effects of physical exercise during rehabilitation in patients with severe burns. *Surgery* 2016; **160**(3): 781-788.  707. WYCKOFF MH, SINGLETARY EM, SOAR J, et al. 2021 International Consensus on Cardiopulmonary Resuscitation and Emergency Cardiovascular Care Science With Treatment Recommendations Summary From the Basic Life Support; Advanced Life Support; Neonatal Life Support; Education, Implementation, and Teams; First Aid Task Forces; and the COVID-19 Working Group. *Resuscitation* 2021; **169**: 229-311.  708. WYCKOFF MH, SINGLETARY EM, SOAR J, et al. 2021 International Consensus on Cardiopulmonary Resuscitation and Emergency Cardiovascular Care Science With Treatment Recommendations: Summary From the Basic Life Support; Advanced Life Support; Neonatal Life Support; Education, Implementation, and Teams; First Aid Task Forces; and the COVID-19 Working Group. *Circulation* 2022; **145**(9): E645-E721.  709. XIANG H. VIRTUAL REALITY (VR) AND THE KID'S BRAIN: EMERGING EVIDENCE FOR BRAIN COGNITIVE FUNCTION REHAB AND NEURO PAIN MECHANISM OF VR. *IBRO Neuroscience Reports* 2023; **15**: S910-S911.  710. YANG S, QIU L, XIAO J, et al. The effects of resistance training on children with burns: a meta-analysis. *Pediatric surgery international* 2021; **37**(10): 1323-1332.  711. YANG S, QIU L, XIAO J, et al. The effects of resistance training on children with burns: a meta-analysis. *Pediatric surgery international* 2021; **37**(10): 1323-1332.  712. YANG X, LI R, ZHAI J, et al. Effects of early enteral nutrition in patients with severe burns: A systematic review. *Medicine (United States)* 2024; **103**(7): E37023.  713. YANG XJ, LI R, ZHAI J, et al. Effects of early enteral nutrition in patients with severe burns: A systematic review. *Medicine* 2024; **103**(7): 10.  714. YANG YH, KANG JW, JIANG TY, et al. Safety and efficacy of treating post-burn pathological scars with extracorporeal shock wave therapy: A meta-analysis of randomised controlled trials. *Wound Repair and Regeneration* 2022; **30**(5): 595-607.  715. YANG YP, WANG CJ, WANG JJ, et al. The Effects of an Activity Promotion System on active living in overweight subjects with metabolic abnormalities. *Obes Res Clin Pract* 2017; **11**(6): 718-727.  716. YOSHIDA A, YAMAMOTO M, LI-TSANG CWP, et al. A systematic review assessing the effectiveness of hand therapy programmes in adults with burns using the International Classification of Functioning, Disability and Health framework. *Nagoya journal of medical science* 2022; **84**(4): 689-704.  717. YOSHIDA A, YAMAMOTO M, LI-TSANG CWP, et al. A systematic review assessing the effectiveness of hand therapy programmes in adults with burns using the International Classification of Functioning, Disability and Health framework. *Nagoya Journal of Medical Science* 2022; **84**(4): 865-870.  718. YOSHIDA A, YAMAMOTO M, LI-TSANG CWP, et al. A systematic review assessing the effectiveness of hand therapy programmes in adults with burns using the International Classification of Functioning, Disability and Health framework. *Nagoya Journal of Medical Science* 2022; **84**(4): 689-704.  719. YOUNG A, BROOKES S, RUMSEY N, et al. Agreement on what to measure in randomised controlled trials in burn care: study protocol for the development of a core outcome set. *Bmj Open* 2017; **7**(6): 8.  720. ZAHO JC, YU JA, XIAN CJ, et al. Burns Induced by Cupping Therapy in a Burn Center in Northeast China. *Wounds-a Compendium of Clinical Research and Practice* 2014; **26**(7): 214-220.  721. ZAL M, DELDAR K, FROUTAN R, et al. Rehabilitation of Burn Victims: Improving Quality of Life in Victims With Face and Neck Burn Through an Augmented Reality Coupled Pamphlet. *Journal of burn care & research : official publication of the American Burn Association* 2023; **44**(2): 311-319.  722. ZAL M, DELDAR K, FROUTAN R, et al. Rehabilitation of Burn Victims: Improving Quality of Life in Victims With Face and Neck Burn Through an Augmented Reality Coupled Pamphlet. *Journal of burn care & research : official publication of the American Burn Association* 2023; **44**(2): 311-319.  723. ZAL M, DELDAR K, FROUTAN R, et al. Rehabilitation of Burn Victims: Improving Quality of Life in Victims With Face and Neck Burn Through an Augmented Reality Coupled Pamphlet. *Journal of Burn Care & Research* 2023; **44**(2): 311-319.  724. ZHAO HY, HAN JT, HU DH, et al. A randomized controlled trial on the effect of exercise prescription based on a progressive mode in treating elderly patients with lower limb dysfunction after deep burns. *Zhonghua shao shang yu chuang mian xiu fu za zhi* 2023; **39**(12): 1122‐1130.  725. ZHAO HY, HAN JT, HU DH, et al. [A randomized controlled trial on the effect of exercise prescription based on a progressive mode in treating elderly patients with lower limb dysfunction after deep burns]. *Zhonghua Shao Shang Yu Chuang Mian Xiu Fu Za Zhi* 2023; **39**(12): 1122-1130.  726. ZHAO HY, LIU JQ, HAN JT, et al. [A prospective randomized controlled study on the effects of progressive core muscle group training combined with lower limb intelligent rehabilitation training for burn patients with lower limb dysfunction]. *Zhonghua Shao Shang Yu Chuang Mian Xiu Fu Za Zhi* 2022; **38**(12): 1117-1125.  727. ZHOU YM, ZOU YM, LI XC, et al. Lung Function and Incidence of Chronic Obstructive Pulmonary Disease after Improved Cooking Fuels and Kitchen Ventilation: A 9-Year Prospective Cohort Study. *PLos Med* 2014; **11**(3): 11.  728. ZHU C, HE L, ZHANG BW, et al. [Exploration of family rehabilitation model for children with scar contracture after hand burns]. *Zhonghua Shao Shang Yu Chuang Mian Xiu Fu Za Zhi* 2023; **39**(1): 45-52. |
