# Supplementary material for: The effect of physical exercise on cardiopulmonary fitness in burn patients: A meta-analysis
Source: PLoS One. 2025 Aug 18;20(8):e0330301. doi: 10.1371/journal.pone.0330301 (PMC12360598; doi:10.1371/journal.pone.0330301)
Supplement: S4 Appendix — (DOC) [file pone.0330301.s004.doc]

**S4 Table.** List of Included and Excluded Studies.

| **Included and excluded reasons** | **No. studies** | **References.** |
| --- | --- | --- |
| Included studies | 13 | [1-13] |
| Irrelevant articles | 180 | [14-193] |
| Inappropriate intervention | 73 | [194-266] |
| Not related to inclusion criteria | 25 | [267-291] |
| Response to Letter | 3 | [292-294] |
| Drug interventions | 40 | [295-334] |
| Review articles, conference articles | 32 | [335-366] |
| Without appropriate outcome | 49 | [367-415] |
| Systematic review | 22 | [416-437] |
| Study protocol | 20 | [438-457] |
| Animal study | 4 | [458-461] |
| Case study | 3 | [462-464] |
| Meta analysis | 14 | [465-478] |
| No abstract | 6 | [479-484] |
| Not original article | 2 | [485, 486] |
| Unable to extract data | 3 | [487-489] |

**REFERENCES**
